# Synergizing a knowledge graph and large language model for relay catalysis pathway recommendation

**DOI:** 10.1093/nsr/nwaf271

**Published:** 2025-07-14

**Authors:** Fei Fu, Qing-Qing Li, Fangrong Wang, Jie Hu, Tian-Tian Wang, Yun-Pei Liu, Weihong Xu, Zhili Lin, Fu-Qiang Gong, Qi-Yuan Fan, Jeff Z Pan, Ye Wang, Jun Cheng

**Affiliations:** State Key Laboratory of Physical Chemistry of Solid Surface, iChEM, College of Chemistry and Chemical Engineering, Xiamen University, Xiamen 361005, China; State Key Laboratory of Physical Chemistry of Solid Surface, iChEM, College of Chemistry and Chemical Engineering, Xiamen University, Xiamen 361005, China; School of Informatics, The University of Edinburgh, Edinburgh EH8 9AB, UK; Institute of Artificial Intelligence, Xiamen University, Xiamen 361005, China; Institute of Artificial Intelligence, Xiamen University, Xiamen 361005, China; State Key Laboratory of Physical Chemistry of Solid Surface, iChEM, College of Chemistry and Chemical Engineering, Xiamen University, Xiamen 361005, China; Laboratory of AI for Electrochemistry (AI4EC), Tan Kah Kee Innovation Laboratory (IKKEM), Xiamen 361100, China; Laboratory of AI for Electrochemistry (AI4EC), Tan Kah Kee Innovation Laboratory (IKKEM), Xiamen 361100, China; State Key Laboratory of Physical Chemistry of Solid Surface, iChEM, College of Chemistry and Chemical Engineering, Xiamen University, Xiamen 361005, China; State Key Laboratory of Physical Chemistry of Solid Surface, iChEM, College of Chemistry and Chemical Engineering, Xiamen University, Xiamen 361005, China; School of Chemistry and Chemical Engineering, Shanxi University, Taiyuan 030006, China; School of Informatics, The University of Edinburgh, Edinburgh EH8 9AB, UK; State Key Laboratory of Physical Chemistry of Solid Surface, iChEM, College of Chemistry and Chemical Engineering, Xiamen University, Xiamen 361005, China; State Key Laboratory of Physical Chemistry of Solid Surface, iChEM, College of Chemistry and Chemical Engineering, Xiamen University, Xiamen 361005, China; Institute of Artificial Intelligence, Xiamen University, Xiamen 361005, China; Laboratory of AI for Electrochemistry (AI4EC), Tan Kah Kee Innovation Laboratory (IKKEM), Xiamen 361100, China

**Keywords:** relay catalysis, knowledge graph, large language model, generative pre-trained transformer

## Abstract

Relay catalysis integrates multiple catalytic reactions to efficiently transform intermediates and enhance conversion and selectivity. However, designing these pathways and multifunctional catalysts is often lengthy and costly, heavily relying on in-depth literature analysis by experienced researchers. To address this, we developed an approach that combines a knowledge graph (KG) and large language models (LLMs) to automatically recommend multistep catalytic reaction pathways. Our method involves using an LLM-assisted workflow for data acquisition and organization, followed by the construction of a detailed catalysis knowledge graph (Cat-KG). After querying the Cat-KG, promising relay catalysis pathways are identified by applying scoring rules informed by expertise in relay catalysis. The LLM then transforms the structured pathways and reaction condition data into readable chemical equations and descriptions for chemists. This step integrates catalysis knowledge from the Cat-KG and helps avoid LLM-induced hallucinations by using reliable information. The method efficiently recommended relay catalysis pathways for ethylene, ethanol, 2,5-furandicarboxylate and other targets within minutes, identifying pathways consistent with reported ones while using different reaction conditions, validating its effectiveness. Thus, this strategy can extrapolate known and novel relay catalysis pathways, showcasing its potential for application in pathway selection.

## INTRODUCTION

Relay catalysis, a strategy that integrates multiple catalytic reactions into a single, multifunctional system, has gained attention in the field of catalysis [1–5]. This approach boosts thermodynamic and kinetic efficiencies in multistep processes through simultaneous co-catalysis, outperforming traditional single-function catalysts in terms of synthesis efficiency, selectivity and atom economy, while reducing costs, optimizing energy and enhancing environmental sustainability [2]. Relay catalysis has shown promising results in fields like Fischer-Tropsch synthesis [6,7], where the design concept of oxide-zeolite composite catalysts has overcome selectivity limitations imposed by the Anderson–Schulz–Flory distribution [5], achieving remarkable selectivity in the direct conversion of syngas to mixed light olefins [8], ethylene [1] and ethanol [9,10]. However, the process of identifying individual catalytic steps and combining them in a coordinated manner, along with determining the optimal reaction conditions, is still a lengthy and challenging endeavor [10]. This complexity arises from three main challenges: the dispersion of relevant knowledge and data across various sources, which complicates data collection; the inefficiency of human analysis in matching catalysts and reaction conditions, which makes the process time-consuming and subjective; the complexity of validating proposed pathways, which requires costly and comprehensive experimental efforts. These challenges make relay catalysis research both demanding and resource-intensive.

Knowledge graphs (KGs) emerge as a promising tool to address these challenges [11]. In KGs, information is represented as a network of interconnected nodes and edges, where nodes represent entities (such as reactants, catalysts, solvents and products), and edges depict the relationships between these entities. With their unique structure of nodes representing entities and edges depicting relationships, KGs have proven successful in various fields [12,13]. Grzybowski and co-workers have utilized networks to encapsulate knowledge in organic reactions and molecular structures, mapping expansive networks of chemical reactions [14–16]. These networks, which can be considered as precursors to modern KGs, facilitated the identification of key molecules and the discovery of synthetic pathways, including pathways optimized for one-pot synthesis [17]. KGs have also been applied to catalyst prediction and reaction condition optimization in organic reactions, demonstrating their unique potential in supporting chemical reaction design [18]. KGs can be tailored to address specific challenges in relay catalysis by offering advantages in three areas: (i) efficiently consolidating dispersed data from various resources, e.g. literature, books and chemists’ experiential knowledge [19]; (ii) simplifying complex aspects of relay catalysis into a coherent knowledge base, addressing inefficiencies in manual analysis [16,19–21]; (iii) supporting reasoning [11,19,22] and predictive tasks, aligning with the complexities in pathway validation, thus reducing time and resources required in experimental validation. Compared to static relational databases and flat data storage systems, KGs offer dynamic adaptability and semantic richness, enabling them to evolve with new information [11,21,23]. Therefore, we propose constructing a KG tailored specifically for the intricate aspects of relay catalysis to provide a strategic tool for exploring and identifying effective catalytic reaction pathways and conditions.

Constructing a KG involves several steps, including schema design, data collection, data cleaning and knowledge fusion [24,25], and knowledge updating [11,21,23]. Selecting appropriate data sources is crucial for the quality of the KG. Existing databases relevant to chemistry, catalysis and materials science, such as the Open Reaction Database [26,27], Reaxys [28] and SciFinder [29], were assessed. While these resources offer high-quality data and extensive information on substance transformations, selectivity yields and reaction conditions, they lack detailed information on catalyst synthesis methods and the physical and chemical properties of catalysts. This gap is particularly significant in relay catalysis, where detailed catalyst information is essential for designing multifunctional catalysts and generating effective pathways. To bridge these information gaps, we propose collecting in-depth, reliable data on catalytic reactions from high-quality scientific publications to construct a KG. This method aims to enrich the knowledge base with precise experimental conditions and catalyst specifics, enhancing data accuracy and integrity. The KG approach offers several advantages, such as integrating real-time updates [11], storing reaction information in a structured manner [21] and enabling advanced reasoning capabilities [19,30]. Implementing this approach facilitates the construction of a high-quality catalysis KG, thereby bolstering relay catalysis research.

Extracting high-quality catalytic reaction data from scientific literature presents multiple challenges. These challenges include the lack of standardization and organization due to various formats and methods employed by different research groups and publications. Additionally, the wide range of reaction types and conditions complicates data extraction, and a deep understanding of chemistry is needed to accurately distinguish between target reactions and similar reactions [31,32]. Traditional data acquisition relies on pre-trained language models (PLMs) [31,33] for tasks such as named entity recognition, relationship extraction and text classification. However, this process depends on manual annotation, pre-training and fine-tuning of models, leading to low efficiency, strong subjectivity and challenges in ensuring quality knowledge extraction, while also requiring extensive programming and data science knowledge [31]. In contrast, the emergence of advanced large language models (LLMs) such as generative pre-trained transformers (GPTs)[34], Claude3.5, Gemini [35] and GPT-4 [36] marks a significant shift in the era of AI-generated content. These models possess robust general knowledge capabilities and an understanding of domain-specific content [21,37], demonstrating potential in simplifying the extraction of chemical data [38,39]. Unlike PLMs, LLMs can utilize semantic understanding and generation to extract knowledge, improving the accuracy and coverage of knowledge extraction, while significantly reducing the time and effort required for data annotation, making it possible to construct a high-quality, large-scale catalysis KG at low cost [21,30].

**Figure 1.**
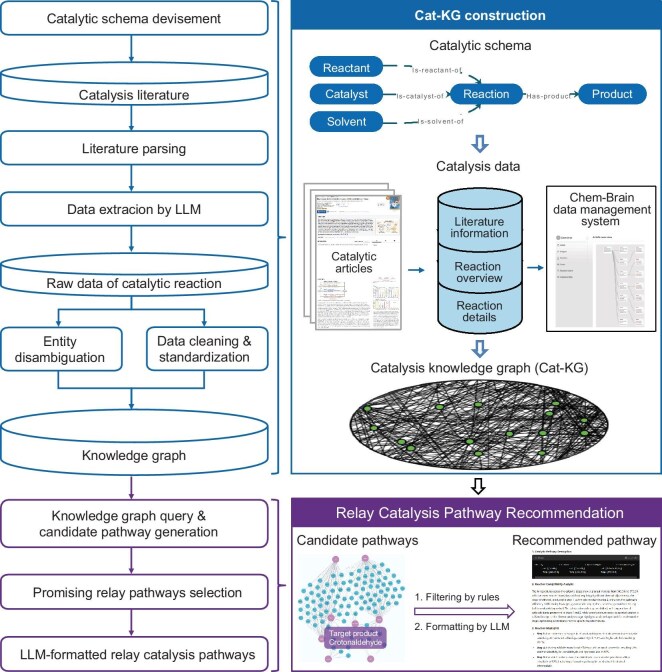
Workflow diagram of the construction of the Cat-KG and application for relay catalysis. The initial phase involves the selection of a catalytic schema and the acquisition of data from catalysis-centric literature to establish the Cat-KG (blue), followed by the recommendation of a reaction pathway (purple). Shared steps are marked with the corresponding color.

In this research, we present a workflow that combines the strengths of a KG and an LLM to efficiently recommend relay catalysis pathways. Our approach consists of two main phases: constructing the catalysis knowledge graph (Cat-KG) and using it to recommend reaction pathways (Fig. 1). For the Cat-KG construction, we use Gemini’s data extraction abilities to gather comprehensive catalytic reaction data from literature. The resulting Cat-KG covers 15 881 publications and 27 760 thermocatalytic reactions, of which 18 174 are heterogeneous catalysis reactions. We then use the Cat-KG to identify promising relay catalysis pathways by searching through it and applying scoring rules to filter out high-quality pathways and their key reaction information from numerous candidates obtained. Then, GPT-4 is used to convert the structured text into natural language descriptions and easily readable chemical equations. This KG-enhanced LLM approach reduces hallucinations, ensures recommendation effectiveness and maintains traceability to the original literature. Our method recommended reaction pathways for ethylene, ethanol and 2,5-furandicarboxylate, including pathways consistent with reported literature, using different reaction conditions, and achieving results significantly faster than manual efforts. This strategy enhances the exploration of reaction pathways by combining expert-designed scoring rules, KG and LLM, enabling chemists to efficiently identify known effective pathways and discover new relay catalysis pathways.

## RESULTS AND DISCUSSION

### Framework construction

Our workflow harnesses the combined strengths of KGs and LLMs to efficiently generate and evaluate relay catalysis pathways, as shown in Fig. 1. We begin by constructing the Cat-KG using an LLM-assisted methodology to accurately gather and structure catalytic reaction data from the literature, transforming scattered information into a coherent catalysis database. The Cat-KG provides detailed, traceable and structured catalytic reaction information. Next, the Cat-KG is used to search for potential pathways. Manually designed scoring rules are applied to filter and recommend high-quality paths, which are then output as structured and organized information. LLMs are then employed to convert the structured recommended relay catalysis pathway information into a more accessible natural language format, ensuring both accuracy and readability. This workflow highlights the unique capabilities of both KGs and LLMs, demonstrating how their integration can advance catalysis research by streamlining the data collection and analysis process.

### Knowledge graph construction

KGs can be constructed using two main methodologies: top down and bottom up [40]. The top-down method starts by defining a schema with high-level concepts, which are then refined into a detailed taxonomy and linked with specific entities like catalytic reactions and catalysts. This method is particularly useful for building domain-specific KGs. Given the interconnected nature of reactants, catalysts, solvents and products, the top-down approach was selected for the construction of the Cat-KG. As illustrated in Fig. 1, the construction process involved schema design, data collection, cleaning, entity disambiguation, data storage and knowledge updating. These steps resulted in a comprehensive and accurate representation of catalytic reactions and catalysts.

**Figure 2.**
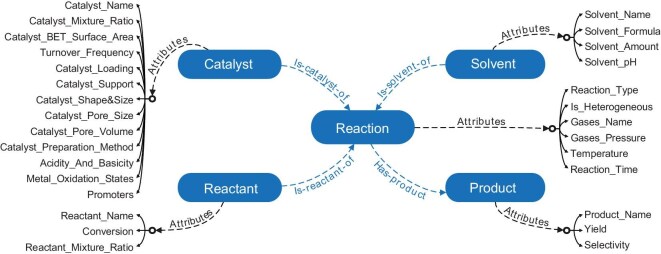
Schema of the Cat-KG. The catalytic reaction data structure utilized in this study encompasses five core schema classes and 29 unique attributes to thoroughly represent the complexity of catalytic processes.

To construct a detailed and professional KG in the catalysis domain, a comprehensive schema was developed to represent the highest-level concepts in this field. The schema was organized into a structured hierarchy consisting of five essential classes: reaction, reactant, catalyst, solvent and product. These classes are interconnected through four types of relationships, as illustrated in Fig. 2, and the schema encompasses 29 attributes. Each class and its relationships are closely linked, ensuring that all critical catalysis-related data are accurately captured. This well-defined schema serves as the foundation for the construction of the Cat-KG, allowing for the detailed representation and analysis of catalytic reactions within this structured framework.

**Figure 3.**
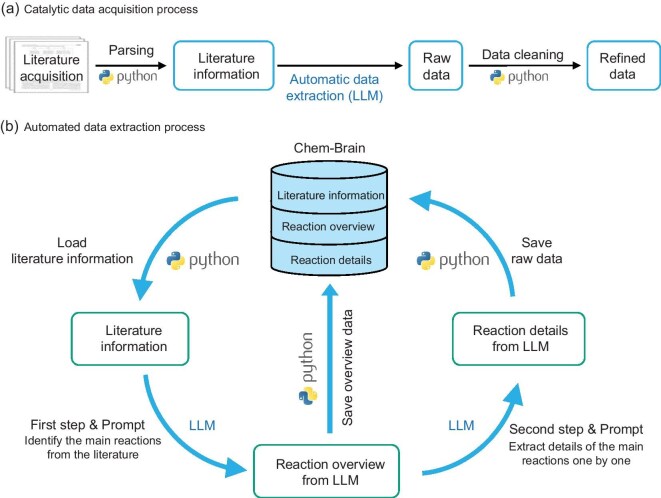
Workflow of catalysis data acquisition and automated extraction. Panel (a) outlines the process of extracting and validating structured data from the literature, employing a Python-based script for parsing and an LLM for ensuring the reliability of the data. Panel (b) delineates the automated sequence for extracting catalytic reaction data from literature information in the Chem-Brain system, which includes generating a reaction overview and reaction details via an LLM, followed by the data storage phase.

To obtain key catalytic reaction information, a workflow was designed to efficiently gather high-quality data, as shown in Fig. 3. Additionally, the Chem-Brain platform (see Fig. S48) was developed and implemented to manage the catalysis literature and their reaction data, facilitate data sharing among researchers and support interactive applications with the Cat-KG.

With the support of the Chem-Brain data management system, we collected, processed and stored data from peer-reviewed publications that contain comprehensive information on catalytic reactions. We gathered 15 881 publications related to thermal catalysis from reputable publishers (see the Methods section within the online supplementary material). We then designed a literature parsing process to automatically extract relevant information on catalysis quality and details from these publications. This included information such as publication date, journal name and citation count, which reflect the reliability and relevance of the reactions, as well as text from abstracts, introductions, main texts, figure captions and table notes, which provide specific reaction parameters.

To extract complete and high-quality catalytic reaction data from the literature, we developed a workflow as shown in Fig. 3. LLMs have demonstrated outstanding performance in general chemical knowledge understanding and reasoning-based question answering [41,42], chemical experiment planning and design [43–45], as well as in literature information integration and data extraction from chemical texts [21,38,46–48]. LLMs can gather a large amount of data more cost-effectively and efficiently than traditional manual annotation and named entity recognition techniques. However, previous reports in the chemical field usually extract data from single paragraphs [38,49]. This approach is not suitable for extracting detailed catalytic reaction data because it does not capture comprehensive information. For example, product yields are typically reported in the results section, while key information about the catalyst preparation method and catalyst support is found in the methods section. Therefore, single-paragraph extraction methods are insufficient for comprehensive data collection, prompting the need to explore full-text extraction.

Full-text extraction introduces additional challenges, primarily due to the decreased performance of LLMs with longer prompts. To overcome these issues, three strategies were implemented. First, a sequential extraction process was employed, dividing the task into two steps to reduce complexity and improve the results, as shown in Fig. 3b. Second, the Gemini 1.5 Flash model, which is well suited for this task, was utilized to improve the understanding of long texts and enhance extraction accuracy. Lastly, prompt engineering was applied to design reliable prompts that ensure consistency and completeness in the extracted data (details provided in the Methods section within the online supplementary material).

The automated catalysis data acquisition and extraction workflow shown in Fig. 3 was used to process 15 881 catalysis articles, resulting in the extraction of 27 760 thermocatalytic reactions, including 18 174 heterogeneous catalysis reactions. Thanks to the implementation of the full-text extraction strategy and the methods that improved its performance, the workflow was able to capture reactions with rich key information. The extracted literature information, reaction overviews and reaction details were first structured in JSON format (Figs S50 and S52) using automated scripts and then systematically stored and organized in the Chem-Brain platform.

To evaluate the effectiveness and quality of data extraction, a representative set of reactions was selected from the reaction pathways of 10 different compounds, resulting in 44 reactions, as detailed in Section S2 within the online supplementary material. By calculating and analyzing the cumulative precision (P) and recall (R) curves, we observed minimal fluctuations, indicating high stability. The calculated values of P, R and F1, along with their standard errors, were as follows: mean precision, 91.49% (SE 1.07%); mean recall, 91.18% (SE 1.04%); mean F1 score, 0.9113 (SE 0.0086). These results demonstrate that the data extraction strategy is effective, providing high-value data. The detailed data and the methods for calculating P, R and F1 can be found in Section S2 within the online supplementary material.

Despite the use of the advanced LLM, Gemini, some issues such as errors, omissions, duplicates and inconsistent formats were observed, which are common when dealing with large datasets [21,42]. To address these, a data cleaning and entity disambiguation process was implemented, incorporating a rule-based automated system to improve efficiency. This process significantly improved data quality, and the cleaned and disambiguated data were stored in structured JSON format, providing a foundation for the construction of the KG and subsequent search applications. In addition, the entire automation process is modular, allowing each component to be updated or replaced as needed. For example, as LLMs evolve rapidly, the system can switch to different LLM application programming interfaces to take advantage of newer models. Carefully designed prompts restrict the output format, data structure and key-value types, and robust parsing logic ensures consistency despite stylistic differences across models. These design choices support seamless LLM replacement without disrupting the Cat-KG construction pipeline.

To effectively transform the extracted data into a visual KG, Neo4j [40,50], a high-performance graph database designed to handle complex network relationships, was selected. Using this approach, data from 15 881 catalysis publications were successfully converted into a Neo4j-based KG (see the Methods section within the online supplementary material for a detailed construction). This KG not only improved data accessibility and visualization, but also provided a solid foundation for complex queries and analyses.

Currently, we have successfully established a comprehensive Cat-KG in the field of catalysis on Neo4j, adhering to the construction process outlined earlier (details in the Methods section within the online supplementary material). The Cat-KG is continuously updated and expanded with newly extracted catalytic reaction data through a dynamic update mechanism, which ensures that the KG remains up to date with the evolving body of literature. As part of this update process, a rule-based entity disambiguation procedure was implemented to resolve inconsistencies and ambiguities in chemical entities across different sources, thereby improving data quality, consistency and interoperability. The overall design, implementation strategies and advantages of the dynamic update mechanism, including the integrated entity disambiguation workflow, are described in detail in Section S3 within the online supplementary material.

At present, the Cat-KG encompasses 15 881 catalysis publications and 27 760 thermocatalytic reactions. The reaction information includes five schema classes, four types of relationships and 29 key catalytic attributes. Its extensive coverage of catalytic reactions provides detailed information for downstream applications, offering a wealth of catalysis knowledge and supporting logical reasoning. Moreover, the Cat-KG assists chemical researchers in querying and retrieving catalytic reaction information, with full traceability to the original literature sources. For instance, using the Chem-Brain platform (https://ai4ec.ikkem.com/apps/chembrain) users can search for catalytic chemical reactions based on catalysts, reactants and products. All data sources used to construct the Cat-KG were obtained through authorized and compliant means; for more details, see the LLM-Assisted Presentation of Relay Catalysis Pathway subsection under the Methods section within the online supplementary material. These values make it a valuable tool for exploring and analyzing catalysis.

### Relay catalysis application

**Figure 4.**
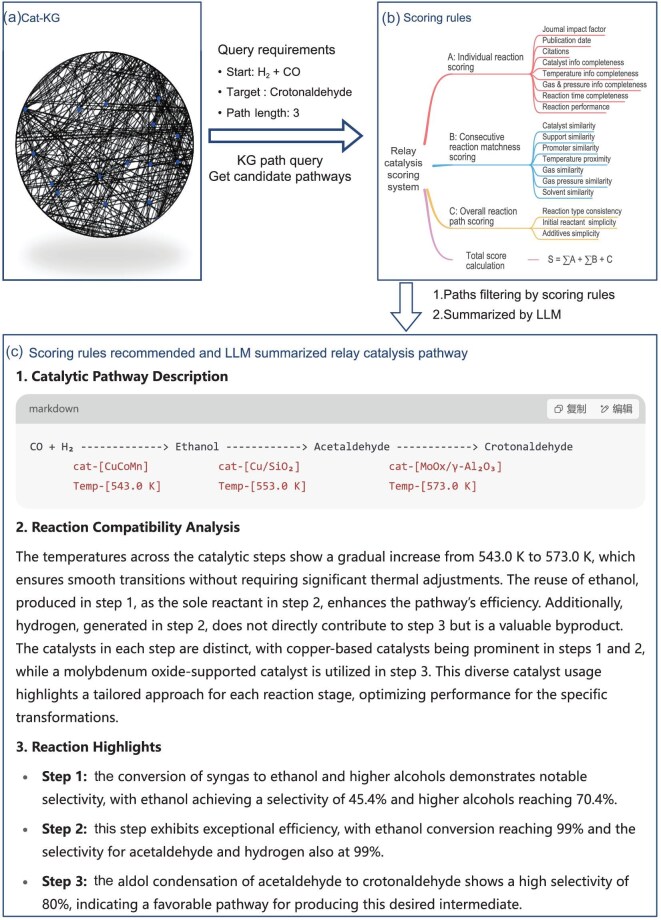
Relay catalysis pathway query, filtering and recommendation process using the Cat-KG. Panel (a) represents the schematic of the Cat-KG, where candidate pathways are generated via specific KG queries. Panel (b) represents the filtering and prioritizing of these pathways using scoring rules. Panel (c) presents the recommended relay catalysis pathways, summarized and formatted by an LLM for clarity.

To efficiently identify and recommend potential relay catalysis pathways, an automated recommendation system was developed using the Cat-KG (Fig. 4). The system is designed to suggest possible pathways and reaction conditions based on existing knowledge, serving as a foundation for further experimental exploration. Chemists can then use these recommendations as a starting point to refine and optimize reaction parameters through subsequent experiments.

Specifically, the workflow integrates the traceability of reaction information from the Cat-KG, the interpretability of chemistry-based filtering rules and the language processing capabilities of LLMs [21,30]. It automatically generates and executes Neo4j queries through Python scripts, ranks the identified pathways and processes the top-ranked results into structured JSON format. These structured data are then converted into natural language descriptions by an LLM, making the information more accessible for researchers.

First, Cypher, the query language used for interacting with graph databases such as Neo4j [50], is used to search the Cat-KG for candidate relay catalysis pathways based on a specified target product and pathway length. Users can also specify reaction conditions, starting materials or intermediates to refine the search. For example, when querying crotonaldehyde as the target product with H$_2$ and CO as starting materials and a pathway length limit of three steps (Fig. 4), the system can identify over 93 000 candidate pathways within a minute. To enhance usability, we developed a user-friendly query interface that allows users to specify pathway constraints (Fig. S57). The recommendation system automatically generates and executes the corresponding Cypher queries based on user input, enabling researchers to interact with the system without needing to learn Cypher syntax. This approach facilitates more precise searching and recommendation of expert-preferred relay catalysis pathways, while also improving the objectivity and customizability of the recommendation system.

However, many of the candidate pathways are impractical due to incompatibilities in reaction conditions. Issues such as large temperature differences or the presence of incompatible additives (for example, O$_2$ and H$_2$) in the same pathway are common. To address this, a set of scoring rules (Fig. 4b) was designed to evaluate the validity and feasibility of the pathways, based on our understanding and experience in relay catalysis design. These rules assess factors such as the reliability of the reaction sources, the compatibility of key reaction conditions and catalysts in adjacent steps, and the overall atom economy and reaction phase (details in the Methods section within the online supplementary material). A Python script computes scores for each pathway, filtering out only the top-ranked candidates that are research worthy for further study (Fig. S56).

Once the top pathways are identified, an LLM is employed to generate readable descriptions of the pathways in natural language. Using the pathway IDs, detailed information for each reaction step is traced from Chem-Brain. Since the original JSON format is difficult to interpret (Fig. S52), the LLM’s natural language generation capabilities are used to convert the structured data into readable chemical equations and descriptions. From various available LLMs, GPTs were chosen for this task, though other LLMs could also perform it. A tool, the *relay catalysis analyzer*, was developed within GPTs (Figs S3 and S7), which automatically formats the information into a clear and understandable structure. For example, the recommended pathway for crotonaldehyde (Fig. 4c) presents key conditions, a compatibility analysis of reactions and highlights for each reaction step. This process makes it easier for researchers to quickly understand the essential details of each pathway.

The pathway recommendation system developed in this study combines the traceability of reaction data from the Cat-KG, the interpretability of chemistry-based scoring rules and the language processing power of LLMs. This integration enables the system to quickly recommend high-value pathways aligned with specific goals. Unlike traditional reaction databases such as Reaxys [28] or SciFinder [29], which focus mainly on single-step reactions and molecular structure retrieval, our Cat-KG-based approach is naturally flexible and can be used in different types of catalytic applications. In this study, we focus on relay catalysis by adding customized workflows and evaluation methods to support the recommendation of multistep pathways. These include a scoring and filtering system that checks whether the catalysts and reaction conditions are suitable across different steps. Furthermore, the Cat-KG captures detailed information on catalyst synthesis and characterization—data often missing from existing resources, but essential for the development of multifunctional catalytic systems. Combined with the generative capabilities of LLMs, this structured knowledge enables the system to produce readable and traceable pathway summaries, helping chemists quickly understand, verify and explore new relay catalytic strategies.

While LLMs are good at summarizing and explaining chemical information in natural language, they still have limitations when dealing with complex chemistry [21,30,41,42]. These include bias from training data and limited access to structured, trustworthy chemical information. As a result, LLMs may produce content that appears correct, but may not be fully reliable, especially without access to trustworthy and structured data [21,42] (Section S4 within the online supplementary material). To reduce this problem, we provide the LLM with structured JSON data that includes detailed reaction information and IDs from the Cat-KG. This helps the model generate readable, traceable and more accurate descriptions. However, mistakes can still happen. So, for any pathways that researchers find valuable, the outputs are manually checked by tracing the IDs back to the original Cat-KG entries and related publications. This process ensures that the information is correct and supports further study. For more details, see Section S1. Methods, under the subsection LLM-Assisted Presentation of Relay Catalysis Pathway.

Based on the Cat-KG-based relay catalysis pathway recommendation system developed in this work, 20 new pathways were proposed for 10 valuable target compounds. These compounds include lower olefins, ethylene, ethylene glycol, oxalic acid, propylene glycol, crotonaldehyde, 1,3-butadiene, 1,4-butanediol, cis-2-butene and 2,5-furandicarboxylic acid (FDCA). These pathways have not yet been reported in the literature (see Section S5 within the online supplementary material). They provide new opportunities for future research on pathway optimization and experimental validation.

**Figure 5.**
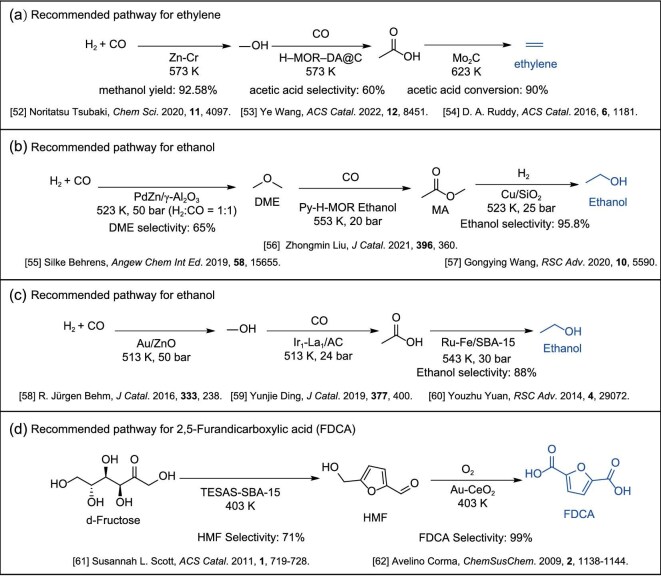
Relay catalysis pathways recommended by the KG-based recommendation system. Panel (a) displays a three-step pathway recommended for the synthesis of ethylene that is consistent with reported [1] relay catalysis pathways. Panels (b) and (c) display three-step pathways recommended for the synthesis of ethanol, consistent with reported [3,9] relay catalysis pathways. Panel (d) displays a two-step pathway recommended for the synthesis of FDCA, consistent with reported [51] relay catalysis pathways.

Moreover, we identified four relay catalysis pathways that have been reported and validated in the literature (Fig. 5). These include a three-step pathway for the synthesis of ethylene (Fig. 5a), which starts with methanol production from syngas [52], followed by the conversion of methanol and carbon monoxide into acetic acid [53], and ends with the hydrogenation of acetic acid to ethylene [54]. The reaction conditions for each step were obtained from high-quality catalysis journals, ensuring the pathway’s reliability. Wang and co-workers confirmed the effectiveness of this pathway, achieving 85% ethylene selectivity from the methanol intermediate [1]. We also identified two three-step pathways for the synthesis of ethanol (Fig. 5b and c). The first pathway involves the synthesis of ethanol from syngas via dimethyl ether (DME) [55], which is then converted to methyl acetate [56], and finally hydrogenated to ethanol [57]. The second pathway follows the sequence of syngas to methanol [58], methanol to acetic acid [59], and acetic acid to ethanol through hydrogenation [60]. Both have been experimentally validated as effective relay catalysis pathways [3,9]. Finally, we identified a two-step pathway for the synthesis of FDCA (Fig. 5d), which begins with the conversion of d-fructose to 5-hydroxymethylfurfural (HMF) [61], followed by the oxidation of HMF to FDCA [62]. This pathway has also been validated [51].

Taking the syngas-to-methanol conversion as an example (see Fig. 5 and Fig. S58), we discuss the effectiveness of the Cat-KG-based recommendation for relay catalysis pathways. The reported relay catalysis pathway [1] is compared with the Cat-KG-recommended three-step relay pathway from syngas to a methanol intermediate and finally to the target product ethylene. Both pathways share the same starting feedstock and intermediate, but the catalysts used in each step differ. In the methanol synthesis step, the Cat-KG-based recommendation system selects the well-established Zn–Cr oxide catalyst, which achieves a methanol yield of 92.6% at 573 K [52]. For the methanol carbonylation step, the Cat-KG recommends H-MOR-DA@C, which provides 60% acetic acid selectivity at 573 K [53], while the reported pathway uses H-MOR-DA [1]. In the acetic acid hydrogenation and decarboxylation step, the Cat-KG system adopts Mo$_2$C, achieving 90% conversion at 623 K [54], whereas the reported pathway employs ZnO–TiO$_2$ as the catalyst. Benefiting from the introduction of temperature similarity weighting in the recommendation system, all three steps are confined within a narrow temperature window of 573–623 K. This design enables each step to operate close to its optimal reaction condition and matches well with the reported pathway’s optimal relay catalysis temperature of 583 K [1].

Despite these achievements, several challenges remain in the integration of Cat-KG with GPT-4 for reaction pathway research. In the current work, each recommended reaction is treated independently. However, when combined into a full pathway, interactions between reaction conditions can influence overall catalytic performance [10]. Although the recommended pathways match reported ones in terms of feedstock, intermediates and products, the algorithm primarily focuses on selecting the best catalyst and conditions for individual steps. It does not yet account for cross-step factors such as acidity–metal activity matching, by-product management or hydrothermal stability. In contrast, the reported relay catalysis pathway from syngas to a methanol intermediate and finally to the target product ethylene [1] accounts for thermodynamic and kinetic coupling across multiple steps, as well as spatial distribution and compatibility of active sites, as shown in Fig. S58. For example, the hydrogenation of acetic acid produces a large amount of H$_2$O, which can poison the acid sites of the upstream catalyst H-MOR–DA, especially when water accumulates in confined spaces. The ZnO–TiO$_2$ catalyst used in the reported pathway enables a simultaneous water–gas shift reaction, i.e. H$_2$O + CO $\rightarrow$ H$_2$ + CO$_2$, which converts water into gas-phase products *in situ* and mitigates deactivation, thereby maintaining the carbonylation activity in the first step. Such cross-step coupling is essential for ensuring long-term catalyst stability and high product selectivity [1]. At present, there is no efficient and universal method to intelligently recommend or determine optimal conditions such as metal valence states, catalyst ratios, proportions, particle sizes and mixing strategies. Even the most advanced LLMs cannot accurately predict these parameters, making manual experimentation necessary to explore different catalyst combinations and reaction conditions in order to identify the optimal configuration.

Looking ahead, we plan to develop an intelligent AI-driven strategy that helps recommend optimal conditions for individual reactions within a pathway, while also considering how these conditions interact. To further improve the accuracy and practicality of pathway recommendations, we also plan to introduce reinforcement learning techniques, such as interactive reinforcement learning from human feedback [63], where expert feedback on pathway preferences can be used to optimize scoring weights through reinforcement learning, making the results better aligned with actual research needs. We will explore how to incorporate synergies and mutual influences between reaction conditions. Additionally, we plan to expand the Cat-KG beyond thermal catalysis by continuously incorporating data on photocatalysis and electrocatalysis. This will help build a comprehensive KG for the catalysis field, better supporting researchers in tasks such as reaction and literature retrieval, question answering and even more complex knowledge reasoning and prediction. As our KG grows, our data-driven recommendation strategy will become increasingly effective.

## CONCLUSIONS

We successfully developed an approach that combines a KG with LLMs to recommend relay catalysis pathways effectively. Our method used a Gemini-assisted workflow for extracting full-text data, resulting in structured, high-quality catalytic reaction and literature data. This enabled us to create a detailed Cat-KG, built from 15 881 catalysis literature sources and including 27 760 thermocatalytic reactions, which formed the basis of our pathway recommendation system. Using these extensive data, along with chemistry-based filtering rules and GPT-4’s understanding and natural language answering capabilities, our system quickly identified valuable relay catalysis pathways. This included four pathways that matched those reported in the literature and 20 previously unreported valuable pathways. Each pathway search and recommendation can be completed within minutes. This demonstrates that our KG and LLM-based approach can improve the efficiency of relay catalysis research and aid in discovering new reaction pathways. As we continue to expand the Cat-KG and leverage advanced AI technologies, we expect to assist chemists not only in thermal catalysis research, but also in photocatalytic and electrocatalytic applications.

## Supplementary Material

nwaf271_Supplemental_File

## Data Availability

The Cat-KG constructed in this work enables the retrieval of catalytic reaction information and data (https://ai4ec.ikkem.com/apps/chembrain). The data supporting the findings of this study are available within the article and its online supplementary  material, or from the authors upon reasonable request.
