# Supplementary material for: Synergizing a knowledge graph and large language model for relay catalysis pathway recommendation
Source: Natl Sci Rev. 2025 Jul 14;12(8):nwaf271. doi: 10.1093/nsr/nwaf271 (PMC12374726; doi:10.1093/nsr/nwaf271)
Supplement: nwaf271_Supplemental_File [file nwaf271_supplemental_file.pdf]

Supporting Information

Synergizing Knowledge Graph and Large Language Model for Relay Catalysis Pathway Recommendation

Fei Fu[1†], Qing-Qing Li[1†], Fangrong Wang[2], Jie Hu[3], Tian-Tian Wang[3], Yun-Pei Liu[1], Weihong Xu[4], Zhili Lin[4], Fu-Qiang Gong[1], Qi-Yuan Fan[1,5], Jeff Z. Pan[2*], Ye Wang[1*], Jun Cheng[1,3,4*]

[1]State Key Laboratory of Physical Chemistry of Solid Surface, iChEM, College of Chemistry and Chemical Engineering, Xiamen University, Xiamen, China.
[2]School of Informatics, The University of Edinburgh, Edinburgh, United Kingdom.
[3]Institute of Artificial Intelligence, Xiamen University, Xiamen, China.
[4]Laboratory of AI for Electrochemistry (AI4EC), Tan Kah Kee Innovation Laboratory (IKKEM), Xiamen, China.
[5]School of Chemistry and Chemical Engineering, Shanxi University, Taiyuan, China.

*Corresponding author(s). E-mail(s): j.z.pan@ed.ac.uk; wangye@xmu.edu.cn; chengjun@xmu.edu.cn;
†These authors contributed equally to this work.

Table of Contents for Supporting InformationSupplementary Information:

Section S1. Methods

Literature Collection and Parsing

Full-Text Data Extraction

Data Cleaning and Standardization Process

Data Transformation and Knowledge Storage

Relay Catalysis Pathway Search from Knowledge Graph

Relay Catalysis Pathways Scoring, Ranking, and Filtering

LLM-Assisted Presentation of Relay Catalysis Pathway

Section S2. Data Extraction Performance Evaluation

Section S3. Update Process and Entity Disambiguation in Cat-KG

Section S4. Comparison of LLM Pathway Generation with and without Knowledge Graph Support

Section S5. Relay Catalysis Pathways Identified and Recommended for Various Compounds

Section S5. Supplementary Figures

Figure S48. Chem-Brain article management interface.

Figure S49. Prompts for Automated Extraction of Catalytic Reaction Overviews.

Figure S50. Catalytic Reaction Overview in JSON Format.

Figure S51. Prompts for Automated Extraction of Catalytic Reaction Details.

Figure S52. Catalytic Reaction Details in JSON Format.

Figure S53. Cypher Query for Pathway Search in Cat-KG.

Figure S54. Text-Based Output of Pathway Search Results in Cat-KG.

Figure S55. Visualization of Pathway Search Results in Neo4j.

Figure S56. Top-Ranked Candidate Pathways for Crotonaldehyde Synthesis.

Figure S57. Catalytic Pathway Query Interface.

Figure S58. Comparison between the reported and KG-based recommended relay catalysis pathways for ethylene synthesis.

Figure S59. Comparison between the reported and KG-based recommended relay catalysis pathways for ethanol synthesis (via DME and MA).

Figure S60. Comparison between the reported and KG-based recommended relay catalysis pathways for ethanol synthesis (via methanol and acetic acid).

Figure S61. Comparison between the reported and KG-based recommended relay catalysis pathways for 2,5-furandicarboxylic acid (FDCA) synthesis.

Supplementary References

Section S1. Methods

Literature Collection and Parsing

To build a strong dataset for heterogeneous catalysis, thermal catalysis and relay catalysis studies, we used a targeted approach in our literature search. We chose 13 specific keywords to guide our search and cover a wide range of topics in catalysis. These keywords were: ‘catalysis’, ‘catalysts’ ‘heterogeneous catalysis’, ‘nanocatalysis’, ‘tandem catalysis’, ‘relay catalysis’ ,‘solid catalysts’, ‘industrial catalysis’, ‘catalytic & conversion’, ‘catalytic & oxidation’, ‘catalytic & hydrogen’, ‘catalytic & reduction’ and ‘Fischer-Tropsch synthesis’. We focused on well-known chemistry journals and publishers to ensure that we included high-quality, peer-reviewed publications with detailed information on catalytic reactions. We only searched for documents published from January 1, 2000, onwards to include the most recent research.

We collected 15,881 publications in XML, MHTML or HTML format and stored them in the data management system, Chem-Brain (see Figure S48). This webpage format included all important information, except supplementary information, making it easier to parse and process the data later.

All full-text articles used to construct Cat-KG were obtained through authorized sources. Open-access articles were collected from journals such as *Nature Communications*, *Scientific Reports*, *Chemical Science*, *JACS Au*, and *Science Advances*, which permit text and data mining under open licenses. For subscription-based content, access was provided either via Elsevier’s licensed API or through institutional library access authorized for academic use. All data were used for non-commercial scientific research purposes.

We developed a comprehensive workflow for processing literature and used a specific matching method to extract and store key information from selected publications, such as authors, title, citations, journal name, DOI, abstract, paragraphs, figure captions, scheme captions, and table captions (Figure 3b). First, we created a Python script to parse the literature and extract relevant data. This information is systematically stored in the literature section of the Chem-Brain system. Using Python-based extraction methods, we successfully parsed and collected key information from 15,881 publications from different journals.

Full-Text Data Extraction

To extract high-quality and complete catalytic reaction data from literature, we developed a systematic workflow, as shown in Figure 3b. This workflow consists of two main stages: the extraction of a Reactions Overview, followed by the Reaction Details extraction. This phased approach allows us to first identify the primary catalytic reactions within the literature and then delve deeper into extracting comprehensive data related to these reactions, ensuring the completeness and accuracy of the data.

In the Reactions Overview extraction stage, we utilized one of the powerful LLMs, Gemini 1.5 Flash, which excels in processing long texts and executing complex instructions. We designed specialized TypeScript prompts (as detailed in Figure S49) that include criteria for determining the relevance of the publication, identifying

valid reactions, and emphasizing the importance of comprehensive data extraction. These prompts generate structured JSON formats (Figure S50), facilitating automatic parsing and storage. The use of TypeScript offers advantages such as precise data definition, early error detection through strict type-checking, and modular design, ensuring clarity and consistency in the extracted data. At this stage, the LLM identifies the main catalytic reactions in the literature, extracting key information such as reactants, products, catalysts, and the type of catalytic reaction, while filtering out non-thermal catalysis reactions. This method effectively screens valuable reaction data, laying the foundation for the detailed extraction phase that follows.

In the Reaction Details extraction stage, based on the results from the overview extraction, we used Gemini 1.5 Flash to further enrich each identified reaction. Detailed information, including specifics on reactants, catalysts, reaction conditions, solvents, and products, encompassing 29 key reaction details, especially the rich catalyst information often lacking in other databases [1–4], was automatically extracted via an API. The prompts were designed to ensure comprehensive extraction, instructing the model to fill in missing data and validate correctness, thus ensuring the completeness and accuracy of the collected data (Figure S51). TypeScript ensured that all required fields were correctly populated, providing a clear template for data extraction. By defining complex interfaces and nested structures, TypeScript enabled detailed and organized representation of the data (Figure S52), reducing errors and improving the accuracy of the extraction process.

It is important to note that all interactions were conducted in the command line within a Jupyter Notebook environment, with no history of previous Q&A sessions retained. This approach ensures that the context from different articles or paragraphs does not influence the generated responses, maintaining the integrity and specificity of the extracted data for each text and reaction. The extracted literature information, reaction overview, and detailed reaction data are automatically stored in JSON files. Once extraction is complete, these JSON files are parsed and stored within the Chem-Brain platform, ensuring organized and accessible data for future research. Catalytic reaction data is obtained through the workflow outlined in Figure 3 and subsequently stored in the Chem-Brain system.

Data Cleaning and Standardization Process

To further improve data quality, we implemented a comprehensive data cleaning and verification strategy. To overcome the time constraints associated with manual verification, we developed and applied a series of automated rules aimed at refining the raw catalytic reaction data.

To address the issue of non-standard JSON structure and key names in LLM responses, we first used Python scripts to automatically parse the responses and convert them into a standardized JSON data structure with consistent key-value pairs. In the second step, we used Python scripts to clean each reaction or key-value pair. This included unifying compound names, eliminating duplicate reactions, and normalizing values and units for temperature, pressure, conversion, yield, selectivity, and reaction time.

As part of the cleaning process, we normalized values and units for temperature, pressure, conversion, yield, selectivity, and reaction time. We also removed meaningless attribute values, such as entries with missing unit information for temperature and pressure. To ensure consistency across the dataset, we implemented an automatic unit normalization mechanism for all extractable numerical values. Specifically, we parsed each key-value pair to separate the numeric value from the unit string, then converted temperature values to Kelvin (K) and pressure values to bar. This standardization step improves the comparability and uniformity of reaction conditions across heterogeneous data sources. Moreover, by storing numerical values and unit metadata in a structured form, our system supports future integration of automatic unit conversion functions, which will enhance interoperability with diverse user inputs and external datasets.

We further enhanced the dataset's integrity through a compound standardization process manually, utilizing the Pubchem and ChEBI databases, which are commonly used knowledge bases for compound alignment [5]:

- We identified all substance names from the dataset.
- We matched these names against the Pubchem database to find the most appropriate Compound Identifier (CID).
- Using the identified CIDs, we retrieved detailed compound information, including ChEBI ID, CAS numbers, and synonyms. This involved calculating text similarity scores to ensure the selection of the most appropriate matches.
- We conducted manual verification to ensure accuracy.

This standardization process resolved issues related to duplication and inaccuracies in the dataset, leading to the creation of a unified and accurate compound naming table. The process and methodologies employed significantly enhanced the quality of the catalytic reaction data, making it suitable for rigorous scientific analysis and applications.

Data Transformation and Knowledge Storage

After the data cleaning and standardization process, the resulting JSON files contained structured key-value data. This data formed the basis for constructing the knowledge graph (KG). To effectively visualize this data as a KG, we chose to use the Neo4j database, a high-performance graph database well-suited for storing and querying complex network relationships[6].

First, we parsed the collected JSON files to extract the key nodes and relationships. Nodes represent entities in the KG, such as reactants, products, and reactions, while relationships represent the connections between these entities, such as 'is_reactant_of' and 'has_product'. To accomplish this, we wrote scripts to traverse the data structure of the JSON files and convert each entity and relationship into Neo4j's node and relationship format.

Next, we used Neo4j's import tools to load the parsed data into the Neo4j database. During the import process, we utilized Cypher [6] to create nodes and relationships, and we added the corresponding properties to each node and relationship. By following these steps, we successfully transformed the structured data from the JSON files into a KG in the Neo4j database. This KG supports visualization, querying, and further analysis.

Relay Catalysis Pathway Search from Knowledge Graph

To conduct relay catalysis pathway searches within the Cat-KG, we used Python scripts together with the Neo4j database. The search begins by defining the target compound and specifying the desired conditions for each reaction step. Users can set parameters such as required reactants, excluded substances, reaction heterogeneity, and limitations on intermediate compounds in the relay catalysis pathway (Figure S57). These parameters are then used to dynamically generate Cypher queries through designed Python functions tailored to the user's specifications.

The Cypher queries are constructed by first identifying the target compound within Cat-KG and then matching it to relevant reactions. The process iterates through each step of the pathway, applying the user-defined conditions to ensure that the search results align with the specified requirements. For instance, when searching for pathways leading to Crotonaldehyde, the script generates a query that includes CO and H_2 as required reactants and filters for heterogeneous reactions (Figure S53). The generated queries are then executed against the Neo4j database, retrieving pathways that meet the specified criteria.

To refine the search results, the pathways are subjected to a filtering and deduplication process. The filtering involves examining the sequence of reactions within each pathway to ensure that substances are not repeated. Duplicate pathways—those that share identical sequences of reactions—are identified and removed. This process ensures that the final results include only unique and relevant pathways, reducing redundancy and enhancing the quality of the data.

The results of the pathway search can be output in two formats: text-based and graphical. The text-based output lists the pathways, providing detailed information about each reaction step, including the reactants, products, and conditions (Figure S54). This format is particularly useful for researchers who need a clear and concise overview of potential pathways and is also conducive to automated analysis and processing. Additionally, users have the option to visualize the pathways using the Neo4j app. The generated Cypher queries can be copied to the Neo4j interface, where the pathways are displayed as nodes and relationships, offering an intuitive visual representation of the catalytic processes (Figure S55). This graphical format is advantageous for exploring complex interactions and understanding the broader context of the pathways.

Relay Catalysis Pathways Scoring, Ranking, and Filtering

For all candidate pathways obtained from Cat-KG (currently only considering thermocatalytic relay paths), we designed scoring rules to evaluate and rank the effectiveness and value of the reaction paths. Our scoring method considers the reliability and effectiveness of single-step reactions in the pathway, as shown in part A. It also considers the compatibility of reaction conditions and catalysts in adjacent steps, as shown in part B. Additionally, the reaction phase and atom economy of the entire pathway are considered, as shown in part C. The calculation methods for each part and the steps for calculating the final score are as follows:

A: Individual Reaction Scoring

This section evaluates the effectiveness of each reaction in the pathway, considering journal and literature information, key data completeness, and reaction performance. Each reaction is given a score based on the following criteria:

- **Journal Impact Factor:** Higher impact factors get higher scores, from 0 to 100 points. The impact factor is scaled linearly, with the maximum factor corresponding to 100 points. This measures the importance of the journal where the article is published.
- **Publication Date:** More recent publications receive higher scores, from 0 to 100 points. The score is based on the number of days from the publication date to a fixed end date (e.g., 2024-07-30). Publications before January 1, 2000, score 0 points, and those on or after July 30, 2024, score 100 points. This ensures recent research is given appropriate weight.
- **Citations:** Higher citation counts receive higher scores, up to 100 points. Each citation equals 1 point, with a maximum of 100 points. This measures the impact and recognition of the research within the scientific community.
- **Catalyst Information Completeness:** Details such as loading, support, preparation method, promoters, metal oxidation states, BET surface area, turnover frequency, shape, pore size, and pore volume are scored. Each complete piece of information adds 20 points, up to a maximum of 200 points. Comprehensive catalyst information aids in analysis, evaluation, and experimental reproducibility.
- **Temperature Information Completeness:** If the reaction temperature is specified, 100 points are awarded. Clear temperature data is crucial for reaction reproducibility and pathway evaluation and design.
- **Gas and Pressure Information Completeness:** If both the gas name and pressure are specified, 100 points are awarded. Complete gas and pressure information is essential for reaction reproducibility and pathway evaluation and design.
- **Reaction Time Completeness:** If the reaction time is specified, 50 points are awarded. Documenting the reaction duration is important for understanding reaction kinetics and pathway design.
- **Reaction Performance:** Evaluates product yield (a), selectivity (b), and reactant conversion (c). The score is calculated using the formula:

$$S_{performance} = 100 \times \max(b \times c, a) \tag{1}$$

  This scoring strategy prioritizes reactions with high yield, selectivity, and conversion rates, aiming for high target product output.

B: Scoring Between Consecutive Reactions

This section evaluates the similarity and compatibility of conditions between consecutive reaction steps. It focuses on the similarity of the main catalyst, support, promoter, temperature, gas, pressure, and solvent. Each pair of consecutive reactions is given a score based on the following criteria:

- **Catalyst Name Similarity:** If the catalyst names are the same, 200 points are awarded. The comparison is case-insensitive to ensure uniform scoring.
- **Support Material Similarity:** If the support materials are the same, 100 points are awarded. Using the same support aids in the design and synthesis of catalysts in the relay pathway and avoids negative interactions between different supports.
- **Promoters Similarity:** If the promoters are the same, 100 points are awarded. Similar promoters prevent negative interactions between different steps and facilitate the use of reaction conditions from the literature.
- **Temperature Proximity:** The score is based on how close the temperatures of two consecutive reactions are. The formula used is:

$$S_{temperature} = \frac{9000}{|T1 - T2| + 30} \tag{2}$$

  This favors reactions with similar temperatures, increasing the likelihood of achieving good performance under the same conditions and simplifying the optimization process.
- **Gas Name Similarity:** If any gas names are the same, 50 points are awarded. This criterion recognizes the presence of similar gases.
- **Gas Pressure Similarity:** Additional points are awarded based on the similarity of pressures for common gases. If the gas names are the same and both pressures are valid, the relative difference in pressures is calculated and scored as follows:

$$S_{pressure} = 200 \times \left(1 - \frac{|P1 - P2|}{P1 + P2}\right) \tag{3}$$

  Using similar gas environments with close pressures aids in the success of relay catalytic experiments and makes implementation easier.
- **Solvent Similarity:** If any solvent names are the same, 100 points are awarded. The comparison is case-insensitive. Using similar solvents ensures high reactivity under similar conditions if a liquid phase is involved.

C: Overall Reaction pathway Scoring

This section evaluates whether the reaction types are consistently heterogeneous, the availability of starting materials, and the complexity of the reaction system.

- **Reaction Type Consistency:** If all reactions have the same `isHeterogeneous` value, 100 points are awarded. This ensures consistency in reaction types is rewarded.
- **Initial Reactants Simplicity:** The reactants of the first reaction are checked against a list of simple and readily available compounds. If all reactants are on the list, 200 points are awarded. This criterion favors pathways that use easily accessible starting materials.
- **Additives Simplicity:** The number of unique chemicals (reactants, products, solvents, gases) is counted. Fewer chemicals receive higher scores, up to 100 points. The scoring formula is:

$$S_{additives} = 10 \times (15 - n) \tag{4}$$

This favors simpler reactions with fewer additives, promoting shorter pathways to achieve the target product.

The effectiveness of each relay catalysis pathway is quantified by calculating a **Relay Catalysis Score**. This score is obtained by summing the individual reaction scores, the scores for connections between reactions, and the overall pathway scores. The formula used to calculate the total score S for each pathway is as follows:

$$S = \sum SA_i + \sum SB_j + \sum C \tag{5}$$

where SA_i represents the scores for individual reactions, SB_j represents the scores for reaction connections, and C represents the overall pathway scores.

All pathway scores are computed using the above method, with a Python script automating the entire process. The script calculates a score for each relay catalysis pathway, then filters and ranks the pathways based on these scores. The results are stored in a text file (Figure S56), which includes the pathway scores, rankings, and detailed reaction information. This information comprises the reactants, products, catalysts, temperatures, literature titles, and reaction IDs for each step of the pathway. The literature titles and reaction IDs are specifically included to allow tracing back to the original sources in Chem-Brain, providing access to detailed raw data and literature information for further analysis. The highest-scoring pathways are listed at the top of the text file, indicating their higher value, while lower-scoring pathways are placed at the bottom, reflecting their lesser value. This approach enables researchers to quickly identify and focus on the most promising relay catalysis pathways from hundreds to thousands of candidates.

LLM-Assisted Presentation of Relay Catalysis Pathway

After the relay catalysis pathways are scored and ranked, researchers can select pathways of interest based on the text-formatted results, which include information on reactants, intermediates, reaction temperatures, catalysts, and relevant literature titles (Figure S56). By using the pathway ID corresponding to their selection, researchers can trace and extract detailed JSON-formatted information for each step of the pathway.

However, this JSON format, while detailed, is not user-friendly and can be challenging to interpret (Figure S52). To address this issue, we adopted two complementary strategies. The first strategy is to present readable reaction pathway information directly on our query interface (Figure S57), where the results are organized in a user-friendly format for easy inspection and interpretation. The second strategy leverages the natural language generation capabilities of a LLM to convert structured pathway data into chemical equations and human-readable descriptions. Specifically, we designed a tool named *Relay Catalysis Analyzer* within the GPTs (Figures S3, S7) using the functionalities provided by OpenAI's GPTs. This tool can be easily found and accessed through the GPTs store (https://chatgpt.com/g/g-8XM17gFuc-relay-catalysis-analyzer).

Researchers can provide the structured JSON pathway information to the *Relay Catalysis Analyzer* without the need for additional input or explanation. The tool automatically and efficiently formats this information into a readable structure, including clear and concise reaction pathways, key conditions, compatibility analyses of

reactions and conditions, and highlighted analyses of each reaction step Figure 4c, Figure S3, S7). This method is highly flexible, allowing the customization of different tools within GPTs to meet various research needs, making our approach adaptable to different tasks and requirements.

Section S2. Data Extraction Performance Evaluation

To assess the effectiveness of Gemini in extracting full-text data and evaluate the data quality, we established specific evaluation standards. First, a representative set of reactions was selected as evaluation data. Specifically, we used reactions from the synthesis pathways of ten compounds—lower olefins, ethylene, ethylene glycol, oxalic acid, propylene glycol, crotonaldehyde, 1,3-butadiene, 1,4-butanediol, cis-2-butene, and 2,5-furandicarboxylic acid—identified in Section S5 of the Supporting Information: *Relay Catalysis Pathways Identified and Recommended for Various Compounds*. These reactions were chosen to ensure coverage of major reaction types. After removing duplicates, a total of 44 reactions were included in the evaluation. The data were then assessed using previously reported methods [7, 8].

During manual validation, each synthesis parameter extracted by Gemini was verified and annotated. Correctly extracted parameters were classified as true positives (TP, parameters correctly identified by Gemini), incorrect extractions were classified as false positives (FP, values assigned to the wrong category or irrelevant data extracted), and missed parameters were classified as false negatives (FN, information that Gemini failed to extract). Precision was calculated as the proportion of correct records out of all extracted records, while recall measured the proportion of correctly extracted records out of all existing records in the literature. The F1 score, representing the harmonic mean of precision and recall, was used to evaluate performance on this potentially imbalanced dataset [7].

$$\text{Precision} = \frac{\text{TP}}{\text{TP} + \text{FP}} \tag{6}$$

$$\text{Recall} = \frac{\text{TP}}{\text{TP} + \text{FN}} \tag{7}$$

$$\text{F1} = 2 \times \frac{\text{Precision} \times \text{Recall}}{\text{Precision} + \text{Recall}} \tag{8}$$

We calculated precision, recall, and F1 scores for the 44 reactions. The accumulative metrics, as a function of the number of reactions evaluated, are shown in Figure S1a and b. Precision and recall values, along with their standard errors, are displayed next to their legends. The curves demonstrate that the precision and recall results are highly stable, with fluctuations of less than 1% within the evaluated samples. These results suggest that the findings can be extrapolated to the entire dataset.

The calculated mean values for the 44 reactions are as follows: Mean Precision: 91.49% (SE: 1.07%), Mean Recall: 91.18% (SE: 1.04%), and Mean F1-Score: 0.9113 (SE: 0.0086). The fact that both precision and recall exceed 90%, and the F1 score exceeds 0.9, indicates that Gemini's automated extraction process for catalytic data yields high-quality results. Furthermore, the low standard errors confirm the high stability and robustness of the extraction method. These findings give confidence that the evaluation can be extended to the entire dataset, demonstrating the effectiveness of the Gemini-based data extraction pipeline.

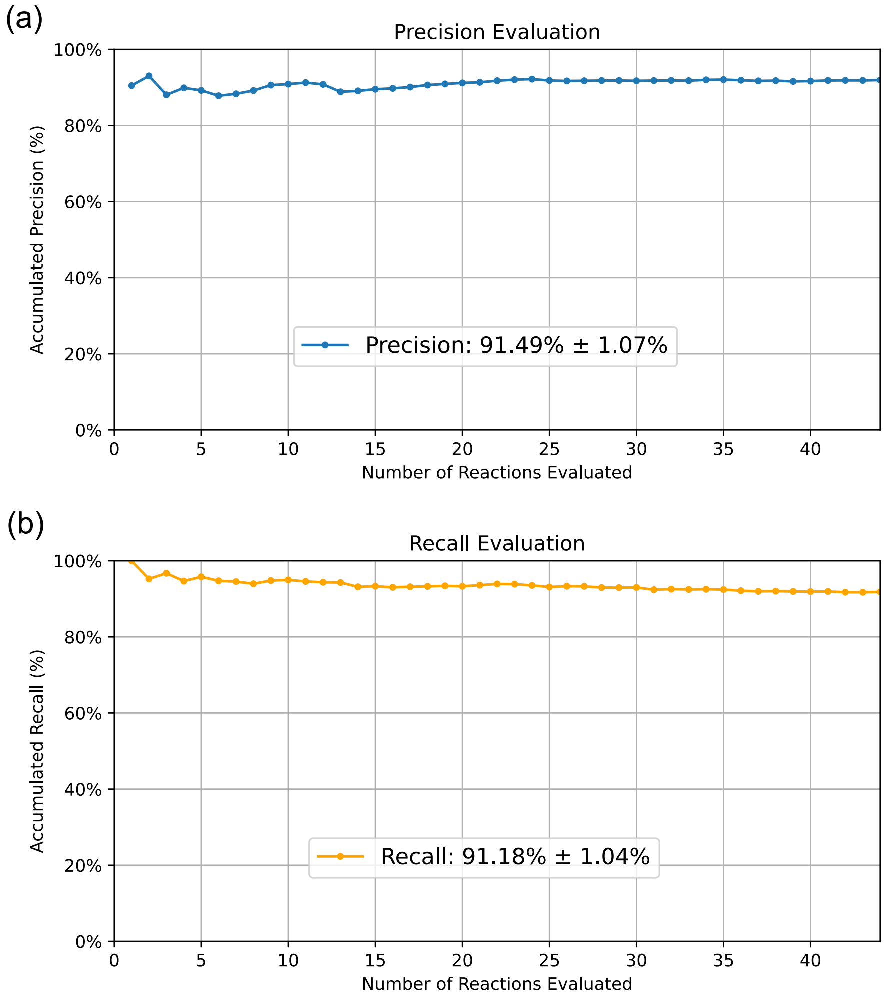

Figure S1. Evaluations of precision and recall for the extracted catalytic reaction data. Plots (a) and (b) show the accumulative precision and recall, respectively.

Section S3.Update Process and Entity Disambiguation in Cat-KG

As real-world knowledge continuously evolves, new entities and relations emerge over time, requiring KGs to be dynamically updated in order to remain current and relevant[9–12]. A prominent example of a dynamically maintained KG is Wikidata, which integrates both human curation and automated bot-based batch updates to achieve large-scale and high-frequency knowledge maintenance. In recent years, various mechanisms have been proposed to enable KGs to incorporate new knowledge continuously without the need for complete reconstruction[10, 11]. Among them, two dominant approaches are incremental updates and real-time /streaming updates. Incremental updating involves periodically or irregularly integrating newly added or modified knowledge entries into the existing graph structure, thereby avoiding full-scale reloading. This method emphasizes updating only the affected subgraphs to reduce computational overhead. In contrast, streaming updates are designed to achieve higher real-time responsiveness. In this model, knowledge sources are treated as continuous data streams, and the KG system responds to incoming events by immediately triggering update operations. This can be implemented, for instance, by using message queue systems such as Kafka to monitor source data changes and execute mini-batch insertions in near real-time. These strategies provide a technical foundation for building KGs that are capable of evolving in alignment with the dynamic nature of their underlying domains.

Given that Cat-KG features large-scale data volume, a well-defined schema, and a non-streaming, batch-like pattern of knowledge growth, a full reconstruction strategy for updating the graph would be both time-consuming and inefficient, and thus unsuitable for supporting continuous evolution and rapid iteration. In the field of catalysis, new knowledge is predominantly derived from periodically published scientific literature, which results in knowledge being generated in discrete batches rather than through high-frequency data streams. Therefore, a scheduled, batch-based update approach is more practical and efficient for this context. To meet this need, Cat-KG adopts a script-driven incremental update mechanism. Specifically, each time the system completes automatic information extraction from approximately 3,000 research articles (see Section S1. Methods), an update pipeline is triggered. The extracted content is first converted into a JSON file conforming to the predefined schema, and then processed using a series of Python scripts before being imported into the Neo4j graph database via Cypher queries. The processing workflow includes normalization of JSON field names and structure, unit standardization, compound name alignment and unification, filtering of invalid or incomplete fields and reactions, and comprehensive data cleaning and standardization (detailed in Section S1. Methods). From the cleaned JSON, entities and relationships are extracted, index tables are generated, and the data is batch inserted into the existing KG. This fully automated pipeline—from raw literature to graph updates—significantly enhances processing efficiency and data consistency while minimizing human error. Particularly in the catalytic domain, where knowledge emerges at a regular but moderate pace and exhibits a highly structured format, this update mechanism offers a technically feasible, cost-effective, and scalable

solution. It has thus become the core infrastructure supporting the dynamic evolution of Cat-KG.

During each incremental update of the KG, Cat-KG performs essential data validation and conflict detection through automated scripts to ensure the structural integrity and semantic consistency of the graph. Specifically, when the system detects that a newly extracted entity may already exist in the graph, it does not directly create a new node. Instead, it checks whether the entity has been previously recorded by comparing its unique identifier, such as the PubChem CID. If the entity already exists, the system updates its attributes or adds new relationships; if not, it creates a new node. This approach reflects the core principle of conflict resolution in KG updates—ensuring entity uniqueness to avoid redundancy and inconsistency[5, 12, 13]. Embedding entity validation in the automated workflow is crucial for filtering out invalid or duplicate data. This includes checking data types, standardizing field formats, and verifying entity uniqueness. Among these, entity disambiguation is particularly important[12–14]. It refers to the process of linking ambiguous mentions in text or structured data to a single, well-defined entity in the knowledge base. This step is critical not only for maintaining uniqueness within the graph, but also for enabling accurate reasoning and cross-database integration, making it a central part of Cat-KG's update framework.

In the chemical and catalysis domains, inconsistent naming is a major challenge for entity disambiguation[5]. Even for simple compounds like hydrogen, different literature sources may use names such as "Hydrogen," "H_2," "hydrogen gas," or "molecular hydrogen." Without normalization, these variations can lead to duplicate nodes and structural confusion in the graph. To address this, Cat-KG adopts a disambiguation strategy based on authoritative chemical databases, which is a commonly used approach for compound entity alignment [5]. All compound entities in the graph, including reactants, products, gases, and solvents, are collected and manually aligned with PubChem or ChEBI to obtain a unique identifier (CID or ChEBI ID). This identifier is then used as the primary key for each compound node to ensure that the same compound appears only once in the graph. For example, all names referring to CID 783 (hydrogen) are unified into a single node, and Neo4j enforces uniqueness on the CID field to prevent duplication. For compounds not recognized by these databases, such as those with code names like "Compound-21" or abbreviations like "BTB," the original name is used as a unique identifier. This strategy allows Cat-KG to standardize chemical entities without sacrificing data coverage, ensuring reliable and consistent updates to the KG.

Cat-KG establishes an efficient and reliable mechanism for dynamic updates and entity disambiguation within the KG. At the system level, a structured, script-based incremental update pipeline enables Cat-KG to continuously integrate newly extracted catalytic knowledge from scientific literature without the high cost of full reconstruction. This ensures that the graph evolves dynamically and remains up-to-date. The update process includes data standardization, field normalization, and structural validation, which together guarantee the consistency and stability of each update cycle. At the entity level, Cat-KG applies a rule-based disambiguation strategy using authoritative chemical databases such as PubChem and ChEBI. By mapping compound names to unique identifiers (e.g., CID), the system avoids duplication and semantic conflict

caused by synonyms. This approach improves the accuracy and uniqueness of chemical entities, while also providing a consistent anchor for linking external resources and expanding graph attributes. Compared to complex machine learning models, Cat-KG's method is more transparent, controllable, and traceable—qualities that are especially important in scientific applications where consistency and interpretability are critical. Overall, this design effectively addresses key challenges in integrating dynamic knowledge in the catalysis domain, enabling high-quality fusion of new and existing data from diverse sources, and laying a solid foundation for building a living, evolving KG.

Section S4.Comparison of LLM Pathway Generation with and without Knowledge Graph Support

In this study, we compared the ability of a large language model (LLM) to generate reaction pathways with and without the support of reliable catalytic reaction knowledge from the catalysis knowledge graph (Cat-KG). The experiments aimed to assess the LLM's capacity to recommend accurate and innovative reaction pathways solely through prompts versus when provided with structured reaction data.

When prompted without any background knowledge, the LLM was able to generate reasonable pathways that adhered to well-established industrial standards, such as a three-step relay catalysis process for ethanol synthesis. We designed a prompt to query GPT-4, asking for a three-step relay catalysis pathway for ethanol[1], as shown in Figure S2. The generated pathway involved: (1) the formation of methanol from hydrogen and carbon monoxide using $Cu/ZnO/Al_2O_3$ as a catalyst, (2) conversion of methanol to dimethyl ether (DME) using γ-Al_2O_3, and (3) subsequent conversion of DME to ethanol using Cu/Al_2O_3, all under typical industrial conditions of temperature and pressure. In contrast, we conducted a test using the pathway search and recommendation process developed in this study. We first queried Cat-KG and obtained a ranked recommendation for an ethanol synthesis pathway. To further describe the pathway, we utilized structured reaction information from reliable literature and employed an application developed and deployed within GPTs, called *Relay Catalysis Analyzer*[2], to process catalytic data and generate pathway descriptions. The results[3] obtained through this method are shown in Figure S3. The results demonstrate that the reaction conditions in each step of the relay catalysis pathway recommended by the method in this paper are well-matched, such as a consistent temperature of 523 K, and that each step exhibits high selectivity, providing clear and reliable reaction information.

Additionally, we conducted pathway recommendation tests for crotonaldehyde. First, we designed a prompt to query GPT-4, asking for a three-step relay catalysis pathway for crotonaldehyde synthesis starting from syngas without access to domain-specific knowledge. We performed three parallel tests[4][5][6], as shown in Figures S4, S5 and S6. The same pathway search and recommendation process developed in this study was also applied[7], as shown in Figure S7.

Analyzing the recommendations for ethanol and crotonaldehyde synthesis under both conditions— with and without reliable catalytic reaction knowledge from Cat-KG—revealed clear differences in the quality of the pathways. When the LLM generated pathways without the support of the KG, the results tended to rely heavily on well-established industrial processes, offering little innovation or novelty. These pathways often lacked references to literature or data sources, making it difficult for non-experts to assess their reliability. Additionally, in some cases, the LLM failed to follow the provided instructions precisely, such as recommending two-step relay

[1] https://chatgpt.com/share/f7aed650-2cbe-4c30-8a3b-d28c7806bef4
[2] https://chatgpt.com/g/g-8XM17gFuc-relay-catalysis-analyzer
[3] https://chatgpt.com/share/67067586-3060-8004-895e-73dcfcb6d7d0
[4] https://chatgpt.com/share/66f64b6b-0d78-8004-9b31-b6474bccacd5
[5] https://chatgpt.com/share/66f64c2b-9968-8004-8f84-9ac3dae24320
[6] https://chatgpt.com/share/66f64bbd-67c0-8004-951f-ff9198a956a8
[7] https://chatgpt.com/share/66f65125-0c38-8004-9cf5-ec49f56c8a9e

Please generate a plausible three-step relay catalytic reaction pathway to synthesize ethanol starting from hydrogen (H2) and carbon monoxide (CO). The reaction should involve multiple catalytic steps, and for each step, please provide the temperature and pressure conditions. The temperature and pressure values should be as consistent as possible across all steps. If you are uncertain, please reply with 'I do not know'.

Here is a plausible three-step relay catalytic reaction pathway to synthesize ethanol (C_2H_5OH) starting from hydrogen (H_2) and carbon monoxide (CO). The reaction pathway involves multiple catalytic steps:

Step 1: Formation of Methanol (CH_3OH)

- **Reactants:** $H_2 + CO \rightarrow CH_3OH$
- **Catalyst:** $Cu/ZnO/Al_2O_3$
- **Temperature:** 240-260°C
- **Pressure:** 50-100 bar

Step 2: Methanol to Dimethyl Ether (DME)

- **Reactants:** $2\ CH_3OH \rightarrow CH_3OCH_3 + H_2O$
- **Catalyst:** γ-Al_2O_3
- **Temperature:** 250-300°C
- **Pressure:** 50-100 bar

Step 3: DME to Ethanol (C_2H_5OH)

- **Reactants:** $CH_3OCH_3 + H_2 \rightarrow C_2H_5OH$
- **Catalyst:** Cu/Al_2O_3
- **Temperature:** 220-250°C
- **Pressure:** 50-100 bar

This pathway uses consistent temperature and pressure conditions across all steps to synthesize ethanol from hydrogen and carbon monoxide, with slight variations to optimize each catalytic step.

Figure S2. Ethanol Synthesis Pathway Generated from GPT-4 without Domain-Specific Knowledge. A three-step relay catalysis pathway for ethanol synthesis generated by GPT-4 without access to domain-specific knowledge.

```
    }
  ]
}
--------------------------------------------------
```

1. Catalytic Pathway Description

```markdown
CO + H₂ --------> CH₃OH --------> Methyl Acetate --------> Ethanol
   Cat.-[HzIn₂O₃-x(OH)y]   Cat.-[H-ZSM-5]     Cat.-[Cu/SiO₂]
   Temp.-[523.0 K]         Temp.-[523.0 K]    Temp.-[523.0 K]
```

2. Reaction Compatibility Analysis

The temperatures across all steps are perfectly aligned at 523.0 K, ensuring an optimal reaction flow with minimal need for thermal adjustment, which promotes energy efficiency throughout the catalytic sequence. Hydrogen is utilized both in the initial CO hydrogenation (step 1) and again in the final methyl acetate hydrogenation (step 3), offering the benefit of reactant reuse and enhancing economic efficiency. Moreover, the use of relatively common catalysts like H-ZSM-5 and Cu/SiO_2, both of which are well-established in industrial applications, supports cost-effective implementation, especially given their widespread availability and high catalytic performance. The selective production of methanol (49.23% selectivity in step 1) and the excellent conversion rates of acetaldehyde (90.5% in step 2) and methyl acetate (95.4% in step 3) indicate a streamlined pathway with relatively high yields. Importantly, Cu/SiO_2 as the final catalyst in the hydrogenation step benefits from a high surface area (569 m^2/g), which can improve contact between reactants and active sites, further enhancing the reaction efficiency.

3. Reaction Highlights

- **Step 2:** High acetaldehyde conversion (90.5%) during methanol-to-olefins conversion on H-ZSM-5.
- **Step 3:** Outstanding performance with 95.4% methyl acetate conversion and 90.4% ethanol yield, coupled with 95.8% selectivity toward ethanol on the Cu/SiO_2 catalyst.

Figure S3. Ethanol Synthesis Pathway Generated with Domain-Specific Knowledge from Cat-KG. A three-step relay catalysis pathway for ethanol synthesis generated using structured reaction information from Cat-KG and processed by the *Relay Catalysis Analyzer*.

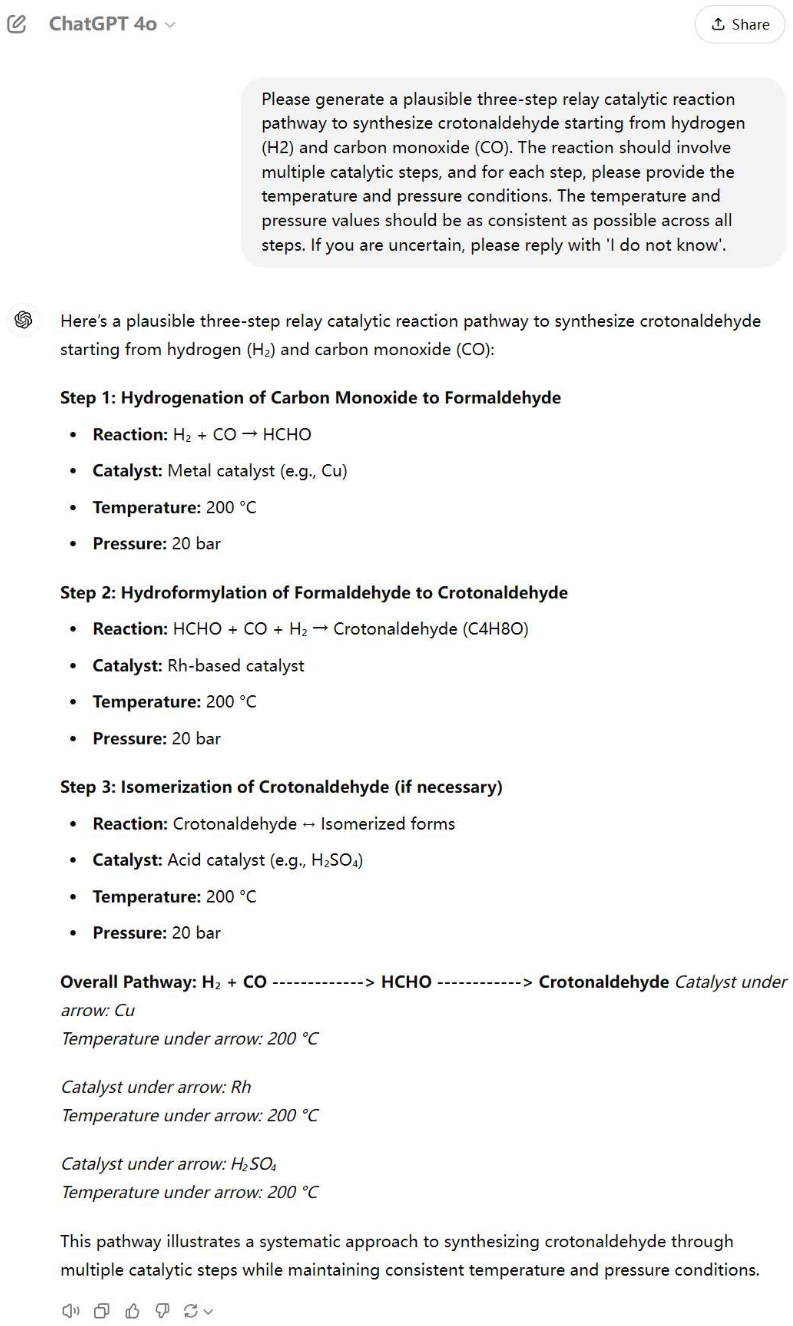

ChatGPT 4o

Share

Please generate a plausible three-step relay catalytic reaction pathway to synthesize crotonaldehyde starting from hydrogen (H2) and carbon monoxide (CO). The reaction should involve multiple catalytic steps, and for each step, please provide the temperature and pressure conditions. The temperature and pressure values should be as consistent as possible across all steps. If you are uncertain, please reply with 'I do not know'.

Here's a plausible three-step relay catalytic reaction pathway to synthesize crotonaldehyde starting from hydrogen (H_2) and carbon monoxide (CO):

Step 1: Hydrogenation of Carbon Monoxide to Formaldehyde

- **Reaction:** H_2 + CO → HCHO
- **Catalyst:** Metal catalyst (e.g., Cu)
- **Temperature:** 200 °C
- **Pressure:** 20 bar

Step 2: Hydroformylation of Formaldehyde to Crotonaldehyde

- **Reaction:** HCHO + CO + H_2 → Crotonaldehyde (C4H8O)
- **Catalyst:** Rh-based catalyst
- **Temperature:** 200 °C
- **Pressure:** 20 bar

Step 3: Isomerization of Crotonaldehyde (if necessary)

- **Reaction:** Crotonaldehyde ↔ Isomerized forms
- **Catalyst:** Acid catalyst (e.g., H_2SO_4)
- **Temperature:** 200 °C
- **Pressure:** 20 bar

Overall Pathway: H_2 + CO -------------> HCHO ------------> Crotonaldehyde *Catalyst under arrow: Cu*
Temperature under arrow: 200 °C

Catalyst under arrow: Rh
Temperature under arrow: 200 °C

Catalyst under arrow: H_2SO_4
Temperature under arrow: 200 °C

This pathway illustrates a systematic approach to synthesizing crotonaldehyde through multiple catalytic steps while maintaining consistent temperature and pressure conditions.

Figure S4. Crotonaldehyde Synthesis Pathway Generated from GPT-4 without Domain-Specific Knowledge. A three-step relay catalysis pathway for crotonaldehyde synthesis generated by GPT-4 without access to domain-specific knowledge.

ChatGPT 4o

Share

Please generate a plausible three-step relay catalytic reaction pathway to synthesize crotonaldehyde starting from hydrogen (H2) and carbon monoxide (CO). The reaction should involve multiple catalytic steps, and for each step, please provide the temperature and pressure conditions. The temperature and pressure values should be as consistent as possible across all steps. If you are uncertain, please reply with 'I do not know'.

Here's a plausible three-step relay catalytic reaction pathway to synthesize crotonaldehyde from hydrogen (H_2) and carbon monoxide (CO):

Pathway Description

Step 1: Hydrogenation of Carbon Monoxide

- **Reactants:** CO + H_2
- **Catalyst:** Nickel (Ni)
- **Reaction Conditions:**
  - Temperature: 200 °C
  - Pressure: 30 bar
- **Intermediate Product 1:** Ethanol (C_2H_5OH)

Step 2: Dehydration of Ethanol

- **Reactants:** C_2H_5OH
- **Catalyst:** Acidic catalyst (e.g., sulfuric acid or alumina)
- **Reaction Conditions:**
  - Temperature: 200 °C
  - Pressure: 30 bar
- **Intermediate Product 2:** Ethylene (C_2H_4)

Step 3: Carbonylation of Ethylene

- **Reactants:** C_2H_4 + CO
- **Catalyst:** Palladium (Pd)
- **Reaction Conditions:**
  - Temperature: 200 °C
  - Pressure: 30 bar
- **Final Product:** Crotonaldehyde (C_4H_6O)

Summary

This pathway consists of a hydrogenation step to produce ethanol, followed by a dehydratior step to form ethylene, and finally a carbonylation step to synthesize crotonaldehyde, all under consistent temperature and pressure conditions.

Figure S5. Crotonaldehyde Synthesis Pathway Generated from GPT-4 without Domain-Specific Knowledge. A three-step relay catalysis pathway for crotonaldehyde synthesis generated by GPT-4 without access to domain-specific knowledge.

pathways instead of the requested three-step sequences (as shown in Figure S4). Furthermore, the LLM sometimes recommended reaction conditions or substances that were too harsh or impractical for facilitating a relay pathway, such as the use of H_2SO_4 as an additive, which would hinder the feasibility of the proposed process (as shown in Figures S4, S5, S6).

In contrast, the knowledge-driven pathway recommendation strategy, based on Cat-KG, addressed many of these issues by leveraging reliable, structured data to provide more informed and consistent recommendations. The pathways generated through this approach were supported by recent research findings, ensuring greater alignment with the latest advances in catalytic science. This data-backed method also offered clear traceability to the literature, allowing users to verify the recommendations easily and mitigating the common problem of hallucinations—where LLMs, in the absence of domain-specific data [11, 15], might generate pathways that seem valid but are chemically inaccurate or impractical for real-world application. Moreover, the Cat-KG-driven strategy demonstrated the ability to recommend novel pathways that had not yet been reported in the literature (as shown in Section S5 of the Supporting Information: *Relay Catalysis Pathways Identified and Recommended for Various Compounds*). This highlights the potential of using structured knowledge to not only avoid the pitfalls of purely prompt-based LLM approaches but also to advance catalysis research by proposing innovative, data-backed solutions.

These experimental results highlight the limitations of prompt-only LLM approaches when domain-specific data is lacking. Integrating structured KG such as Cat-KG can improve the accuracy and novelty of pathway recommendations. This observation aligns with recent evaluations of LLMs in various chemical tasks. Studies have shown that LLMs perform well in general chemical knowledge and question answering, demonstrating strong abilities in information integration, natural language interaction, and code generation—sometimes even surpassing human experts [15, 16]. However, they still face clear challenges in structural reasoning, use of domain-specific databases, and simulating chemical intuition, and are prone to hallucinations in the absence of reliable data [11, 15]. Therefore, combining LLMs with KGs is a promising strategy to leverage their complementary strengths and enhance the reliability and practicality of LLM-based systems in chemistry [11].

ChatGPT 4o

Share

Please generate a plausible three-step relay catalytic reaction pathway to synthesize crotonaldehyde starting from hydrogen (H2) and carbon monoxide (CO). The reaction should involve multiple catalytic steps, and for each step, please provide the temperature and pressure conditions. The temperature and pressure values should be as consistent as possible across all steps. If you are uncertain, please reply with 'I do not know'.

Here's a plausible three-step relay catalytic reaction pathway to synthesize crotonaldehyde from hydrogen (H_2) and carbon monoxide (CO):

Step 1: Hydrogenation of CO to form Methanol
Reactants: H_2 + CO -------------> Methanol
Catalyst under arrow: Cu/ZnO
Temperature: 250 °C
Pressure: 30 bar

Step 2: Dehydration of Methanol to form Dimethyl Ether
Reactants: Methanol -------------> Dimethyl Ether
Catalyst under arrow: Acid catalyst (e.g., H_2SO_4)
Temperature: 250 °C
Pressure: 30 bar

Step 3: Selective Hydrogenation of Dimethyl Ether to form Crotonaldehyde
Reactants: Dimethyl Ether + H_2 -------------> Crotonaldehyde
Catalyst under arrow: Pd/Al_2O_3
Temperature: 250 °C
Pressure: 30 bar

This pathway maintains consistent temperature and pressure across all steps.

Figure S6. Crotonaldehyde Synthesis Pathway Generated from GPT-4 without Domain-Specific Knowledge. A three-step relay catalysis pathway for crotonaldehyde synthesis generated by GPT-4 without access to domain-specific knowledge.

Relay catalysis analyzer

Share


```
      "reactionTime": "10.0 h",
      "gases": [
        {
          "name": "acetaldehyde",
          "pressure": "0.0709 bar",
          "cid": "177",
          "cid_name": "Acetaldehyde",
          "smiles": "CC=O"
        }
      ]
    },
    "solvents": [
      {
        "solventPH": "unknown"
      }
    ]
}
--------------------------------------------------
```


1. **Catalytic Pathway Description**

```markdown
CO + H₂ --------> Ethanol --------> Acetaldehyde --------> Crotonaldehyde
     Cat.-[CuCoMn]         Cat.-[Cu/SiO₂]          Cat.-[MoOx/γ-Al₂O₃]
     Temp.-[543.0 K]       Temp.-[553.0 K]         Temp.-[573.0 K]
```

2. **Reaction Compatibility Analysis**

The temperatures across the steps range from 543.0 K to 573.0 K, indicating that the thermal conditions are well-aligned, with only a gradual increase, which facilitates smooth transitions between each catalytic step. Notably, ethanol is a key intermediate utilized in both steps 1 and 2, enhancing the process's economic efficiency. The initial conversion of carbon monoxide is 29.7%, setting a foundation for subsequent reactions. The use of heterogeneous catalysts in each step underscores the robustness of the pathway, with copper-cobalt-manganese exhibiting notable properties in the first step and the copper/silica system achieving an impressive 99% conversion of ethanol in the second step. The selection of cost-effective catalysts, such as CuCoMn and Cu/SiO_2, provides potential for significant economic advantages and sustainability throughout the process.

3. **Reaction Highlights**

Step 1: The catalyst CuCoMn facilitates a promising conversion of carbon monoxide into ethanol, establishing a strong initial framework for the pathway.
Step 2: The reaction features exceptional efficiency, achieving a 99% conversion rate of ethanol to acetaldehyde, showcasing the catalyst's effectiveness.
Step 3: The final step is notable for its selectivity of 80% in the conversion of acetaldehyde to crotonaldehyde, indicating a successful transformation in the synthesis pathway.

Figure S7. Crotonaldehyde Synthesis Pathway Generated with Domain-Specific Knowledge from Cat-KG. A three-step relay catalysis pathway for crotonaldehyde synthesis generated using structured reaction information from Cat-KG and processed by the *Relay Catalysis Analyzer*

Section S5. Relay Catalysis Pathways Identified and Recommended for Various Compounds

This section aims to present the relay catalysis pathways recommended by our system for various target compounds, focusing on the synthesis of molecules with significant industrial applications or potential. By analyzing the recommended results for several example compounds such as lower olefins, ethylene, ethylene glycol, oxalic acid, propylene glycol, crotonaldehyde, 1,3-butadiene, 1,4-butanediol, cis-2-butene, and 2,5-furandicarboxylic acid, we demonstrate the applicability and value of our method in discovering potential efficient relay catalysis pathways.

1. Recommendations for Lower Olefins Synthesis Pathways

(1) Two-Step Heterogeneous reaction pathway Query and Recommendation

Using Neo4j to perform Cypher queries on the Cat-KG, we obtained all candidate two-step heterogeneous reaction pathways for synthesizing lower olefins from Cat-KG, as shown in Figure S8.

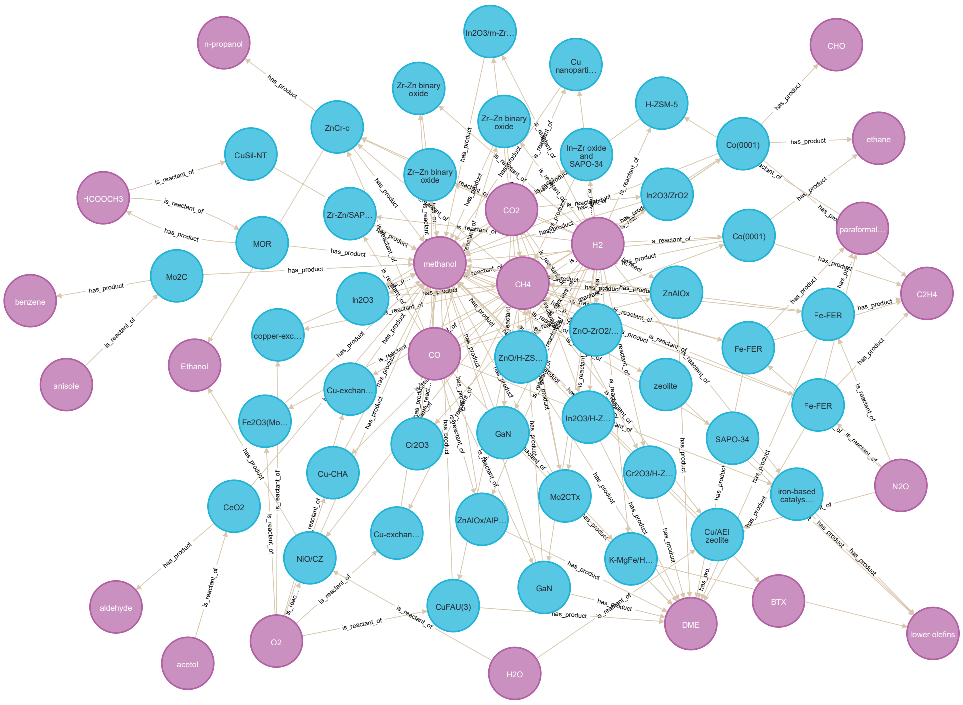

Figure S8. Two-step candidate pathways for synthesizing lower olefins. Blue nodes represent reaction nodes with catalyst names above, while purple nodes indicate reactants or products. The lines illustrate the relationships between the compounds and reaction nodes.

Applying our scoring rules, the system recommend a two-step relay catalysis pathway for lower olefins synthesis. Figure S9a illustrates this pathway in Neo4j representation. Figure S9b provides the synthesis pathway description converted into natural language by the GPTs.

(a)

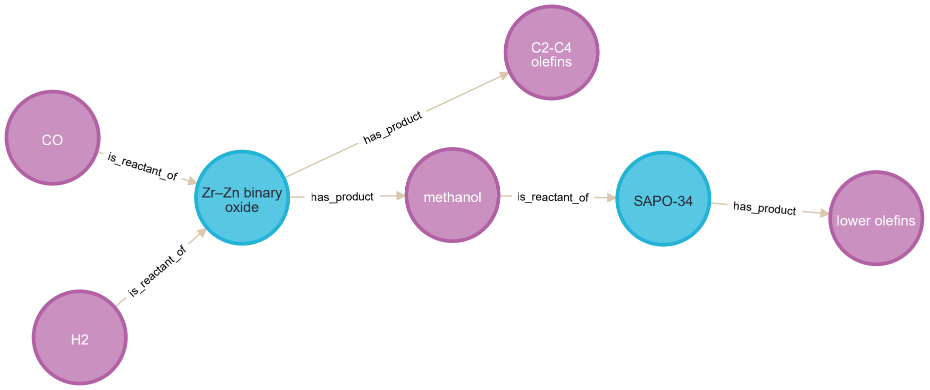

(b)

1. Catalytic Pathway Description

```css
CO + H₂ -------------> CH₃OH -------------> C₂-C₄ Olefins
     Cat.-[Zr-Zn binary oxide]     Cat.-[SAPO-34]
     Temp.-[673.0 K]             Temp.-[673.0 K]
```

2. Reaction Compatibility Analysis The temperatures for both steps are precisely aligned at 673.0 K, facilitating seamless transitions between the steps without the need for additional thermal management. The initial conversion of carbon monoxide to methanol proceeds with a conversion rate of 9.5%, while step two utilizes the produced methanol to yield lower olefins, achieving a significant yield of 85%. Notably, the same reactant, methanol, is reused in the second step, enhancing the efficiency of the overall process.
3. Reaction Highlights Step 2 demonstrates a remarkable yield of 85% in the conversion of methanol to lower olefins, showcasing the effectiveness of the SAPO-34 catalyst in facilitating this transformation. Additionally, the first step shows a noteworthy selectivity of 63% for C_2-C_4 olefins from methanol, underlining the potential of the Zr–Zn binary oxide catalyst in this pathway.

Figure S9. Relay Catalysis Pathway for Lower Olefins Synthesis. (a) The two-step relay catalysis pathway recommended by the system, visualized in Neo4j. (b) The natural language description of the pathway generated by GPTs.

(2) Three-Step Heterogeneous reaction pathway Query and Recommendation

Similarly, we performed a query for three-step heterogeneous reaction pathways for lower olefins from Cat-KG, as shown in Figure S10.

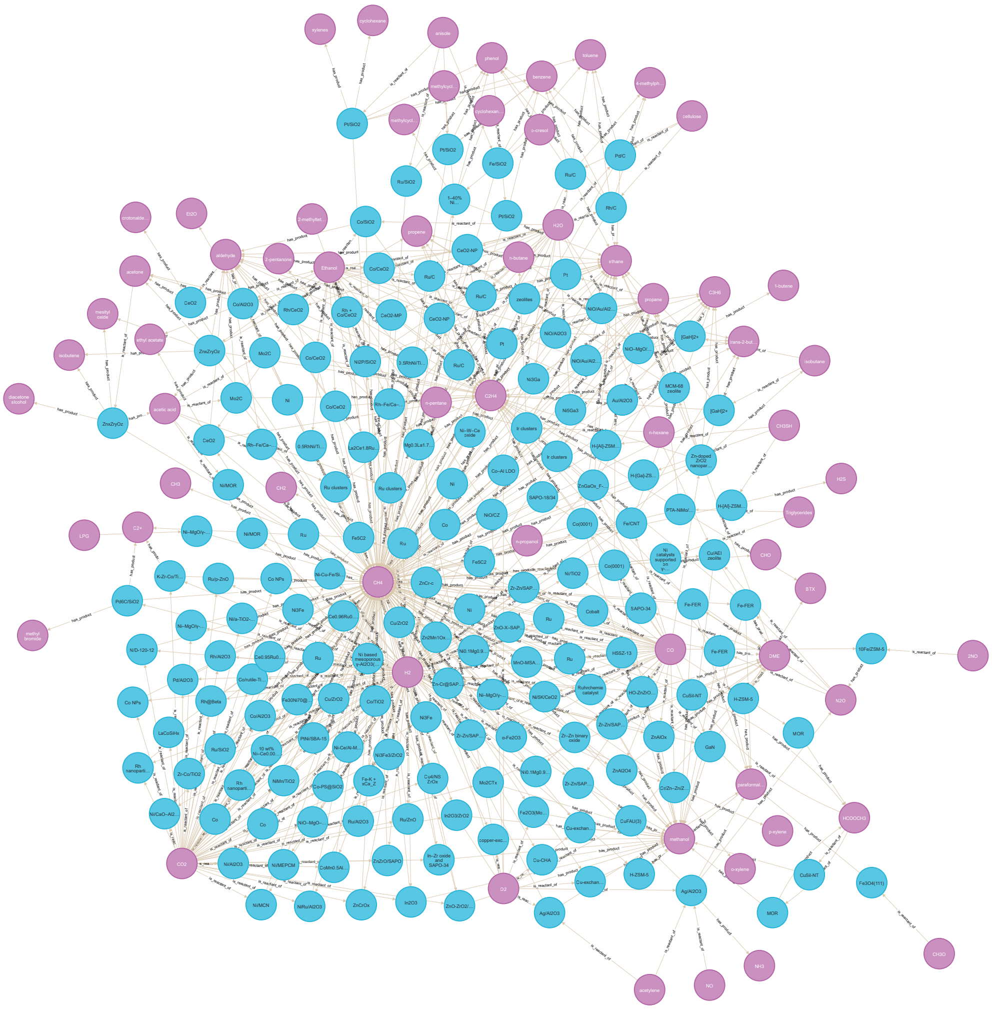

Figure S10. Three-step candidate pathways for synthesizing lower olefins. Blue nodes represent reaction nodes with catalyst names above, while purple nodes indicate reactants or products. The lines illustrate the relationships between the compounds and reaction nodes.

Applying our scoring rules, the system recommend a three-step relay catalysis pathway for lower olefins synthesis. Figure S11a illustrates this pathway in Neo4j

representation. Figure S11b provides the synthesis pathway description converted into natural language by the GPTs.

(a)

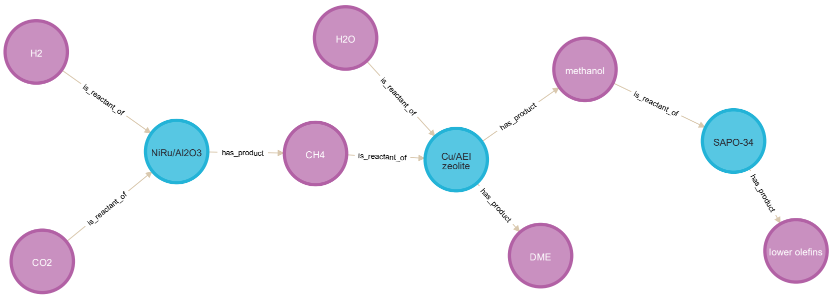

(b)

1. Catalytic Pathway Description

markdown | Copy code

```
CO2 + H2 ------------> CH4 ------------> Methanol ------------> Lower Olefins
      Cat.-[NiRu/Al2O3]        Cat.-[Cu/AEI zeolite]        Cat.-[SAPO-34]
      Temp.-[623.0 K]        Temp.-[623.0 K]            Temp.-[673.0 K]
```

2. Reaction Compatibility Analysis

The temperatures for the first two steps are identical at 623.0 K, ensuring a seamless transition without the need for thermal adjustments, thus enhancing the efficiency of the overall pathway. In the first step, CO2 is effectively converted to methane with a high conversion rate of 89.7% and 100% selectivity, allowing for its direct use as a reactant in the subsequent step. The second step utilizes the methane produced, demonstrating effective reactant utilization across steps. Notably, the third step operates at a slightly higher temperature of 673.0 K, which is compatible with the prior steps, allowing for a smooth reaction progression to the final target of lower olefins synthesis.

3. Reaction Highlights

Step 1: Achieves an impressive conversion of CO2 (89.7%) with complete selectivity for methane.
Step 2: Provides a noteworthy selectivity of 45% for methanol from methane.
Step 3: Offers a strong yield of 85% for lower olefins from methanol, marking a significant advantage in the conversion process.

Figure S11. Relay Catalysis Pathway for Lower Olefins Synthesis. (a) The three-step relay catalysis pathway recommended by the system, visualized in Neo4j. (b) The natural language description of the pathway generated by GPTs.

2. Recommendations for Ethylene Synthesis Pathways

(1) Two-Step Heterogeneous reaction pathway Query and Recommendation

Using Neo4j to perform Cypher queries on the Cat-KG, we obtained all candidate two-step heterogeneous reaction pathways for synthesizing ethylene from Cat-KG, as shown in Figure S12.

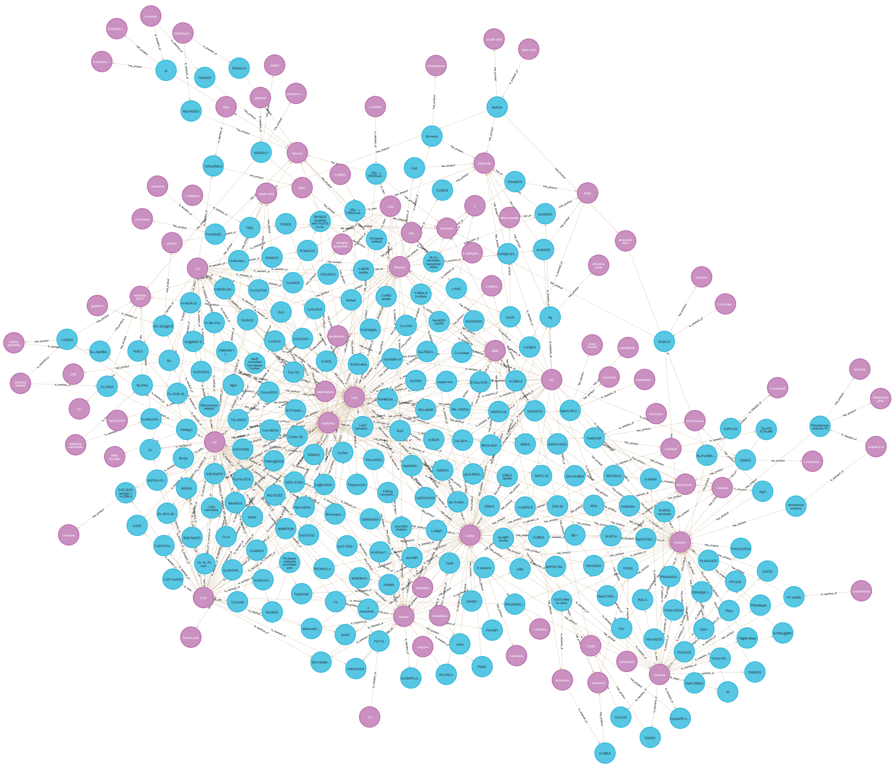

Figure S12. Two-step candidate pathways for synthesizing ethylene. Blue nodes represent reaction nodes with catalyst names above, while purple nodes indicate reactants or products. The lines illustrate the relationships between the compounds and reaction nodes.

Applying our scoring rules, the system recommend a two-step relay catalysis pathway for ethylene synthesis. Figure S13a illustrates this pathway in Neo4j representation. Figure S13b provides the synthesis pathway description converted into natural language by the GPTs.

(a)

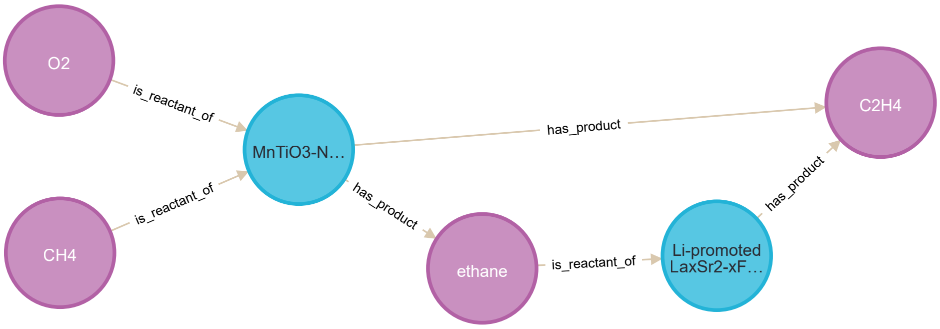

(b)

1. **Catalytic Pathway Description**

```css
CH4 + O2 ------------> C2H4 -----------> Ethylene
     Cat.-[MnTiO3-NW/SG]     Cat.-[Li-promoted LaxSr2-xFeO4-δ]
     Temp.-[973.0 K]      Temp.-[973.0 K]
```

2. **Reaction Compatibility Analysis** The temperatures for both steps are identical at 973.0 K, indicating optimal alignment and facilitating smooth transitions without the need for significant energy adjustments. The first step utilizes methane and oxygen, with a notable conversion of 35.6% for methane and selectivity of 60.8% for ethylene. The ethylene produced in step one is then used as the feed for step two, where ethane is converted with a conversion rate of 61%. The use of a consistent temperature across both steps enhances overall efficiency and reduces the risk of thermal destabilization.

3. **Reaction Highlights** Step 1 demonstrates a commendable selectivity of 60.8% towards ethylene from methane. In step 2, the reaction achieves an impressive selectivity of 90% for the conversion of ethane to ethylene, complemented by a substantial yield of 54.90%.

Figure S13. Relay Catalysis Pathway for Ethylene Synthesis. (a) The two-step relay catalysis pathway recommended by the system, visualized in Neo4j. (b) The natural language description of the pathway generated by GPTs.

(2) Three-Step Heterogeneous reaction pathway Query and Recommendation

Similarly, we performed a query for three-step heterogeneous reaction pathways for ethylene from Cat-KG, as shown in Figure S14.

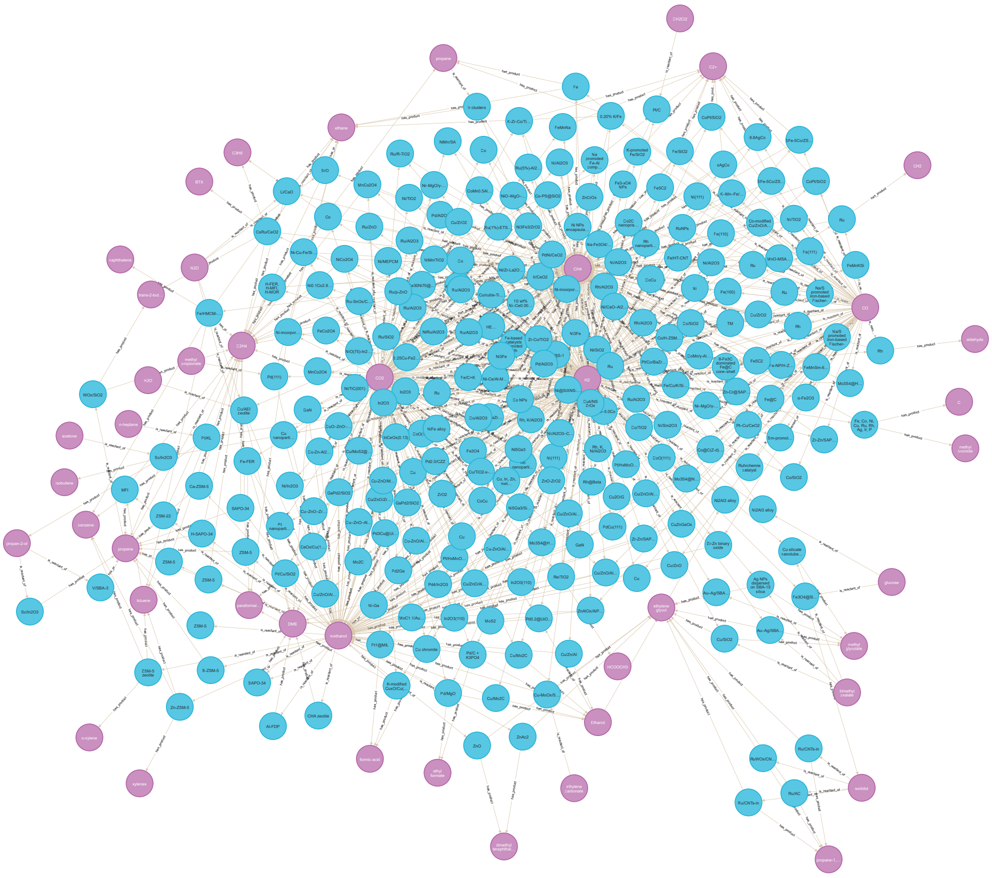

Figure S14. Three-step candidate pathways for synthesizing ethylene. Blue nodes represent reaction nodes with catalyst names above, while purple nodes indicate reactants or products. The lines illustrate the relationships between the compounds and reaction nodes.

Applying our scoring rules, the system recommend a three-step relay catalysis pathway for ethylene synthesis. Figure S15a illustrates this pathway in Neo4j representation. Figure S15b provides the synthesis pathway description converted into natural language by the GPTs.

(a)

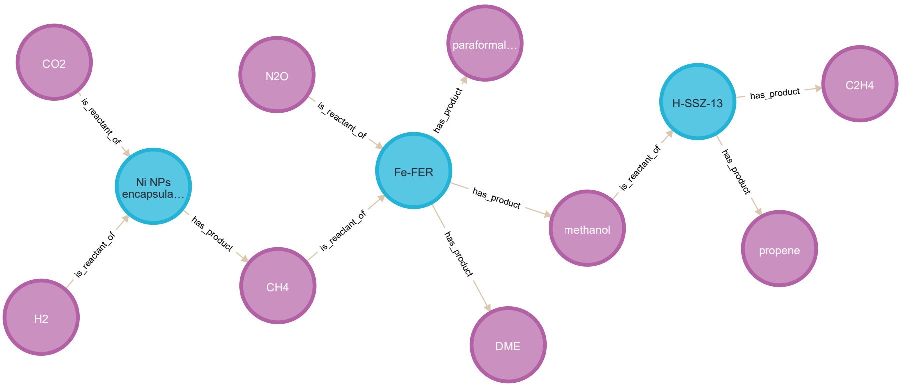

(b)

Certainly! Here's the detailed and academic description of the relay catalytic pathway for ethylene synthesis:

1. **Catalytic Pathway Description**

markdown

```
CO2 + H2 ------------> CH4 ------------> Methanol ------------> Ethylene
      Cat.-[Ni NPs in MIL-101]      Cat.-[Fe-FER]      Cat.-[H-SSZ-13]
      Temp.-[573.0 K]               Temp.-[573.0 K]   Temp.-[573.0 K]
```

2. **Reaction Compatibility Analysis**

The temperatures across all three steps are uniformly set at 573.0 K, ensuring optimal reaction conditions and facilitating seamless transitions between each step without the need for thermal adjustments. In this pathway, carbon dioxide is reused from step 1 to step 2, enhancing efficiency and sustainability. The catalyst Ni NPs in MIL-101 in the first step achieves complete conversion of CO_2 to methane with 100% selectivity, which serves as an effective precursor for the subsequent methane oxidation process utilizing N_2O in step 2, ultimately leading to the formation of methanol.

3. **Reaction Highlights**

Step 1: Complete conversion of carbon dioxide to methane (100% conversion) with 100% selectivity.
Step 2: Noteworthy 43.7% conversion of nitrous oxide, indicating significant methane oxidation potential.
Step 3: The transformation of methanol to ethylene is supported by the strong acid sites present in the H-SSZ-13 catalyst, promoting effective olefin production.

Figure S15. Relay Catalysis Pathway for Ethylene Synthesis. (a) The three-step relay catalysis pathway recommended by the system, visualized in Neo4j. (b) The natural language description of the pathway generated by GPTs.

3. Recommendations for Ethylene Glycol Synthesis Pathways

(1) Two-Step Heterogeneous reaction pathway Query and Recommendation

Using Neo4j to perform Cypher queries on the Cat-KG, we obtained all candidate two-step heterogeneous reaction pathways for synthesizing ethylene glycol from Cat-KG, as shown in Figure S16.

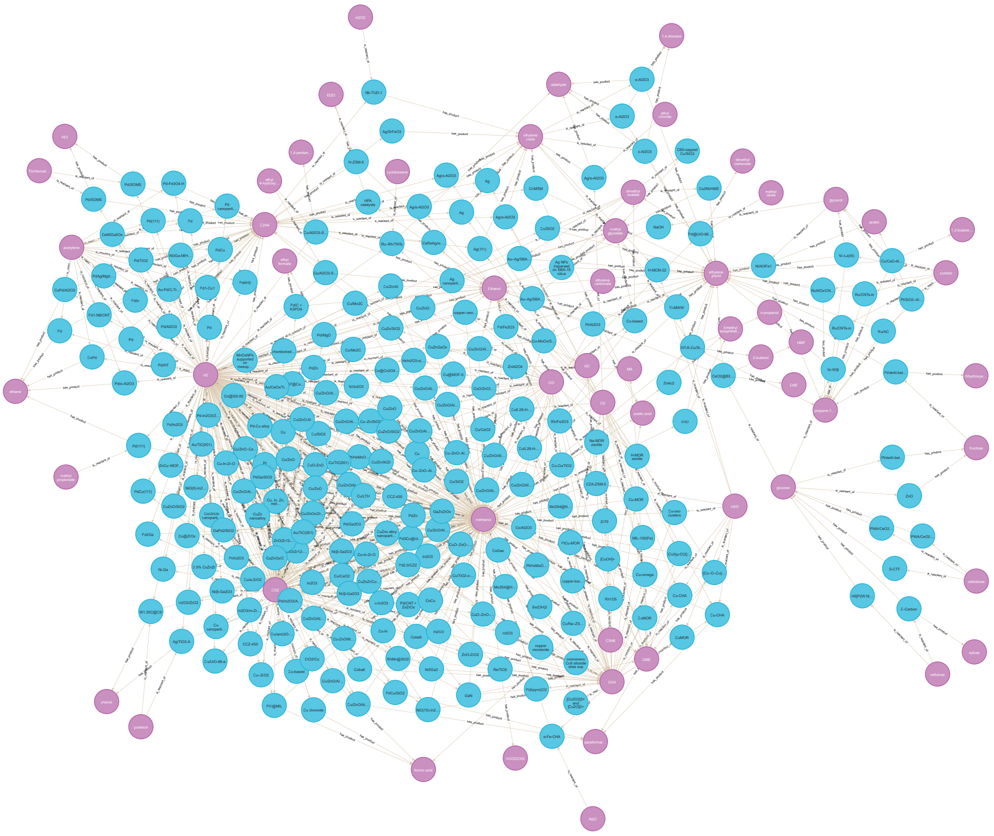

Figure S16. Two-step candidate pathways for synthesizing ethylene glycol. Blue nodes represent reaction nodes with catalyst names above, while purple nodes indicate reactants or products. The lines illustrate the relationships between the compounds and reaction nodes.

Applying our scoring rules, the system recommend a two-step relay catalysis pathway for ethylene glycol synthesis. Figure S17a illustrates this pathway in Neo4j representation. Figure S17b provides the synthesis pathway description converted into natural language by the GPTs.

(a)

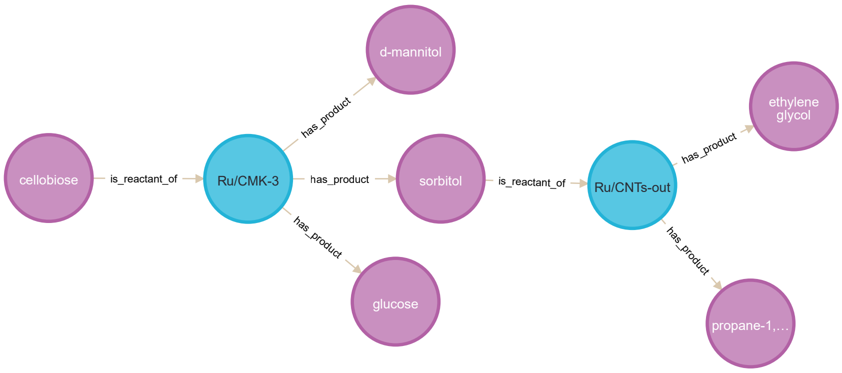

(b)

1. **Catalytic Pathway Description**

markdown   Copy code

```
Cellobiose -------------> Sorbitol -------------> Ethylene Glycol
       Cat.-[Ru/CMK-3]             Cat.-[Ru/CNTs-out]
       Temp.-[453.0 K]              Temp.-[478.0 K]
```

2. **Reaction Compatibility Analysis** The temperatures for each step, 453.0 K for the first step and 478.0 K for the second, indicate a manageable temperature gradient that allows for efficient reaction transitions without requiring substantial energy input. Notably, sorbitol is utilized as an intermediate in both steps, facilitating material reuse and enhancing overall process efficiency. Additionally, hydrogen gas is consistently employed at a pressure of 50 bar across both stages, reinforcing the hydrogen economy within this catalytic pathway. The choice of raw materials, particularly cellobiose and sorbitol, is favorable given their abundant availability and relatively low cost, contributing to the economic sustainability of the overall synthesis process.

3. **Reaction Highlights** Step 1: Achieves complete conversion of cellobiose to sorbitol with a high yield of 91.1%.
   Step 2: Delivers a notable yield of 58.5% for the conversion of sorbitol to ethylene glycol, indicating strong catalytic performance.

Figure S17. Relay Catalysis Pathway for Ethylene Glycol Synthesis. (a) The two-step relay catalysis pathway recommended by the system, visualized in Neo4j. (b) The natural language description of the pathway generated by GPTs.

(2) Three-Step Heterogeneous reaction pathway Query and Recommendation

Similarly, we performed a query for three-step heterogeneous reaction pathways for ethylene glycol from Cat-KG, as shown in Figure S18.

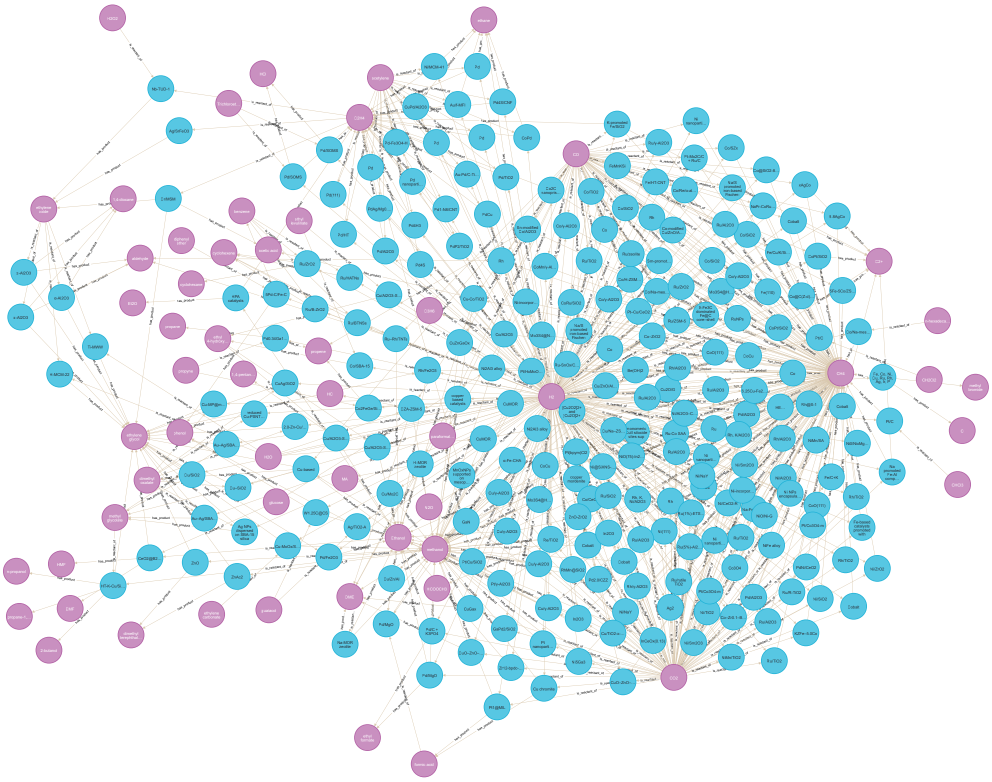

Figure S18. Three-step candidate pathways for synthesizing ethylene glycol. Blue nodes represent reaction nodes with catalyst names above, while purple nodes indicate reactants or products. The lines illustrate the relationships between the compounds and reaction nodes.

Applying our scoring rules, the system recommend a three-step relay catalysis pathway for ethylene glycol synthesis. Figure S19a illustrates this pathway in Neo4j representation. Figure S19b provides the synthesis pathway description converted into natural language by the GPTs.

(a)

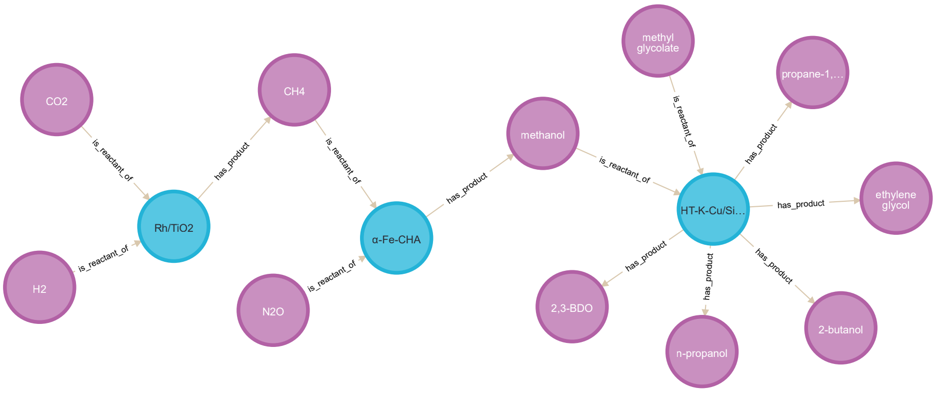

(b)

1. **Catalytic Pathway Description**

```markdown
CO₂ + H₂ -------------> CH₄ -------------> CH₃OH -------------> Ethylene Glycol
     Cat.-[Rh/TiO₂]           Cat.-[α-Fe-CHA]        Cat.-[HT-K-Cu/SiO₂]
     Temp.-[473.0 K]          Temp.-[473.0 K]         Temp.-[513.0 K]
```

2. **Reaction Compatibility Analysis** The temperatures in this catalytic pathway exhibit a close alignment, with the first two steps both occurring at 473.0 K, ensuring efficient transitions without significant thermal energy adjustments. In step 2, methane produced in step 1 is directly utilized as a reactant, demonstrating efficient resource utilization within the pathway. The transition from methanol to ethylene glycol in the final step occurs at a slightly higher temperature of 513.0 K, yet remains within a reasonable range, supporting smooth progressions throughout the relay process.

3. **Reaction Highlights** Step 3 showcases notable efficiency with a high conversion of methanol (93.9%) to ethylene glycol, achieving an impressive yield of 85.4% and a selectivity of 91.0%. This demonstrates the robustness of the catalytic system employed in this final transformation, contributing significantly to the overall efficacy of the pathway.

Figure S19. Relay Catalysis Pathway for Ethylene Glycol Synthesis. (a) The three-step relay catalysis pathway recommended by the system, visualized in Neo4j. (b) The natural language description of the pathway generated by GPTs.

4. Recommendations for Oxalic Acid Synthesis Pathways

(1) Two-Step Heterogeneous reaction pathway Query and Recommendation

Using Neo4j to perform Cypher queries on the Cat-KG, we obtained all candidate two-step heterogeneous reaction pathways for synthesizing oxalic acid from Cat-KG, as shown in Figure S20.

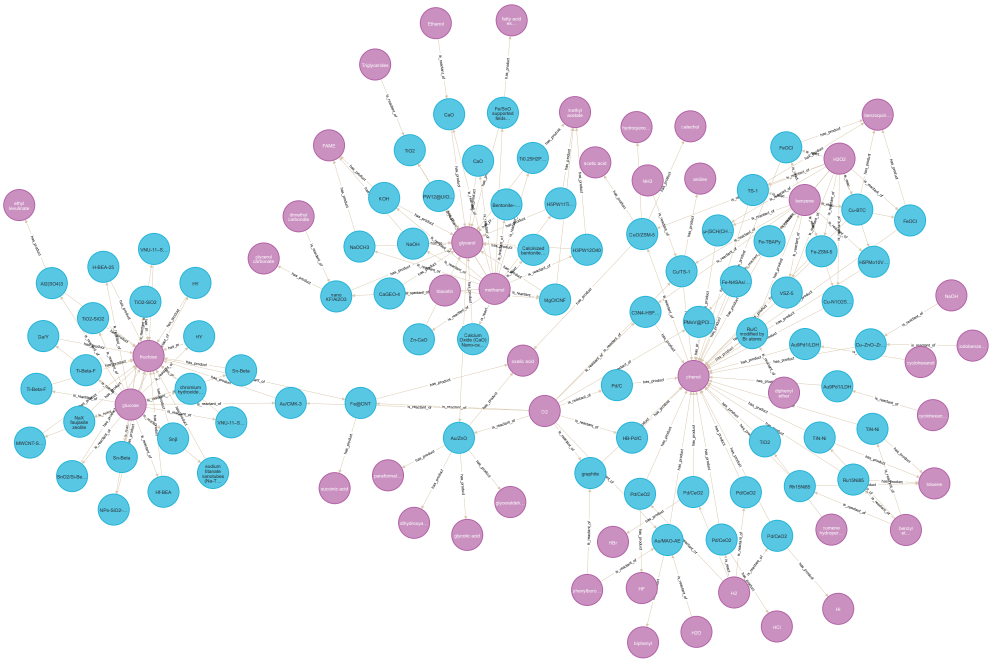

Figure S20. Two-step candidate pathways for synthesizing oxalic acid. Blue nodes represent reaction nodes with catalyst names above, while purple nodes indicate reactants or products. The lines illustrate the relationships between the compounds and reaction nodes.

Applying our scoring rules, the system recommend a two-step relay catalysis pathway for oxalic acid synthesis. Figure S21a illustrates this pathway in Neo4j representation. Figure S21b provides the synthesis pathway description converted into natural language by the GPTs.

(a)

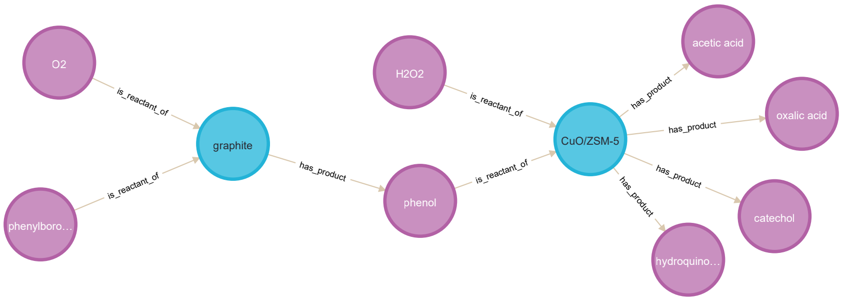

(b)

1. Catalytic Pathway Description

markdown   Copy code

```
Phenylboronic acid + O2 ----> Phenol ----> Oxalic acid + CO2 + H2O + Acetic acid + Hydroqu
          Cat.-[Graphite]                Cat.-[CuO/ZSM-5]
          Temp.-[353.0 K]                Temp.-[353.0 K]
```

2. Reaction Compatibility Analysis The temperatures are identical at 353.0 K for both steps, facilitating smooth transitions without significant thermal adjustments. The complete conversion of phenol in the second step enhances overall efficiency, while the use of water as a solvent in both reactions promotes sustainability. The graphite catalyst in the first step allows for high selectivity, while the copper oxide catalyst on ZSM-5 in the second step potentially offers advantages in the treatment of phenol.
3. Reaction Highlights Step 1: High yield of phenol (92%).
   Step 2: Complete conversion of phenol (100%).

Figure S21. Relay Catalysis Pathway for Oxalic Acid Synthesis. (a) The two-step relay catalysis pathway recommended by the system, visualized in Neo4j. (b) The natural language description of the pathway generated by GPTs.

(2) Three-Step Heterogeneous reaction pathway Query and Recommendation

Similarly, we performed a query for three-step heterogeneous reaction pathways for oxalic acid from Cat-KG, as shown in Figure S22.

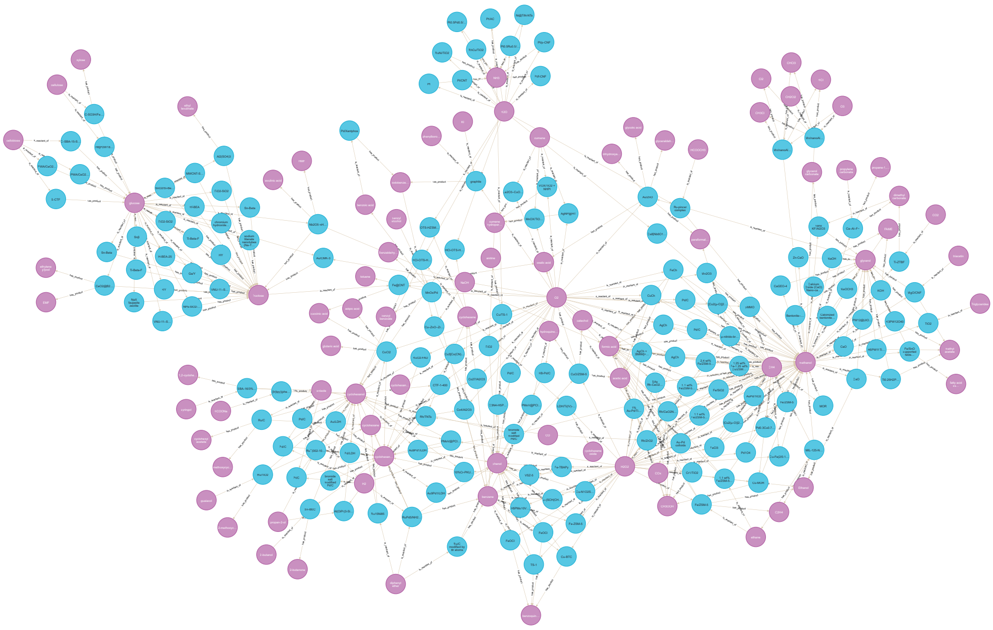

Figure S22. Three-step candidate pathways for synthesizing oxalic acid. Blue nodes represent reaction nodes with catalyst names above, while purple nodes indicate reactants or products. The lines illustrate the relationships between the compounds and reaction nodes.

Applying our scoring rules, the system recommend a three-step relay catalysis pathway for oxalic acid synthesis. Figure S23a illustrates this pathway in Neo4j representation. Figure S23b provides the synthesis pathway description converted into natural language by the GPTs.

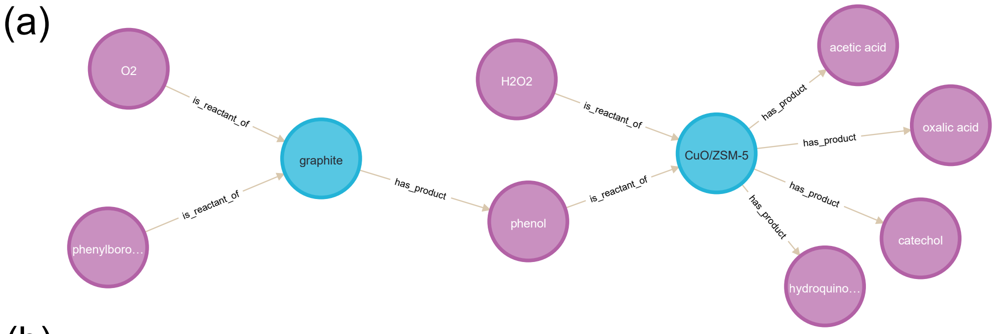

(b)

1. **Catalytic Pathway Description**

```markdown
Phenylboronic acid + KI + O₂ ----> Iodobenzene ----> Phenol ----> Oxalic Acid
             Cat.-[Graphite]             Cat.-[Cu-ZnO-ZrO₂]          Cat.-[CuO/ZSM-5]
             Temp.-[353.0 K]              Temp.-[383.0 K]             Temp.-[353.0 K]
```

2. **Reaction Compatibility Analysis**

The temperatures across the three steps exhibit a strategic alignment, with the first and third steps operating at 353.0 K, while the second step occurs at a slightly higher temperature of 383.0 K. This difference is minimal and facilitates smooth transitions, ensuring that the reactions can proceed efficiently without drastic thermal adjustments. In this pathway, iodobenzene is effectively reused in the second step after its formation in the first step, optimizing the overall material utilization. The first step utilizes potassium iodide and oxygen, while the second employs sodium hydroxide, highlighting a diverse yet efficient use of reagents. Economically, phenylboronic acid and iodobenzene are valuable intermediates, offering high potential for recycling within the pathway, thus enhancing sustainability and cost-effectiveness.

3. **Reaction Highlights**

Step 1: The conversion of phenylboronic acid to iodobenzene demonstrates an impressive yield of 92%.
Step 2: The hydroxylation of iodobenzene to phenol achieves a remarkable yield of 99%.
Step 3: The transformation of phenol into oxalic acid, while lacking specific yield data, capitalizes on a complete conversion of phenol, showcasing the efficiency of the reaction.

Figure S23. Relay Catalysis Pathway for Oxalic Acid Synthesis. (a) The three-step relay catalysis pathway recommended by the system, visualized in Neo4j. (b) The natural language description of the pathway generated by GPTs.

5. Recommendations for Propylene Glycol Synthesis Pathways

(1) Two-Step Heterogeneous reaction pathway Query and Recommendation

Using Neo4j to perform Cypher queries on the Cat-KG, we obtained all candidate two-step heterogeneous reaction pathways for synthesizing propylene glycol from Cat-KG, as shown in Figure S24.

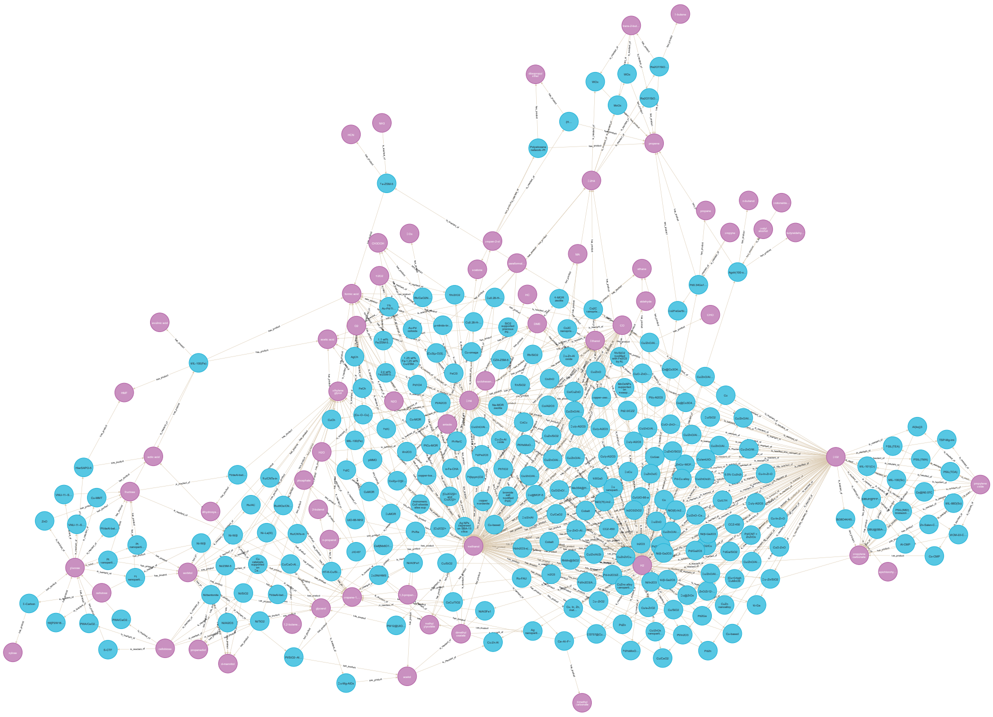

Figure S24. Two-step candidate pathways for synthesizing propylene glycol. Blue nodes represent reaction nodes with catalyst names above, while purple nodes indicate reactants or products. The lines illustrate the relationships between the compounds and reaction nodes.

Applying our scoring rules, the system recommend a two-step relay catalysis pathway for propylene glycol synthesis. Figure S25a illustrates this pathway in Neo4j representation. Figure S25b provides the synthesis pathway description converted into natural language by the GPTs.

(a)

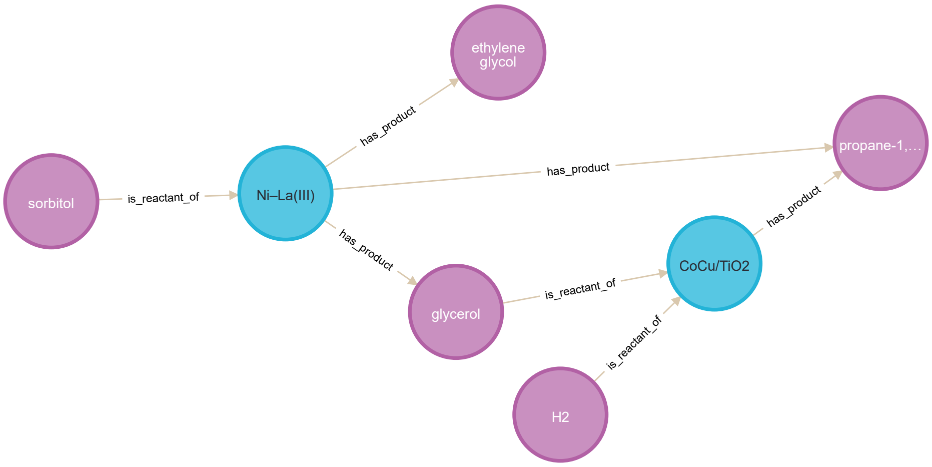

(b)

1. Catalytic Pathway Description

```css
sorbitol ----> glycerol ----> 1,2-propanediol
      Cat.-[Ni-La(III)]       Cat.-[CoCu/TiO2]
      Temp.-[518.0 K]        Temp.-[523.0 K]
```

2. Reaction Compatibility Analysis The temperatures for the two steps are closely aligned, with 518.0 K and 523.0 K, allowing for efficient transitions between the steps. Glycerol serves as a crucial intermediate, reused in the second step, enhancing overall economic efficiency. The use of hydrogen gas is consistent across both reactions, further promoting sustainability.
3. Reaction Highlights Step 1: Complete conversion of sorbitol (100%).
   Step 2: High yield of 1,2-propanediol at 69.5%.

Figure S25. Relay Catalysis Pathway for Propylene Glycol Synthesis. (a) The two-step relay catalysis pathway recommended by the system, visualized in Neo4j. (b) The natural language description of the pathway generated by GPTs.

(2) Three-Step Heterogeneous reaction pathway Query and Recommendation

Similarly, we performed a query for three-step heterogeneous reaction pathways for propylene glycol from Cat-KG, as shown in Figure S26.

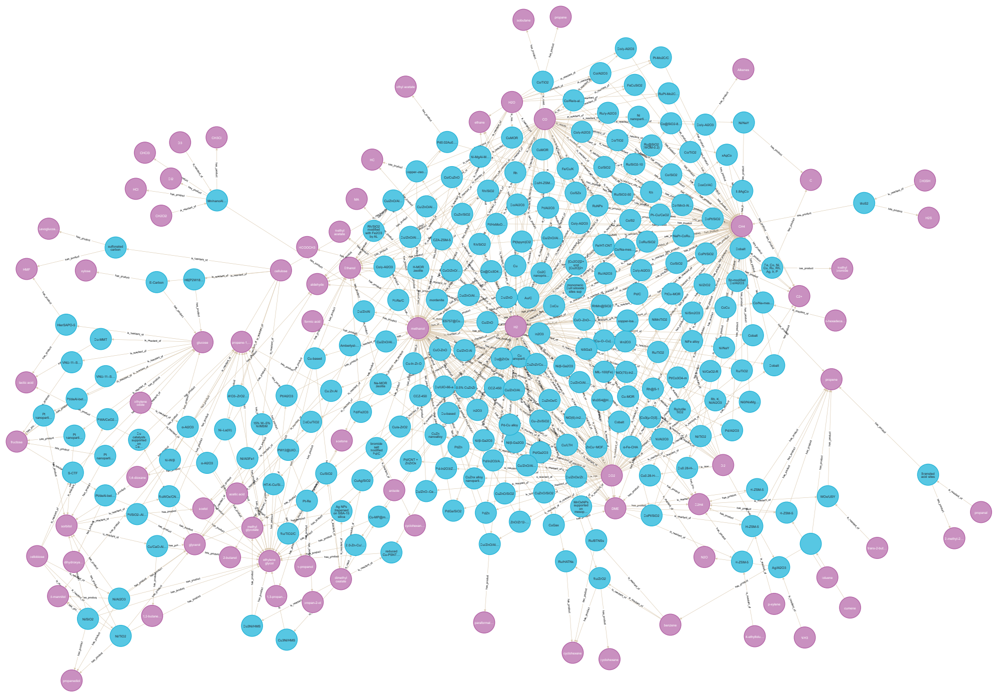

Figure S26. Three-step candidate pathways for synthesizing propylene glycol. Blue nodes represent reaction nodes with catalyst names above, while purple nodes indicate reactants or products. The lines illustrate the relationships between the compounds and reaction nodes.

Applying our scoring rules, the system recommend a three-step relay catalysis pathway for propylene glycol synthesis. Figure S27a illustrates this pathway in Neo4j representation. Figure S27b provides the synthesis pathway description converted into natural language by the GPTs.

(a)

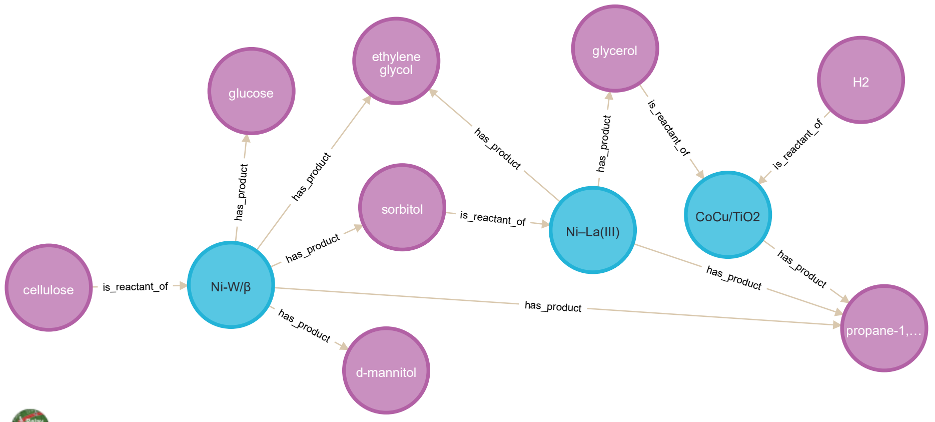

(b)

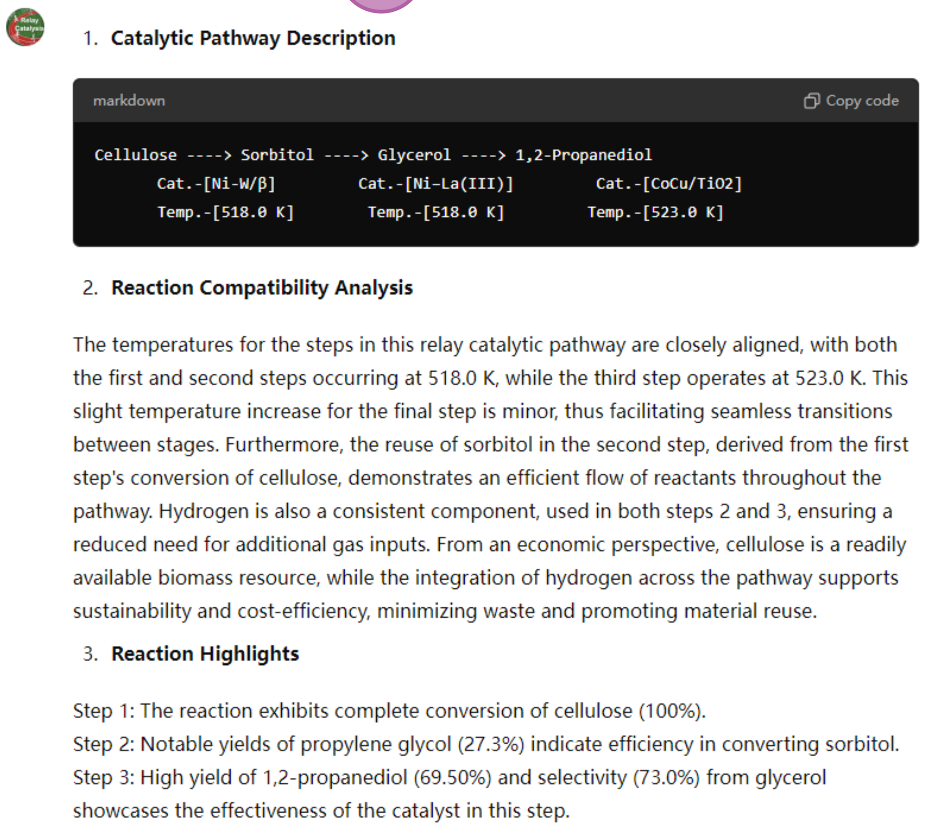

1. **Catalytic Pathway Description**

```markdown
Cellulose ----> Sorbitol ----> Glycerol ----> 1,2-Propanediol
       Cat.-[Ni-W/β]        Cat.-[Ni-La(III)]        Cat.-[CoCu/TiO2]
       Temp.-[518.0 K]        Temp.-[518.0 K]        Temp.-[523.0 K]
```

2. **Reaction Compatibility Analysis**

The temperatures for the steps in this relay catalytic pathway are closely aligned, with both the first and second steps occurring at 518.0 K, while the third step operates at 523.0 K. This slight temperature increase for the final step is minor, thus facilitating seamless transitions between stages. Furthermore, the reuse of sorbitol in the second step, derived from the first step's conversion of cellulose, demonstrates an efficient flow of reactants throughout the pathway. Hydrogen is also a consistent component, used in both steps 2 and 3, ensuring a reduced need for additional gas inputs. From an economic perspective, cellulose is a readily available biomass resource, while the integration of hydrogen across the pathway supports sustainability and cost-efficiency, minimizing waste and promoting material reuse.

3. **Reaction Highlights**

Step 1: The reaction exhibits complete conversion of cellulose (100%).
Step 2: Notable yields of propylene glycol (27.3%) indicate efficiency in converting sorbitol.
Step 3: High yield of 1,2-propanediol (69.50%) and selectivity (73.0%) from glycerol showcases the effectiveness of the catalyst in this step.

Figure S27. Relay Catalysis Pathway for Propylene Glycol Synthesis. (a) The three-step relay catalysis pathway recommended by the system, visualized in Neo4j. (b) The natural language description of the pathway generated by GPTs.

6. Recommendations for Crotonaldehyde Synthesis Pathways

(1) Two-Step Heterogeneous reaction pathway Query and Recommendation

Using Neo4j to perform Cypher queries on the Cat-KG, we obtained all candidate two-step heterogeneous reaction pathways for synthesizing crotonaldehyde from Cat-KG, as shown in Figure S28.

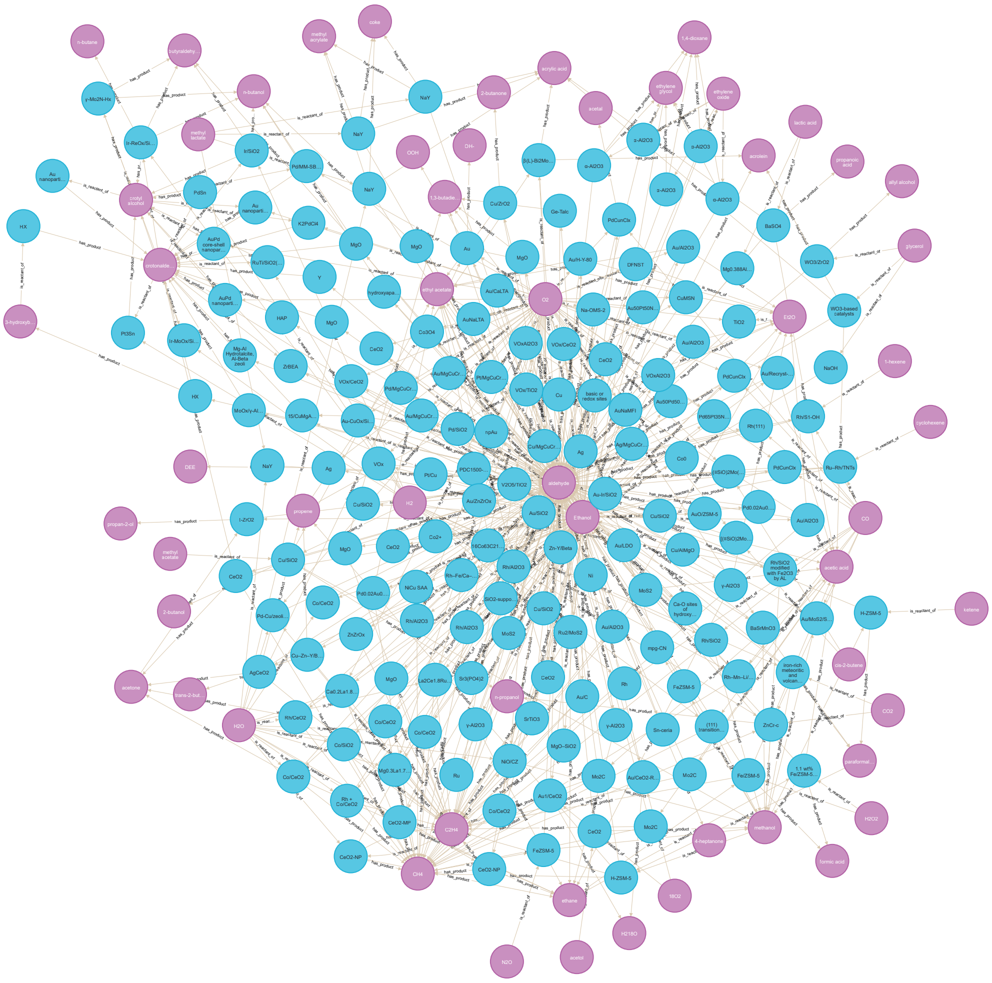

Figure S28. Relay Catalysis Pathway for Crotonaldehyde Synthesis. (a) The two-step relay catalysis pathway recommended by the system, visualized in Neo4j. (b) The natural language description of the pathway generated by GPTs.

Applying our scoring rules, the system recommend a two-step relay catalysis pathway for crotonaldehyde synthesis. Figure S29a illustrates this pathway in Neo4j representation. Figure S29b provides the synthesis pathway description converted into natural language by the GPTs.

(a)

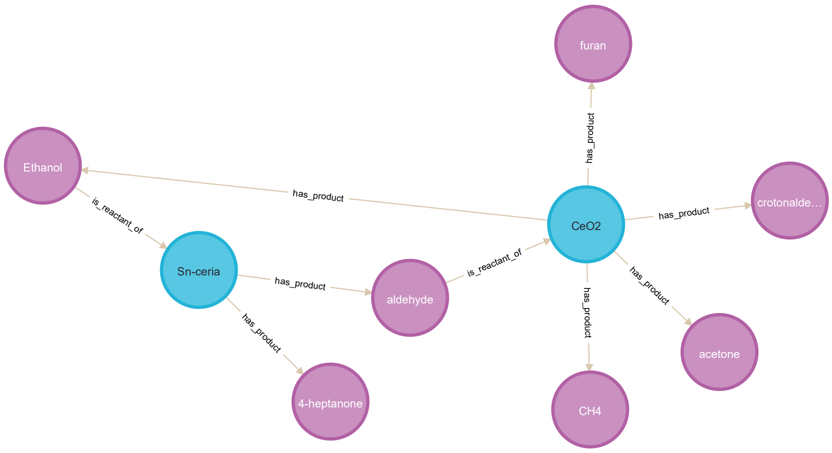

(b)

1. Catalytic Pathway Description

```markdown
Ethanol ----> Acetaldehyde ----> Crotonaldehyde
   Cat.-[2% Sn-ceria]         Cat.-[CeO₂]
   Temp.-[673.0 K]          Temp.-[673.0 K]
```

2. Reaction Compatibility Analysis
   The temperatures are well-aligned at 673.0 K for both steps, facilitating seamless transitions between them. The reuse of acetaldehyde from step 1 in step 2 enhances the overall efficiency of the pathway, while the presence of water as a solvent in both steps contributes to a sustainable reaction environment.

3. Reaction Highlights
   Step 1: Notable selectivity of 86% for the production of 4-heptanone from ethanol. Step 2: The formation of crotonaldehyde as a target compound underscores the efficiency of the catalyst employed.

Figure S29. Two-step candidate pathways for synthesizing crotonaldehyde. Blue nodes represent reaction nodes with catalyst names above, while purple nodes indicate reactants or products. The lines illustrate the relationships between the compounds and reaction nodes.

(2) Three-Step Heterogeneous reaction pathway Query and Recommendation

Similarly, we performed a query for three-step heterogeneous reaction pathways for crotonaldehyde from Cat-KG, as shown in Figure S30.

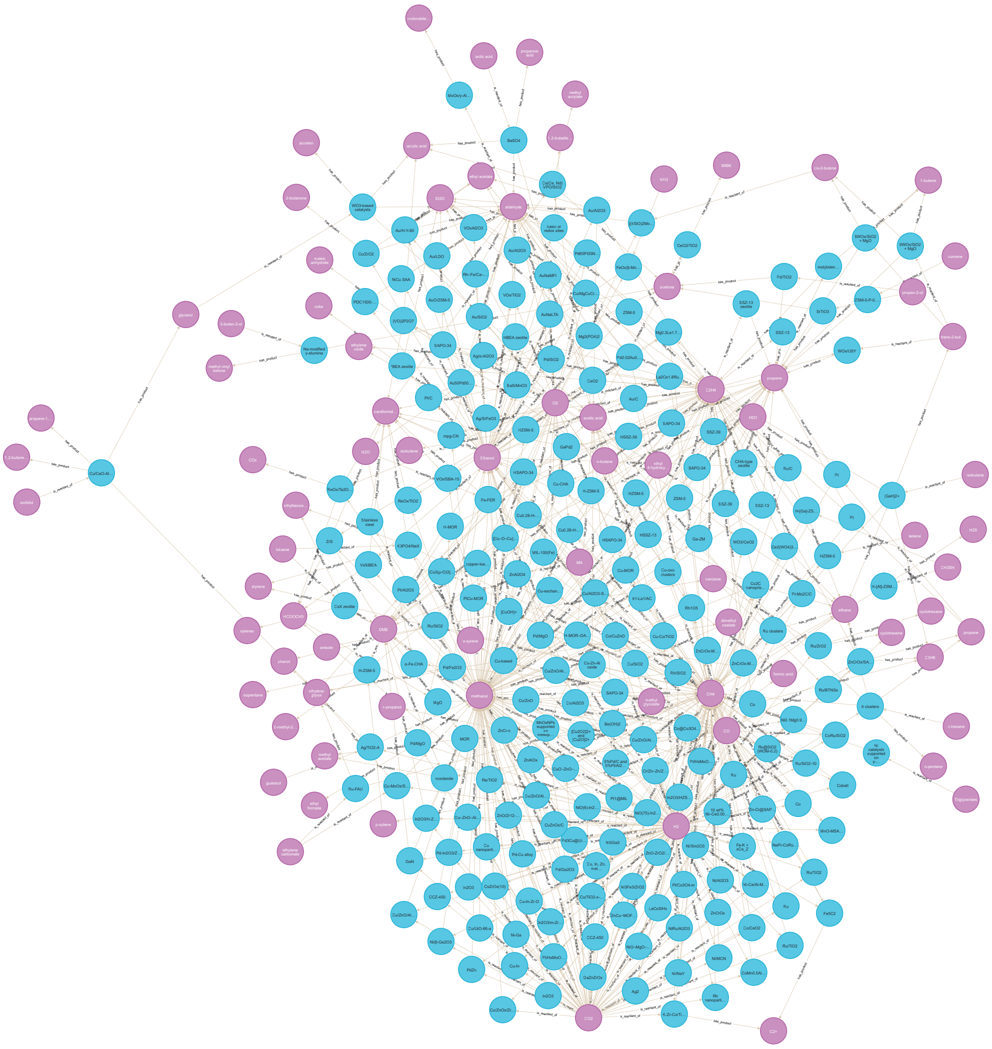

Figure S30. Three-step candidate pathways for synthesizing crotonaldehyde. Blue nodes represent reaction nodes with catalyst names above, while purple nodes indicate reactants or products. The lines illustrate the relationships between the compounds and reaction nodes.

Applying our scoring rules, the system recommend a three-step relay catalysis pathway for crotonaldehyde synthesis. Figure S31a illustrates this pathway in Neo4j

representation. Figure S31b provides the synthesis pathway description converted into natural language by the GPTs.

(a)

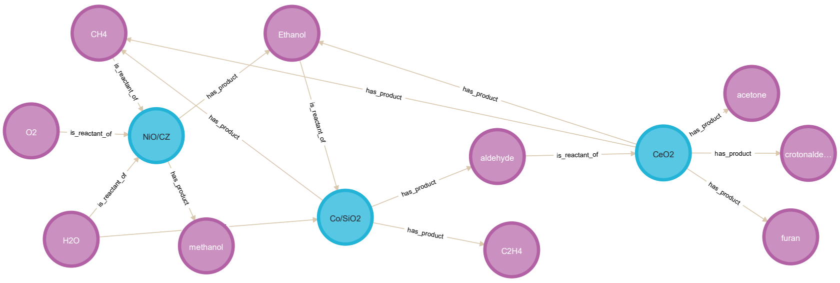

(b)

1. **Catalytic Pathway Description**

markdown — Copy code

```
Methane + O₂ + H₂O --------> Ethanol --------> Acetaldehyde --------> Crotonaldehyde
          Cat.-[NiO/CZ]            Cat.-[Co/SiO₂]           Cat.-[CeO₂]
          Temp.-[723.0 K]        Temp.-[723.0 K]         Temp.-[673.0 K]
```

2. **Reaction Compatibility Analysis** The temperatures of the first two steps are well-aligned at 723.0 K, ensuring that transitions between these steps occur without significant energy adjustments. However, the third step's temperature is 673.0 K, which is slightly lower, potentially requiring a moderate cooling phase to maintain optimal conditions. Ethanol is reused as an intermediate in the transition from the first to the second step, promoting material efficiency. The overall economic efficiency is enhanced by the use of methane as a readily available and low-cost feedstock, alongside the catalyst systems which demonstrate high turnover frequencies and surface areas, thereby potentially reducing operational costs.

3. **Reaction Highlights** Step 1: Notable conversion of methane with a 15% yield of ethanol, exhibiting a selectivity of 24%.
   Step 2: Effective conversion of ethanol, producing multiple valuable intermediates including hydrogen, CO, and acetaldehyde, facilitating further downstream processing.
   Step 3: The transformation of acetaldehyde into crotonaldehyde highlights the potential for selective synthesis in a controlled manner, emphasizing the utility of the CeO_2 catalyst.

Figure S31. Relay Catalysis Pathway for Crotonaldehyde Synthesis. (a) The three-step relay catalysis pathway recommended by the system, visualized in Neo4j. (b) The natural language description of the pathway generated by GPTs.

7. Recommendations for 1,3-Butadiene Synthesis Pathways

(1) Two-Step Heterogeneous reaction pathway Query and Recommendation

Using Neo4j to perform Cypher queries on the Cat-KG, we obtained all candidate two-step heterogeneous reaction pathways for synthesizing 1,3-butadiene from Cat-KG, as shown in Figure S32.

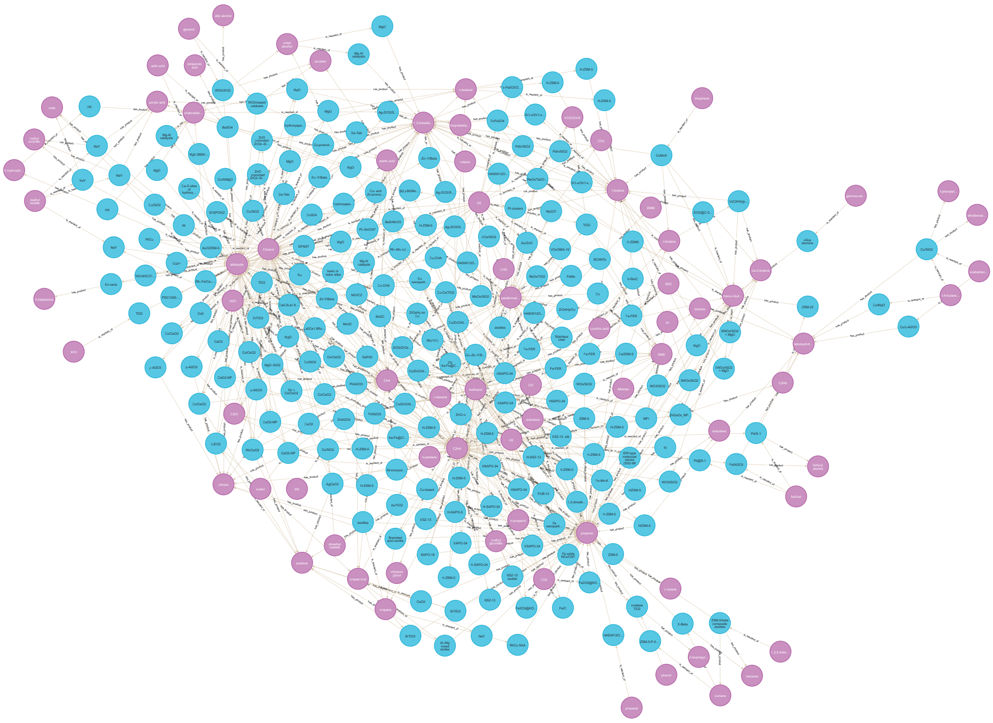

Figure S32. Two-step candidate pathways for synthesizing 1,3-butadiene. Blue nodes represent reaction nodes with catalyst names above, while purple nodes indicate reactants or products. The lines illustrate the relationships between the compounds and reaction nodes.

Applying our scoring rules, the system recommend a two-step relay catalysis pathway for 1,3-butadiene synthesis. Figure S33a illustrates this pathway in Neo4j representation. Figure S33b provides the synthesis pathway description converted into natural language by the GPTs.

(a)

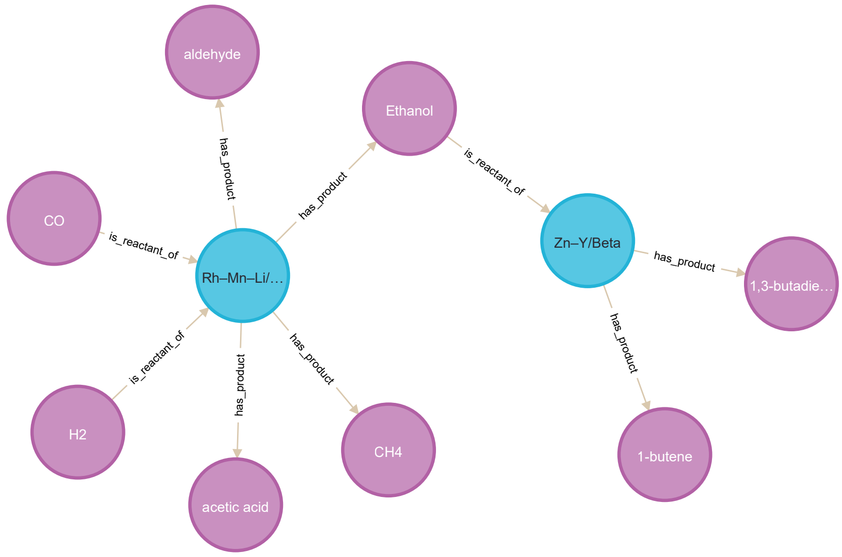

(b)

1. Catalytic Pathway Description

markdown Copy code

```
CO + H₂ ----> Ethanol ----> 1,3-Butadiene
      Cat.-[Rh-Mn-Li/SiO2]      Cat.-[Zn-Y/Beta]
      Temp.-[573.0 K]          Temp.-[588.0 K]
```

2. Reaction Compatibility Analysis The temperatures are well-aligned across the steps, with 573.0 K for the first step and 588.0 K for the second, ensuring smooth transitions without significant energy adjustments. Ethanol is efficiently reused in the second step after its formation in the first, contributing to the overall sustainability of the pathway.

3. Reaction Highlights Step 1: Noteworthy selectivity for carbon monoxide conversion to ethanol (21.5%).
   Step 2: High conversion of ethanol (87%) with a strong selectivity for butadiene (60%).

Figure S33. Relay Catalysis Pathway for 1,3-Butadiene Synthesis. (a) The two-step relay catalysis pathway recommended by the system, visualized in Neo4j. (b) The natural language description of the pathway generated by GPTs.

(2) Three-Step Heterogeneous reaction pathway Query and Recommendation

Similarly, we performed a query for three-step heterogeneous reaction pathways for 1,3-butadiene from Cat-KG, as shown in Figure S34.

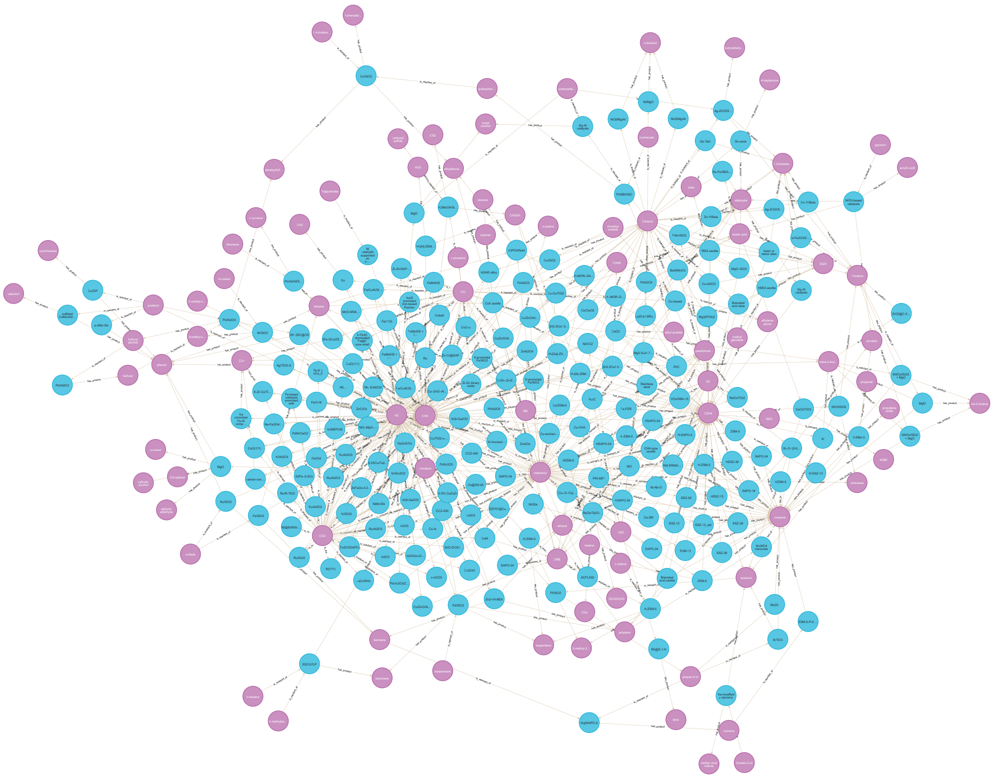

Figure S34. Three-step candidate pathways for synthesizing 1,3-butadiene. Blue nodes represent reaction nodes with catalyst names above, while purple nodes indicate reactants or products. The lines illustrate the relationships between the compounds and reaction nodes.

Applying our scoring rules, the system recommend a three-step relay catalysis pathway for 1,3-butadiene synthesis. Figure S35a illustrates this pathway in Neo4j representation. Figure S35b provides the synthesis pathway description converted into natural language by the GPTs.

(a)

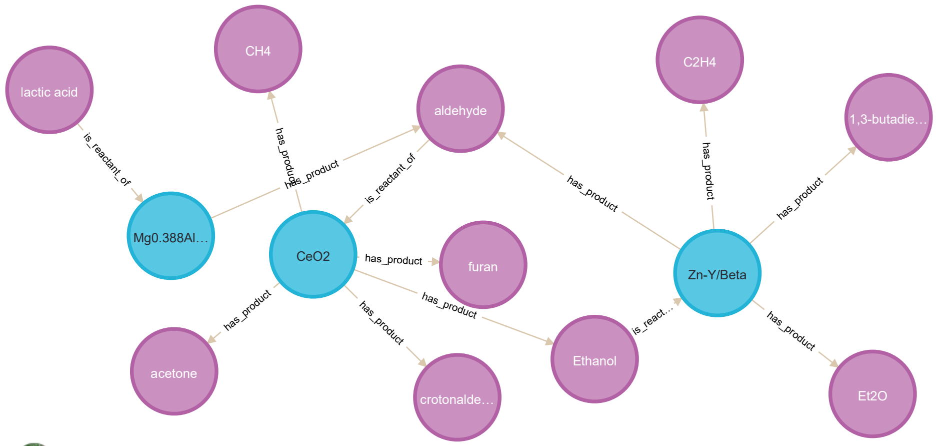

(b)

1. **Catalytic Pathway Description**

```css
lactic acid ----> acetaldehyde ----> ethanol ----> 1,3-Butadiene
    Cat.-[Mg0.388Al2.408O4]     Cat.-[CeO2]          Cat.-[Zn-Y/Beta]
    Temp.-[653.0 K]            Temp.-[673.0 K]      Temp.-[673.0 K]
```

2. **Reaction Compatibility Analysis** The temperatures across the three steps are closely aligned, with step 1 at 653.0 K and both steps 2 and 3 at 673.0 K, facilitating smooth transitions between reactions without significant thermal adjustments. Acetaldehyde, produced in the first step, is reused in the second step, promoting economic efficiency and sustainability. The use of ethanol in the third step, which shows high conversion at 95.8%, further enhances the pathway's viability. The catalysts employed demonstrate varying surface areas and properties, with the $Mg_{0.388}Al_{2.408}O_4$ catalyst exhibiting weak-medium acidity, while the CeO_2 catalyst presents strong basic sites, supporting the diverse reaction conditions.

3. **Reaction Highlights** Step 1: Achieves 100% conversion of lactic acid with a selectivity of 93% for acetaldehyde.
   Step 3: Delivers a high conversion rate of ethanol at 95.8% and a selectivity of 71.4% for 1,3-butadiene.

Figure S35. Relay Catalysis Pathway for 1,3-Butadiene Synthesis. (a) The three-step relay catalysis pathway recommended by the system, visualized in Neo4j. (b) The natural language description of the pathway generated by GPTs.

8. Recommendations for 1,4-Butanediol Synthesis Pathways

(1) Two-Step Heterogeneous reaction pathway Query and Recommendation

Using Neo4j to perform Cypher queries on the Cat-KG, we obtained all candidate two-step heterogeneous reaction pathways for synthesizing 1,4-butanediol from Cat-KG, as shown in Figure S36.

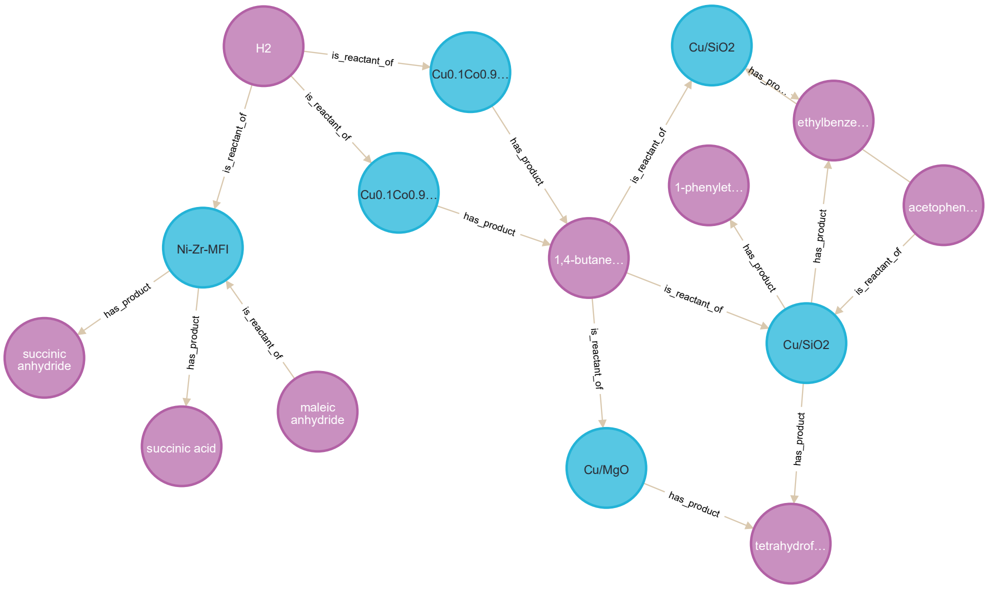

Figure S36. Two-step candidate pathways for synthesizing 1,4-butanediol. Blue nodes represent reaction nodes with catalyst names above, while purple nodes indicate reactants or products. The lines illustrate the relationships between the compounds and reaction nodes.

Applying our scoring rules, the system recommend a two-step relay catalysis pathway for 1,4-butanediol synthesis. Figure S37a illustrates this pathway in Neo4j representation. Figure S37b provides the synthesis pathway description converted into natural language by the GPTs.

(a)

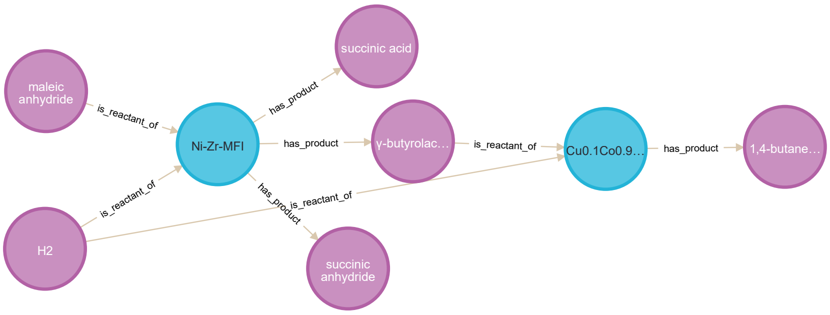

(b)

1. **Catalytic Pathway Description**

markdown

```
Maleic Anhydride + H₂ ----> γ-Butyrolactone ----> 1,4-Butanediol
       Cat.-[Ni-Zr-MFI]       Cat.-[Cu0.1Co0.9/TiO2]
       Temp.-[473.0 K]       Temp.-[413.0 K]
```

2. **Reaction Compatibility Analysis**

The temperatures of the two steps in this relay catalytic pathway exhibit a notable difference, with the first step occurring at 473.0 K and the second at 413.0 K. While the initial reaction is significantly higher, this gradient indicates the potential for effective cooling during the transition between steps, which could facilitate operational efficiency. Hydrogen is reused as a reactant in both steps, enhancing overall process sustainability. The economic efficiency of this pathway is supported by the high yield of γ-butyrolactone at 95.6% in the first step, suggesting a favorable starting point for subsequent transformations. Furthermore, the use of abundant and cost-effective materials, including the hierarchical Ni-Zr-MFI catalyst, contributes to a potentially sustainable approach to synthesizing 1,4-butanediol.

3. **Reaction Highlights**

Step 1: Achieves a remarkable conversion of maleic anhydride at 100% and a high yield of γ-butyrolactone at 95.6%.
Step 2: Demonstrates impressive yield of 1,4-butanediol at 95%.

Figure S37. Relay Catalysis Pathway for 1,4-Butanediol Synthesis. (a) The two-step relay catalysis pathway recommended by the system, visualized in Neo4j. (b) The natural language description of the pathway generated by GPTs.

(2) Three-Step Heterogeneous reaction pathway Query and Recommendation

Similarly, we performed a query for three-step heterogeneous reaction pathways for 1,4-butanediol from Cat-KG, as shown in Figure S38.

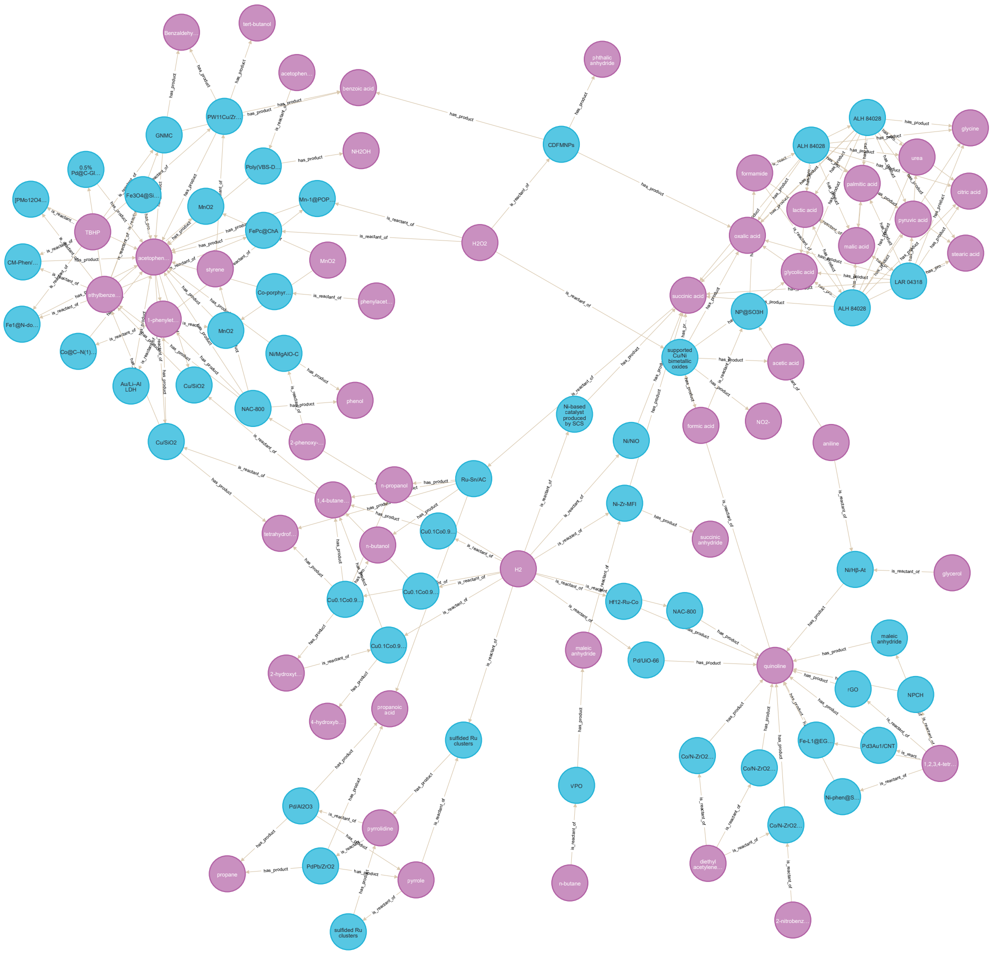

Figure S38. Three-step candidate pathways for synthesizing 1,4-butanediol. Blue nodes represent reaction nodes with catalyst names above, while purple nodes indicate reactants or products. The lines illustrate the relationships between the compounds and reaction nodes.

Applying our scoring rules, the system recommend a three-step relay catalysis pathway for 1,4-butanediol synthesis. Figure S39a illustrates this pathway in Neo4j representation. Figure S39b provides the synthesis pathway description converted into natural language by the GPTs.

(a)

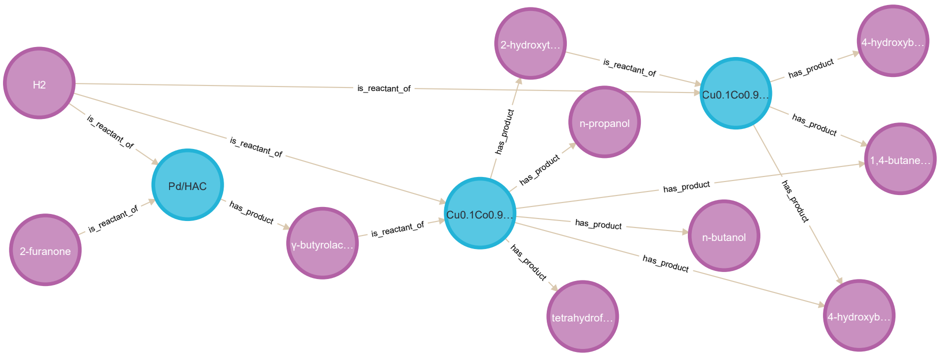

(b)

1. **Catalytic Pathway Description**

```markdown
2-furanone + H₂ ----> γ-butyrolactone ----> 2-hydroxytetrahydrofuran ----> 1,4-Butanediol
    Cat.-[Pd/HAC]       Cat.-[Cu0.1Co0.9/TiO₂]       Cat.-[Cu0.1Co0.9/TiO₂]
    Temp.-[298 K]      Temp.-[413.0 K]           Temp.-[413.0 K]
```

2. **Reaction Compatibility Analysis** The temperatures across the steps exhibit a significant contrast, with step 1 at 298 K and steps 2 and 3 at 413.0 K. This temperature increase may necessitate careful management of thermal conditions to facilitate effective transitions. Hydrogen serves as a common reactant in steps 1, 2, and 3, promoting resource efficiency through its reuse. The economic analysis indicates that the utilization of palladium supported on humin-derived activated carbon and the bimetallic copper-cobalt catalyst over titanium dioxide provides a pathway that leverages renewable feedstocks and potentially lowers overall costs, particularly given the high yield of 1,4-butanediol in the final step.
3. **Reaction Highlights** Step 1: Achieved a remarkable yield of 89% for γ-butyrolactone. Step 2: Demonstrated an impressive yield of 95.2% for 1,4-butanediol. Step 3: Consistently produced 1,4-butanediol with a yield of 95%.

Figure S39. Relay Catalysis Pathway for 1,4-Butanediol Synthesis. (a) The three-step relay catalysis pathway recommended by the system, visualized in Neo4j. (b) The natural language description of the pathway generated by GPTs.

9. Recommendations for cis-2-Butene Synthesis Pathways

(1) Two-Step Heterogeneous reaction pathway Query and Recommendation

Using Neo4j to perform Cypher queries on the Cat-KG, we obtained all candidate two-step heterogeneous reaction pathways for synthesizing cis-2-butene from Cat-KG, as shown in Figure S40.

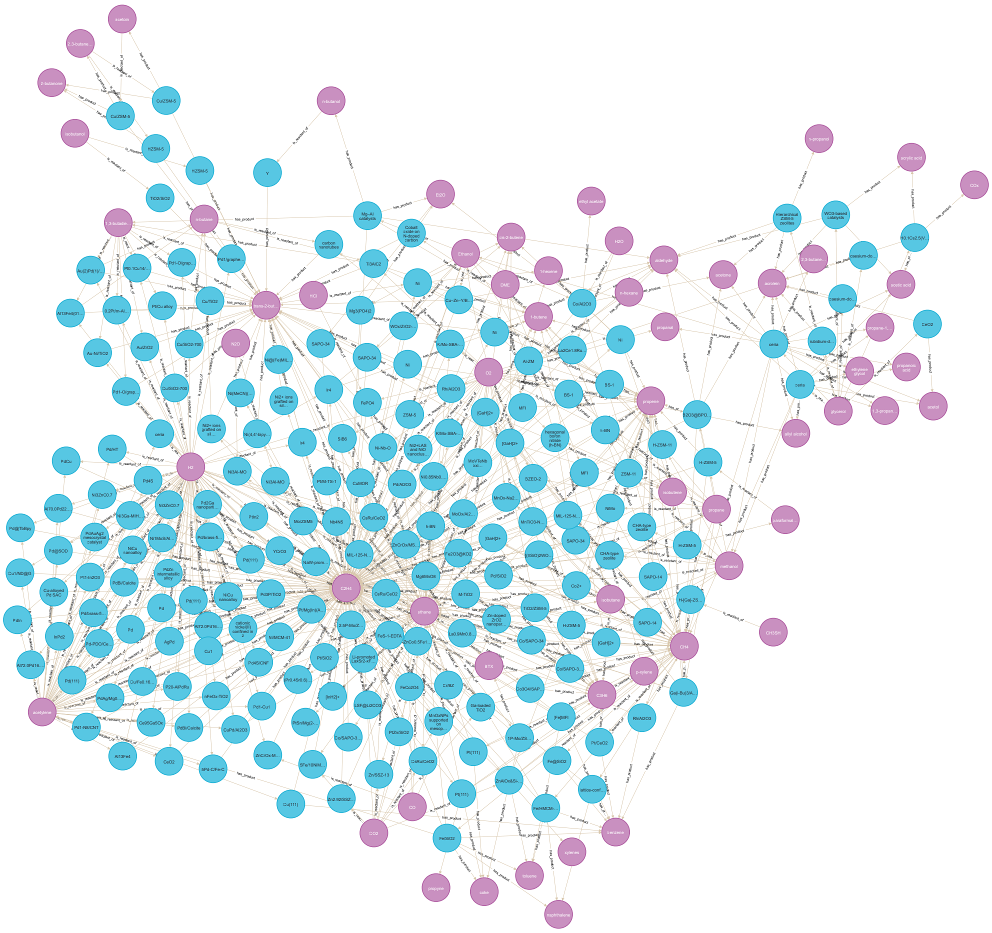

Figure S40. Two-step candidate pathways for synthesizing cis-2-butene. Blue nodes represent reaction nodes with catalyst names above, while purple nodes indicate reactants or products. The lines illustrate the relationships between the compounds and reaction nodes.

Applying our scoring rules, the system recommend a two-step relay catalysis pathway for cis-2-butene synthesis. Figure S41a illustrates this pathway in Neo4j representation. Figure S41b provides the synthesis pathway description converted into natural language by the GPTs.

(a)

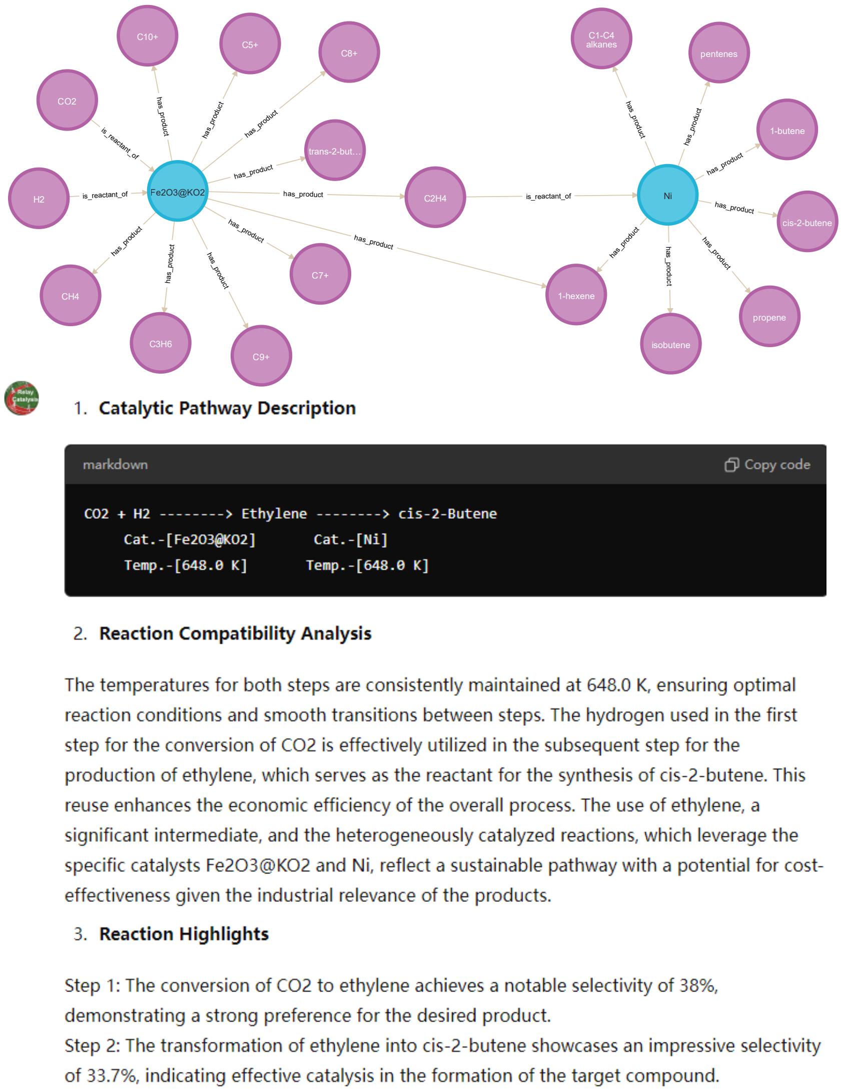

(b)

1. **Catalytic Pathway Description**

markdown Copy code

```
CO2 + H2 --------> Ethylene --------> cis-2-Butene
     Cat.-[Fe2O3@KO2]       Cat.-[Ni]
     Temp.-[648.0 K]       Temp.-[648.0 K]
```

2. **Reaction Compatibility Analysis**

The temperatures for both steps are consistently maintained at 648.0 K, ensuring optimal reaction conditions and smooth transitions between steps. The hydrogen used in the first step for the conversion of CO2 is effectively utilized in the subsequent step for the production of ethylene, which serves as the reactant for the synthesis of cis-2-butene. This reuse enhances the economic efficiency of the overall process. The use of ethylene, a significant intermediate, and the heterogeneously catalyzed reactions, which leverage the specific catalysts Fe2O3@KO2 and Ni, reflect a sustainable pathway with a potential for cost-effectiveness given the industrial relevance of the products.

3. **Reaction Highlights**

Step 1: The conversion of CO2 to ethylene achieves a notable selectivity of 38%, demonstrating a strong preference for the desired product.
Step 2: The transformation of ethylene into cis-2-butene showcases an impressive selectivity of 33.7%, indicating effective catalysis in the formation of the target compound.

Figure S41. Relay Catalysis Pathway for cis-2-Butene Synthesis. (a) The two-step relay catalysis pathway recommended by the system, visualized in Neo4j. (b) The natural language description of the pathway generated by GPTs.

(2) Three-Step Heterogeneous reaction pathway Query and Recommendation

Similarly, we performed a query for three-step heterogeneous reaction pathways for cis-2-butene from Cat-KG, as shown in Figure S42.

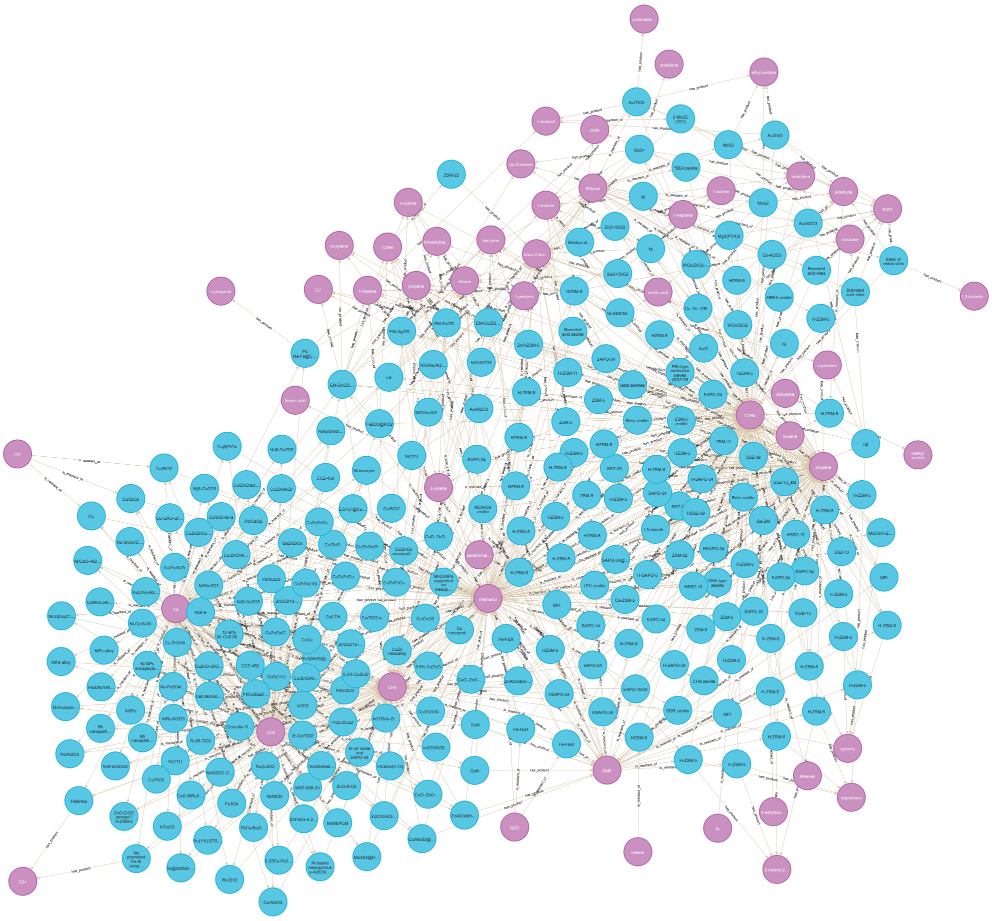

Figure S42. Three-step candidate pathways for synthesizing cis-2-butene. Blue nodes represent reaction nodes with catalyst names above, while purple nodes indicate reactants or products. The lines illustrate the relationships between the compounds and reaction nodes.

Applying our scoring rules, the system recommend a three-step relay catalysis pathway for cis-2-butene synthesis. Figure S43a illustrates this pathway in Neo4j representation. Figure S43b provides the synthesis pathway description converted into natural language by the GPTs.

(a)

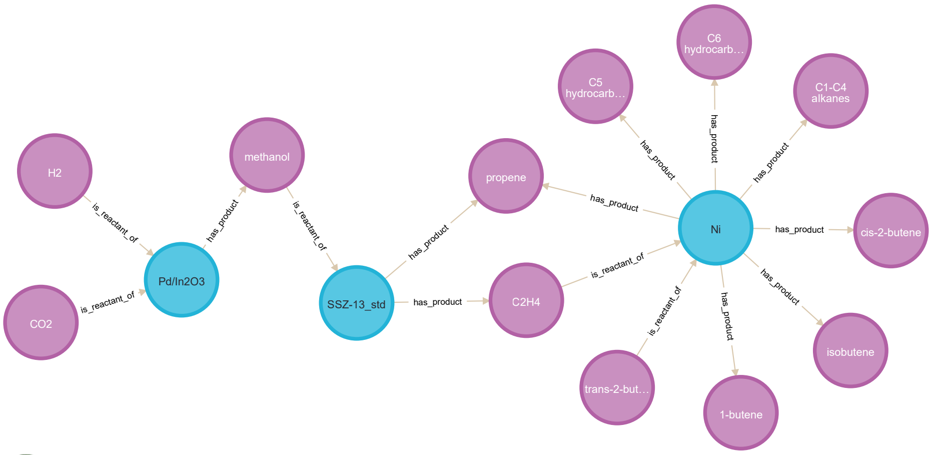

(b)

1. **Catalytic Pathway Description**

```markdown
CO2 + H2 --------> CH3OH --------> C2H4 + C3H6 --------> cis-2-Butene
    Cat.-[Pd/In2O3]      Cat.-[SSZ-13_std]       Cat.-[Ni]
    Temp.-[568.0 K]      Temp.-[623.0 K]       Temp.-[648.0 K]
```

2. **Reaction Compatibility Analysis** The temperatures across the three steps of the catalytic pathway exhibit a sequential increase, with values ranging from 568.0 K to 648.0 K. This gradual rise in temperature ensures that each step remains thermally compatible, facilitating smooth transitions between reactions. Notably, methanol, produced in step 1, serves as a reactant in step 2, enhancing material efficiency by promoting the recycling of intermediates. The economic viability of this pathway is further supported by the utilization of readily available and low-cost raw materials such as CO_2 and H_2, which can potentially enhance sustainability through effective resource management.

3. **Reaction Highlights** Step 1: High selectivity for methanol production at 81%.
   Step 2: The conversion of methanol into ethylene and propylene demonstrates significant potential for generating valuable olefins.
   Step 3: The production of cis-2-butene showcases a commendable selectivity of 33.7%.

Figure S43. Relay Catalysis Pathway for cis-2-Butene Synthesis. (a) The three-step relay catalysis pathway recommended by the system, visualized in Neo4j. (b) The natural language description of the pathway generated by GPTs.

10. Recommendations for 2,5-Furandicarboxylic Acid Synthesis Pathways

(1) Two-Step Heterogeneous reaction pathway Query and Recommendation

Using Neo4j to perform Cypher queries on the Cat-KG, we obtained all candidate two-step heterogeneous reaction pathways for synthesizing 2,5-furandicarboxylic acid (FDCA) from Cat-KG, as shown in Figure S44.

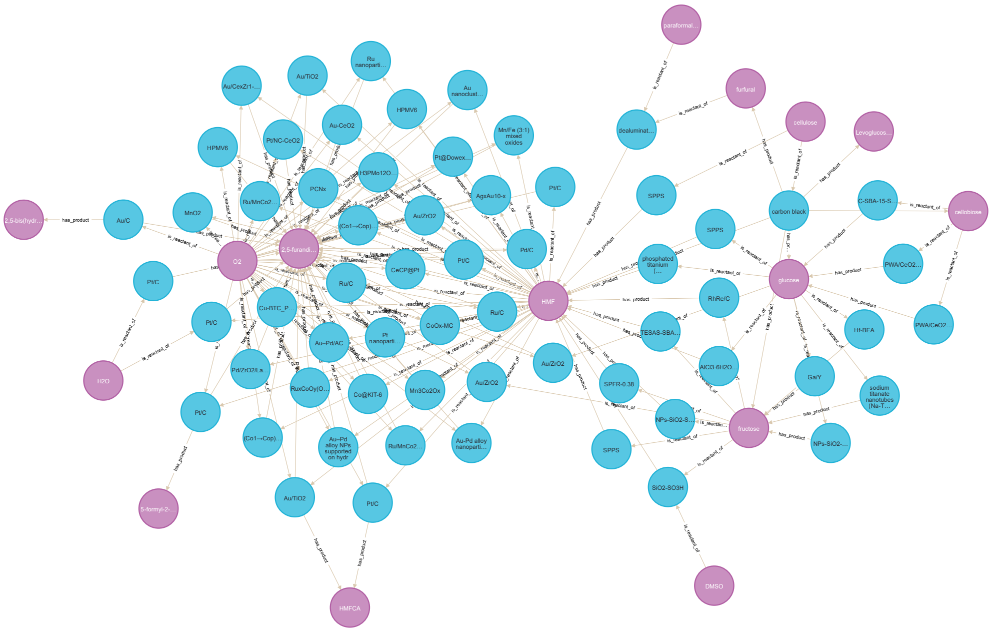

Figure S44. Two-step candidate pathways for synthesizing FDCA. Blue nodes represent reaction nodes with catalyst names above, while purple nodes indicate reactants or products. The lines illustrate the relationships between the compounds and reaction nodes.

Applying our scoring rules, the system recommend a two-step relay catalysis pathway for FDCA synthesis. Figure S45a illustrates this pathway in Neo4j representation. Figure S45b provides the synthesis pathway description converted into natural language by the GPTs.

(a)

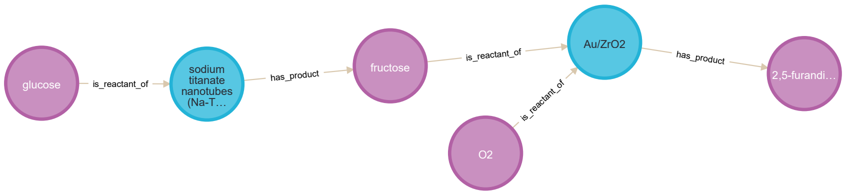

(b)

Sure! Here's the detailed and academic description of the relay catalytic pathway for the synthesis of 2,5-furandicarboxylic acid.

1. **Catalytic Pathway Description**

```markdown
Glucose --------> Fructose --------> 2,5-Furandicarboxylic Acid
      Cat.-[Na-TNTs]               Cat.-[Au/ZrO2]
      Temp.-[373.0 K]              Temp.-[373.0 K]
```

2. **Reaction Compatibility Analysis**

The temperatures for both steps are precisely aligned at 373.0 K, facilitating seamless transitions between the isomerization of glucose to fructose and the subsequent oxidation to 2,5-furandicarboxylic acid. Notably, fructose, generated in step 1, serves as a reactant in step 2, thus promoting the efficient utilization of resources across the pathway. The catalyst sodium titanate nanotubes (Na-TNTs) in step 1 and gold on zirconium oxide (Au/ZrO2) in step 2 are distinct; however, their operational conditions within the same temperature regime support a cohesive reaction sequence. The overall economic efficiency is enhanced through the usage of glucose, a widely available and low-cost biomass-derived feedstock, complemented by the catalytic systems that offer significant potential for reusability in similar reaction contexts.

3. **Reaction Highlights**

Step 1: Achieves a selectivity of 65.26% for the conversion of glucose to fructose.
Step 2: Exhibits an impressive yield of 100% for the synthesis of 2,5-furandicarboxylic acid.

Figure S45. Relay Catalysis Pathway for FDCA Synthesis. (a) The three-step relay catalysis pathway recommended by the system, visualized in Neo4j. (b) The natural language description of the pathway generated by GPTs.

(2) Three-Step Heterogeneous reaction pathway Query and Recommendation

Similarly, we performed a query for three-step heterogeneous reaction pathways for FDCA from Cat-KG, as shown in Figure S46.

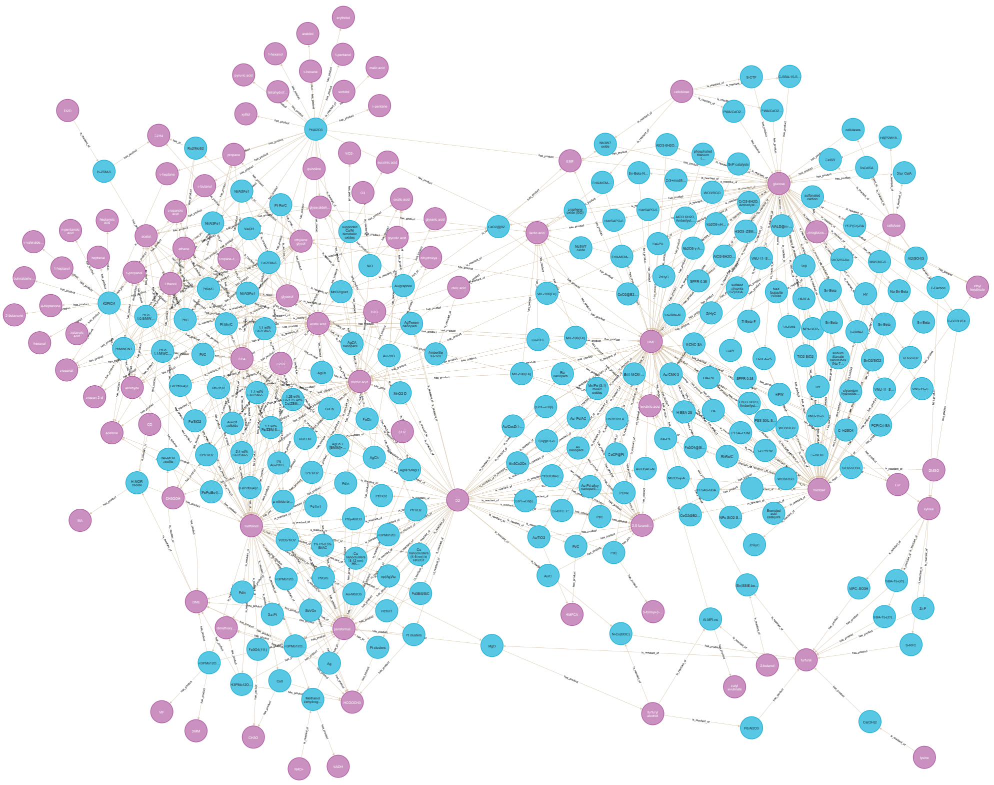

Figure S46. Three-step candidate pathways for synthesizing FDCA. Blue nodes represent reaction nodes with catalyst names above, while purple nodes indicate reactants or products. The lines illustrate the relationships between the compounds and reaction nodes.

Applying our scoring rules, the system recommend a three-step relay catalysis pathway for FDCA synthesis. Figure S47a illustrates this pathway in Neo4j representation. Figure S47b provides the synthesis pathway description converted into natural language by the GPTs.

(a)

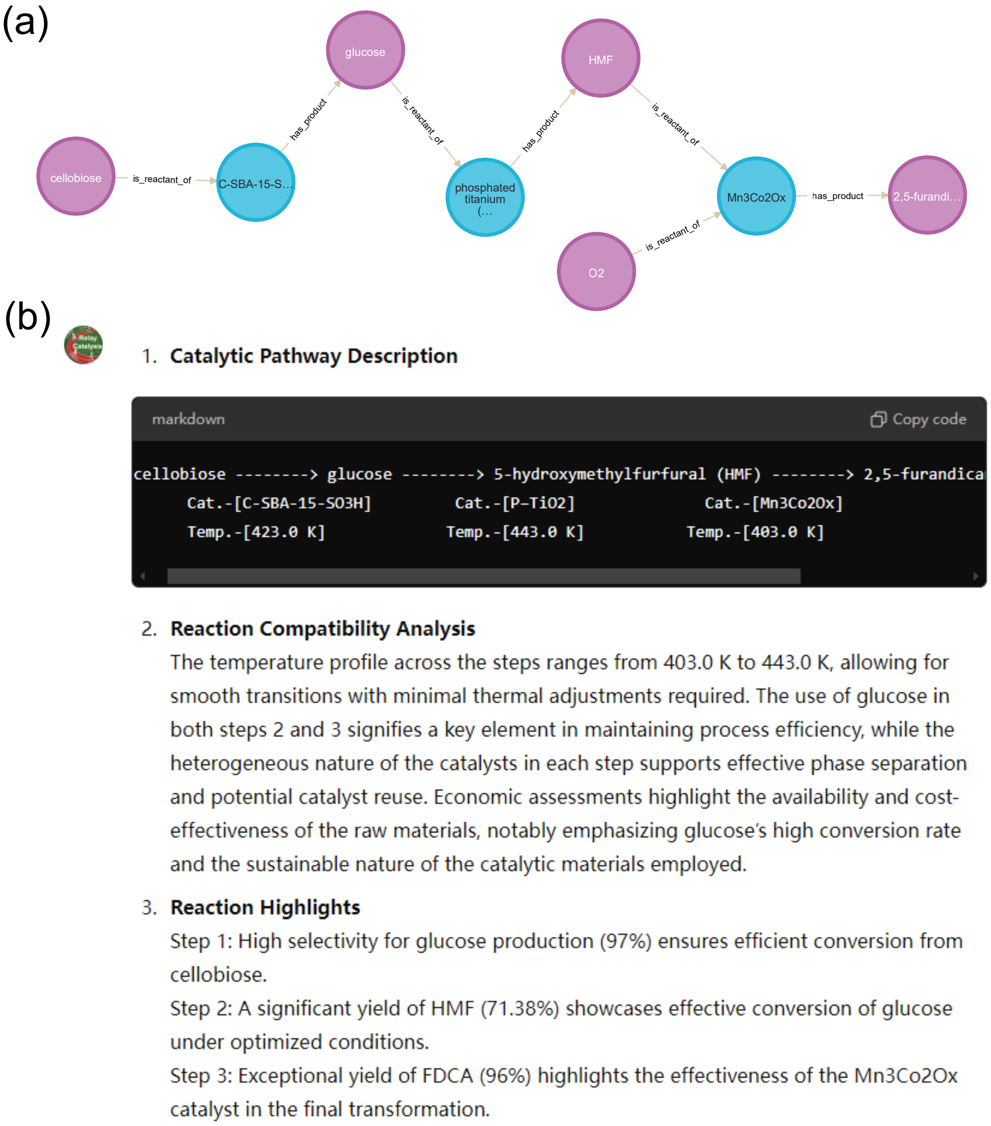

(b)

1. **Catalytic Pathway Description**

markdown   Copy code

```
cellobiose --------> glucose --------> 5-hydroxymethylfurfural (HMF) --------> 2,5-furandica
     Cat.-[C-SBA-15-SO3H]         Cat.-[P-TiO2]            Cat.-[Mn3Co2Ox]
     Temp.-[423.0 K]            Temp.-[443.0 K]          Temp.-[403.0 K]
```

2. **Reaction Compatibility Analysis**
   The temperature profile across the steps ranges from 403.0 K to 443.0 K, allowing for smooth transitions with minimal thermal adjustments required. The use of glucose in both steps 2 and 3 signifies a key element in maintaining process efficiency, while the heterogeneous nature of the catalysts in each step supports effective phase separation and potential catalyst reuse. Economic assessments highlight the availability and cost-effectiveness of the raw materials, notably emphasizing glucose's high conversion rate and the sustainable nature of the catalytic materials employed.

3. **Reaction Highlights**
   Step 1: High selectivity for glucose production (97%) ensures efficient conversion from cellobiose.
   Step 2: A significant yield of HMF (71.38%) showcases effective conversion of glucose under optimized conditions.
   Step 3: Exceptional yield of FDCA (96%) highlights the effectiveness of the Mn3Co2Ox catalyst in the final transformation.

Figure S47. Relay Catalysis Pathway for FDCA Synthesis. (a) The three-step relay catalysis pathway recommended by the system, visualized in Neo4j. (b) The natural language description of the pathway generated by GPTs.

Section S5. Supplementary Figures

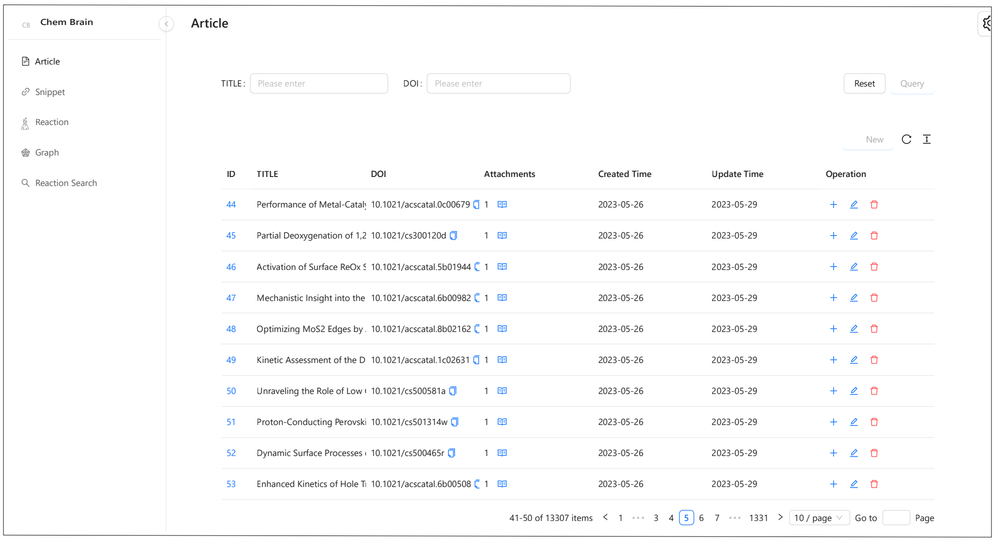

Figure S48. Chem-Brain article management interface. Each entry has details like ID, title, DOI, created time, and update time, and options for operation. The interface facilitates easy access and management of literature, serving as a foundational component for data extraction and processing.

System Instruction Prompt
(Requirements and Automated Extraction Guidance)

```
interface CatalyticStudy {
    is_catalytic_study: boolean;
    num_reactions: number; // It should be noted that the number of reactions in a paper must be based on the actual literature, and can range from 0 to 5. This number cannot be fabricated. If there are more than 5 reactions, only the top 5 most important reactions should be shown. The value of num_reactions should equal the number of reactions listed below.
    reactions: Reaction[];

interface Reaction {
    id: number;
    reactants: string[]; // Compounds cannot be represented by numbers or alphanumerical numbers, such reactions are meaningless
    products: string[]; // Compounds cannot be represented by numbers or alphanumerical numbers, such reactions are meaningless
    catalyst: string;
    reaction_type: 'thermal catalysis' //You must choose between these five options, Reaction type: 'thermal catalysis,' 'photocatalysis,' 'electrocatalysis,' 'enzyme catalysis,' or 'others'
}

}

/**
 * Add reactions to the list below, for example:
 *
 * if (!hasCatalyticContent), export : Reaction_list[] = [
 *     {
 *     is_catalytic_study: false,
 *     num_reactions: 0, // It should be noted that the number of reactions in a paper must be based on the actual literature, and can range from 0 to 5. This number cannot be fabricated. If there are more than 5 reactions, only the top 5 most important reactions should be shown. The value of num_reactions should equal the number of reactions listed below.
 *     reactions: []
 *     }
 * ];
 *
 * Assuming the paper is a catalytic study for demonstration purposes, export const reactions: Reactions_from_article[] = [
 *     {
 *     is_catalytic_study: true,
 *     num_reactions: 2, // The value of num_reactions needs to be equal to the number of reactions below
 *     reactions: [
 *         {
 *             id: 1,
 *             reactants: ["methane", "O2"],
 *             products: ["methanol"],
 *             catalyst: "Pd/C",
 *             description: "Oxidation of methane to methanol using O2 with Pd/C as the catalyst.",
 *             reaction_type: "thermal catalysis"
 *         },
 *         {
 *             id: 2,
 *             reactants: ["furfural", "O2"],
 *             products: ["furoic acid"],
 *             catalyst: "Co SAs/N@C",
 *             reaction_type: "thermal catalysis"
 *         }
 *     ]
 * }
 * ]
 * Note that the above is only an example description, you need to fill in according to the information in the passage
 */

// TODO: export reactions
export const reactions: Reactions_from_article[] = [
]

/**
 * Note:
 * 1. Determine if the paper is focused on catalytic experiments and reactions directly related to the main topic of the study. A valid reaction is not counted if any of the following conditions are met:
 *    a - The paper does not mention any catalytic reactions directly related to the main topic.
 *    b - The paper is primarily focused on computational chemistry, theoretical chemistry, DFT calculations or environmental protection without specific experimental sections.
 *    c - The given title, abstract, and conclusion do not clearly indicate whether actual catalytic experiments were conducted.
 *
 * 2. Note that you only need to provide complete and effective chemical reactions!!! Only include reactions that have complete information about reactants, products, catalysts, and reaction types. Reactions with missing information should not be considered valid for the purpose of this extraction. Additionally, if the reactants or products are described as a general class of compounds or have vague terminology such as "Degradation products," "Aromatic compounds," "Carbonyl compounds," "alkane," etc., and the paper does not specify the exact substances, those reactions should be ignored.
 * 3. An article may contain any number of reactions, and it is necessary to identify catalytic reactions that contain detailed reaction information based on the actual situation
 * 4. Reaction type: 'thermal catalysis,' 'photocatalysis,' 'electrocatalysis,' 'enzyme catalysis,' or 'others.' You must select one of these five options without creating new categories.
 * 5. Special attention should be given to determining if it is related to thermal catalysis.
 * 6. Compounds cannot be represented by numbers or alphanumerical numbers, such reactions are meaningless
 */
```

User Instruction Prompt
(Insert the Targeted Literature Text)

```
{
    "article_information": {
        "title": [
            "Insert the title of the article here"
        ],
        "abstract": [
            "Insert the abstract of the article here"
        ],
        "paragraphs": [
            "Insert the main text paragraphs from the article here"
        ],
        "figure_captions": [
            "Insert figure captions here"
        ],
        "scheme_captions": [
            "Insert scheme captions here"
        ],
        "tables": [
            "Insert table data here"
        ]
    }
}
```

Figure S49. Prompts for Automated Extraction of Catalytic Reaction Overviews. The specialized TypeScript prompts used for automating the extraction of catalytic reaction data. The "System Instruction Prompt" (top section) defines requirements for determining the relevance of the publication, identifying valid reactions, and guiding comprehensive data extraction. The "User Instruction Prompt" (bottom section) outlines placeholders for inputting relevant sections from the literature to be processed.

Catalytic Reaction Overview in JSON Format


```
{
"article_information": {
    "title": [
        "Oxidative coupling of methane—comparisons of MnTiO3–Na2WO4 and MnOx–TiO2–Na2WO4 catalysts on different silica supports"
    ],
    ........
},
"Reaction_Overviews": {
    "Reactions_from_article": [
        {
            "is_catalytic_study": true,
            "num_reactions": 2,
            "reactions": [
                {
                    "id": 1,
                    "reactants": [
                        "CH4",
                        "O2"
                    ],
                    "products": [
                        "C2H4",
                        "C2H6"
                    ],
                    "catalyst": "MnTiO3-NW/SG",
                    "reaction_type": "thermal catalysis"
                },
                {
                    "id": 2,
                    "reactants": [
                        "CH4",
                        "O2"
                    ],
                    "products": [
                        "C2H4",
                        "C2H6"
                    ],
                    "catalyst": "Mn-Ti-NW/SG",
                    "reaction_type": "thermal catalysis"
                }
            ]
        }
    ]
}
```


Figure S50. Catalytic Reaction Overview in JSON Format. The extracted reaction overviews from literature, were structured in JSON format. This format was generated using automated scripts and is presented here to illustrate the systematic representation of the main reactions from the literature. These JSON prompts facilitate automatic parsing and storage for further analysis.

System Instruction Prompt
(Requirements and Automated Extraction Guidance)

```typescript
The following is Typescript code that describe the data structure of reactions. The reactions to be exported are not implement yet.

/**
 * This is a typescript prompt file to export reactions data.
 */

// Interface to represent each reactant in the reaction
interface Reactants {
    reactantName: string; // Compound name or formula
    conversion: string; // Conversion rate of the reactant
    reactantMixtureRatio?: string; // Mixture ratio (e.g., A:B = x:y)
}

// Interface to represent the catalyst used in the reaction
interface Catalyst {
    catalystName: string; // Name or description
    catalystMixtureRatio: string; // Proportional relationship description between the catalyst and the support or catalyst components
    promoters: { // Catalyst promoters
        promoterName: string; // Name or description
        role: string; // Role (Cocatalyst, Additive, etc.)
    }[];
    catalystBETSurfaceArea: string; // BET surface area, number + unit preferred
    turnoverFrequency: string; // Turnover frequency, number + unit preferred
    catalystLoading: string; // Amount of catalyst used
    catalystSupport: string; // Catalyst support material
    catalystShape: string; // Description of catalyst shape and size
    catalystPoreSize: string; // Pore size, number + unit preferred
    catalystPoreVolume: string; // Pore volume, number + unit preferred
    catPreparationMethod: string; // A description of preparation method for the catalyst
    catAcidityAndBasicity: string; // A description of the acidity and basicity for the catalyst
    catMetalOxidationStates: { // Metal oxidation states of the catalyst
        metal: string; // Metal name
        oxidationState: number; // Oxidation state
        proportion: string; // Proportion of the metal
    }[];
}

// Interface to represent the details of the reaction conditions
interface Reaction{
    reactionType: 'thermal catalysis' | 'photocatalysis' | 'electrocatalysis' | 'enzyme catalysis' | 'others'; // Type of this catalytic reaction
    isHeterogeneous: boolean; // Indicates if the reaction is heterogeneous
    balanceGases: string; // Non-reactant gas in the system
    gases:  { // Gases in the reaction
        gasName: string; // Compound name or formula
        pressure: string; // Pressure of the gas
    }[];
    temperature: string; // Temperature for achieving optimal yield
    reactionTime: string; // Reaction time for achieving optimal yield
}

// Interface to represent the solvent used in the reaction
interface Solvents {
    solvents:{ // Solvents in the reaction
        solventName: string; // Compound name or formula
        amount: string; // Amount of the solvent
    }[];
    solventPH: string; // Solvent pH (number preferred)
}

// Interface to represent each product in the reaction
interface Products{
    productName: string; // Product name or formula
    yield: string; // Yield of the product, number + unit preferred
    selectivity: string; // Selectivity of the product, number + unit preferred
}

// Interface to represent the extracted data for the reaction
interface ExtractedData {
    reactants: Reactant[]; // Array of reactants in the reaction
    catalyst: Catalyst; // Catalyst used in the reaction
    reaction: Reaction; // Reaction conditions
    solvents: Solvents[]; // Solvent used in the reaction
    products: Product[]; // Array of products in the reaction
}

// Interface to represent instructions for the extraction task
interface Instructions {
    generalNote: string; // General note for the task
    notes: string[]; // Array of specific notes for the task
}

const instructions: Instructions = {
    generalNote: "Extract the specified reaction from the provided paper. The given description is a reference, and the actual descriptions of reactants, products, and catalysts in the paper may vary in wording and details. Accurately extract and format relevant information to match the specified reaction. The goal is to focus on the reaction of interest.",
    notes: [
        "Use the paper's information to fill the template below. Extract data strictly according to the original description. If any information is missing or not mentioned, retain its key and assign "unknown".",
        "Each reactant or product should be a separate dictionary in the list, with keys 'reactantName', 'conversion' (for reactants), 'productName', 'yield', and 'selectivity' (for products). If mixture ratios are provided, include them as a single string under 'reactantMixtureRatio' in the format 'A:B = x:y' enclosed in single quotes.",
        "'Conversion' is a property of each reactant, while 'yield' and 'selectivity' are properties of each product. Assign these accurately.",
        "H2 and O2 are often key reactants. Ensure these are accurately included.",
        "Default to water (H2O) as the solvent when "solution" or "aqueous solution" is mentioned unless another solvent is specified."
        "For total values: when only a total value (N%) is given for two products (A and B), apply the total to both products. Add an asterisk * after the value to indicate a shared total (eg. selectivity: "8%*").",
        "Preferentially enter 'yield'. If 'yield' is missing but other values indicating quantity or production rate are available, enter those as 'Number + unit'."
    ]
};

/**
 *  export const reactions: ExtractedData[] = [
 *     {
 *         reactants: [
 *             { reactantName: "methane", conversion: "90%"},
 *             { reactantName: "oxygen", conversion: "unknown" },
 *             { reactantMixtureRatio: "CH4:O2 = 1:1"
 *         ],
 *         catalyst: {
 *             catalystName: "palladium on carbon",
 *             catalystMixtureRatio: "5% Pd on carbon",
 *             promoters: [
 *                 { promoterName: "Iron(III) nitrate", role: "Cocatalyst" },
 *                 { promoterName: "Acetic acid", role: "Additive" }
 *             ],
 *             catalystBETSurfaceArea: "1200 m²/g",
 *             turnoverFrequency: "1500 h⁻¹",
 *             catalystLoading: "10 g/L",
 *             catalystSupport: "carbon black",
 *             catalystShape: "Spherical particles， small and well-dispersed NPs in all cases (2.9−3.5 nm)",
 *             catalystPoreSize: "10 nm",
 *             catalystPoreVolume: "0.5 cm³/g",
 *             catPreparationMethod: "incipient wetness impregnation; electrochemical deposition",
 *             catAcidityAndBasicity: "exhibited the highest acidic site concentration at 0.358 mmol/g and a total alkalinity of 0.936 mmol/g.",
 *             catMetalOxidationStates: [
 *                 { metal: "Platinum", oxidationState: "0", proportion: "95%" },
 *                 { metal: "Platinum", oxidationState: "+2", proportion: "unknown" }
 *             ]
 *         },
 *         reaction: {
 *             reactionType: "thermal catalysis",
 *             isHeterogeneous: true,
 *             balanceGases: “air",
 *             gases:[
 *                 {gasName : "O2”, pressure: "2 bar"},
 *                 {gasName : "air”, pressure: "1 atm "}
 *             ],
 *             temperature: "300° C",
 *             reactionTime: "2 hours"
 *         },
 *         solvents: {
 *             solventAmount: :[
 *                 {solventName : " H2O”, amount : "100 mL"},
 *                 {solventName : " Fe(NO3)3”, amount : " 1.0 mM"}
 *                 solventPH: "8"
 *         ],
 *         products: [
 *             { productName: "methanol", yield: "81%", selectivity: "90%" },
 *             { productName: "Product_A", yield: "unknown", selectivity: "8%*" },
 *             { productName: "Product_B", yield: "5%", selectivity: "8%*" }
 *         ]
 *     },
 *  ]
 */

// TODO: export reactions
export const reactions: ExtractedData[] = [
]
```

User Instruction Prompt
(Insert the Reaction Overview to be Extracted and the Targeted Literature Text)

```
Specified reaction:
    {"Insert the Catalytic Reaction Overview here"}
Based on the information in the following paper, you need to extract the information corresponding to the above Specified reaction and complete the code of the reaction:
{
    "article_information": {
        "title": [
            "Insert the title of the article here"
        ],
        "abstract": [
            "Insert the abstract of the article here"
        ],
        "paragraphs": [
            "Insert the main text paragraphs from the article here"
        ],
        "figure_captions": [
            "Insert figure captions here"
        ],
        "scheme_captions": [
            "Insert scheme captions here"
        ],
        "tables": [
            "Insert table data here"
        ]
    }
}
```

Figure S51. Prompts for Automated Extraction of Catalytic Reaction Details. The TypeScript-based prompts define the structure and rules for extracting catalytic reaction data from literature. These prompts streamline the automated extraction process, ensuring accuracy and consistency.

Catalytic Reaction Details in JSON Format


```
"reaction_details": [
    {
        "ID": 1,
        "refined_reaction": {
            "reactants": [
                {
                    "reactantName": "iodobenzene",
                    "conversion": "unknown"
                },
                {
                    "reactantName": "NaOH",
                    "conversion": "unknown"
                }
            ],
            "catalyst": {
                "catalystName": "Cu–ZnO–ZrO2",
                "catalystMixtureRatio": "unknown",
                "promoters": [],
                "catalystBETSurfaceArea": "77.5 m²/g",
                "turnoverFrequency": "unknown",
                "catalystLoading": "1.24 mol%",
                "catalystSupport": "ZnO–ZrO2",
                "catalystShape": "unknown",
                "catalystPoreSize": "6.32 nm",
                "catalystPoreVolume": "unknown",
                "catPreparationMethod": "in situ coprecipitation",
                "catAcidityAndBasicity": "unknown",
                "catMetalOxidationStates": [
                    {
                        "metal": "Copper",
                        "oxidationState": 0,
                        "proportion": "unknown"
                    },
                    {
                        "metal": "Copper",
                        "oxidationState": 1,
                        "proportion": "unknown"
                    }
                ]
            },
            "reaction": {
                "reactionType": "thermal catalysis",
                "isHeterogeneous": true,
                "balanceGases": "Air",
                "pressure":[
                    {
                        "gasName": "air",
                        "pressure": "1 atm"
                    }
                ],
                "temperature": "110 ° C",
                "reactionTime": "24 hours"
            },
            "solvents": {
                "solventAmount": ":[
                    {
                        "solventName": "DMSO",
                        "amount": " 2.0 mL“
                    },
                    {
                        "solventName": "H2O",
                        "amount": "2.0 mL"
                    }
                ],
                "solventPH": "unknown"
            },
            "products": [
                {
                    "productName": "phenol",
                    "yield": "99%",
                    "selectivity": "unknown"
                }
            ]
        }
    }
]
```


Figure S52. Catalytic Reaction Details in JSON Format. The detailed representation of catalytic reactions is structured in JSON format. The data include reactants, catalysts, reaction conditions, and products, as well as various attributes such as catalyst surface area, loading, support, and oxidation states. This structured format ensures accuracy and completeness, facilitating automated extraction and data organization.

Cypher Query Example

```
MATCH (s:substance {cid_name: 'Crotonaldehyde'})
MATCH (s)<-[r:has_product]-(m:reactions_thermalcatalyst)
WHERE m.temperature_K IS NOT NULL
  AND m.isHeterogeneous = 'true'
MATCH (m)<-[r1:is_reactant_of]-(s1:substance)
MATCH (s1)<-[r2:has_product]-(m1:reactions_thermalcatalyst)
WHERE m1.temperature_K IS NOT NULL
  AND m1.isHeterogeneous = 'true'
MATCH (m1)<-[r3:is_reactant_of]-(s2:substance)
MATCH (s2)<-[r4:has_product]-(m2:reactions_thermalcatalyst)
WHERE m2.temperature_K IS NOT NULL
  AND m2.isHeterogeneous = 'true'
  AND 'H2' IN m2.reactantnames
  AND 'CO' IN m2.reactantnames
MATCH (m2)<-[r5:is_reactant_of]-(s3:substance)
RETURN [r.score, r2.score, r4.score] AS score,
    [m, m1, m2] AS reaction,
    [s1.name, s2.name, m2.reactantnames] AS path,
    [r4.selectivity, r3.conversion, r2.selectivity, r1.conversion, r.selectivity, r.yield] AS relation
```

Figure S53. Cypher Query for Pathway Search in Cat-KG. The Cypher query identifies relevant reactions by first locating the target compound in Cat-KG and then matching it to appropriate reactions. This example demonstrates a three-step pathway query where CO and H_2 are required reactants, with crotonaldehyde as the target product.

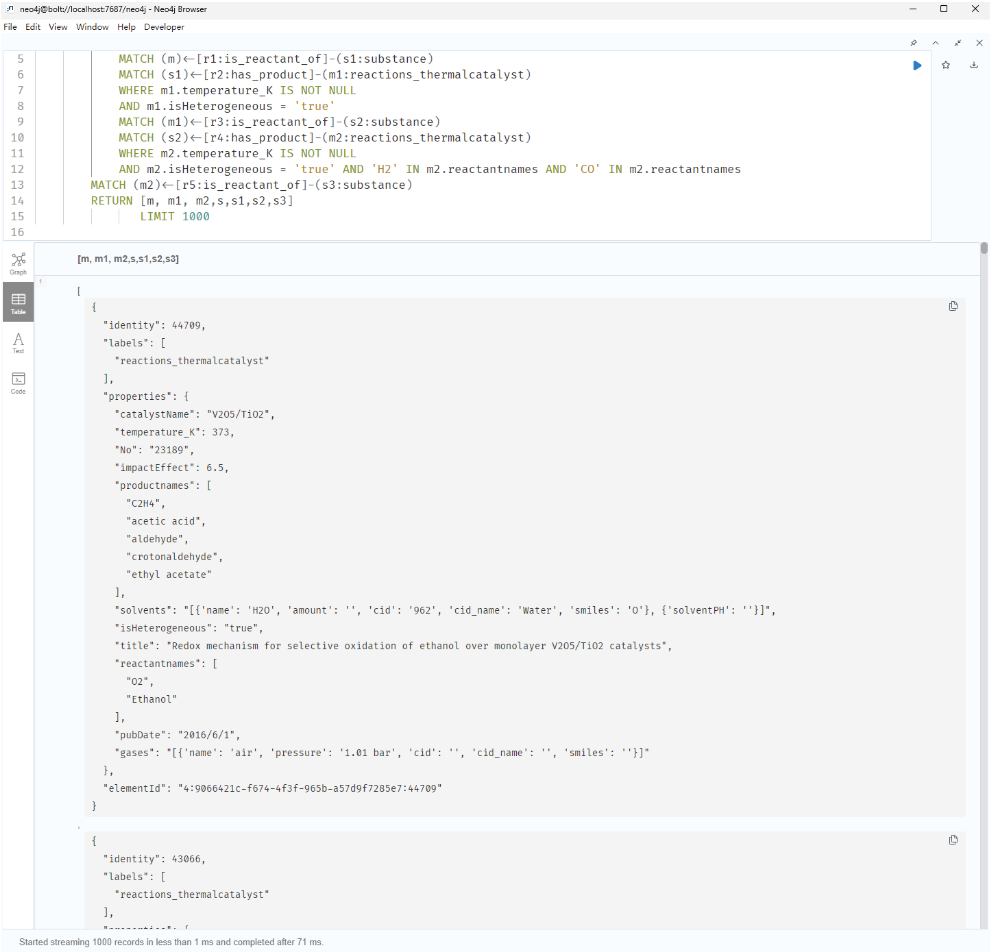

Figure S54. Text-Based Output of Pathway Search Results in Cat-KG. The pathway search results are displayed in text format, providing detailed information for each reaction step. The text-based format allows researchers to analyze potential pathways automatically and efficiently, offering a clear and organized overview of the data.

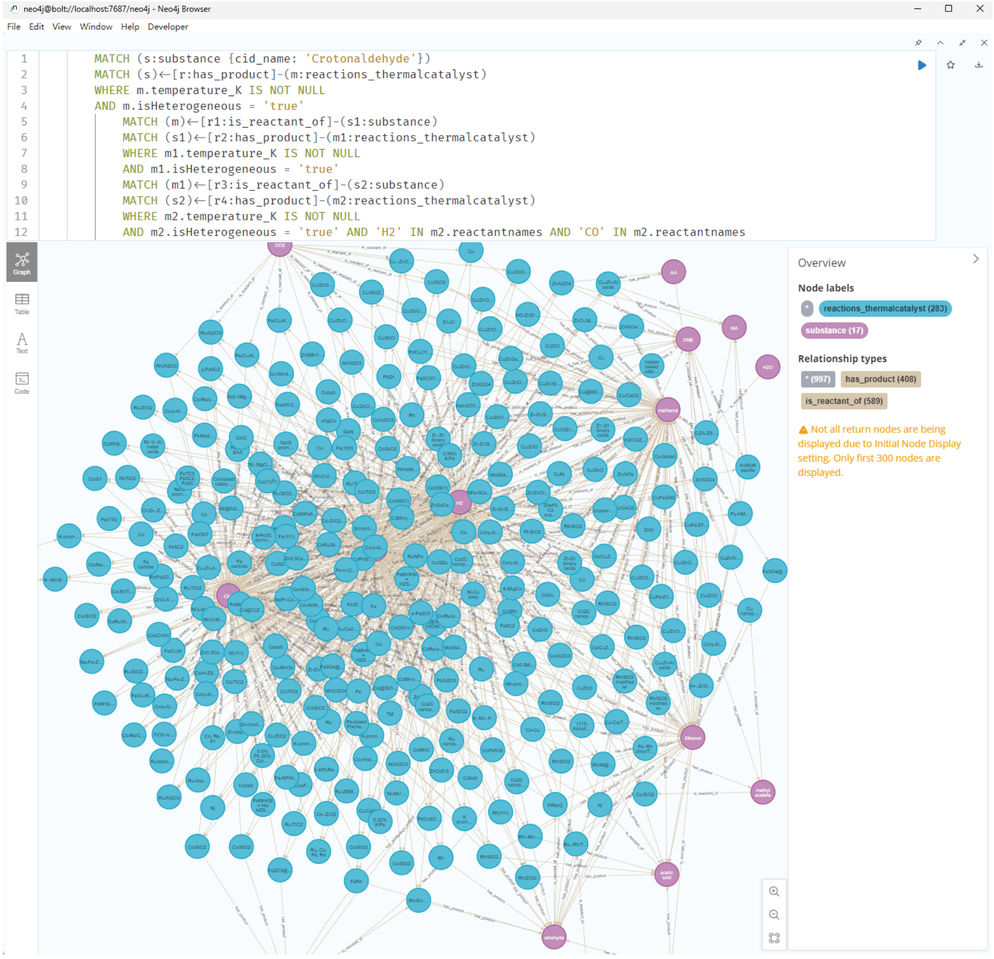

Figure S55. Visualization of Pathway Search Results in Neo4j. The pathways generated from the Cypher query are visualized in the Neo4j interface. The nodes represent substances and reactions, while the relationships between them denote reactant-product interactions. This graphical format helps users explore complex reaction pathways and provides an intuitive overview of reaction processes.

Score and Filter Top-Ranked Candidate Pathways

path1: {score:3628.2, pathway:CO2 + H2 ----> CH4(sele.100%) ----> Ethanol(sele.98.30%) ----> Crotonaldehyde
step_1: CO2 + H2 ---> CH4 (id:23955, Catalyst:Ni NPs encapsulated in MIL-101, Temp:573K, Title:Enhancing activity for carbon dioxide methanation by encapsulating (1 1 1) facet Ni particle in metal–organic frameworks at low temperature)
step_2: H2 + CO + CH4 ---> Ethanol + methanol + acetic acid (id:10647, Catalyst:Cu-Co/TiO2, Temp:573K, Title:Efficient Synthesis of Ethanol from CH4 and Syngas on a Cu-Co/TiO2 Catalyst Using a Stepwise Reactor)
step_3: Ethanol ---> furan + 2-butenol + C2H4 + benzene + n-butanol + 1-butene + aldehyde + crotonaldehyde + ethyl acetate + Et2O (id:18697, Catalyst:Au/TiO2, Temp:573K, Title:Difference in the deactivation of Au catalysts during ethanol transformation when supported on ZnO and on TiO2)
}

path2: {score:3397.97, pathway:H2 + CO ----> CH4 ----> Ethanol(sele.98.30%) ----> Crotonaldehyde
step_1: H2 + CO ---> CH4 (id:23974, Catalyst:Ni–Cr–Al, Temp:573K, Title:CO methanation on ordered mesoporous Ni–Cr–Al catalysts: Effects of the catalyst structure and Cr promoter on the catalytic properties)
step_2: H2 + CO + CH4 ---> Ethanol + methanol + acetic acid (id:10647, Catalyst:Cu-Co/TiO2, Temp:573K, Title:Efficient Synthesis of Ethanol from CH4 and Syngas on a Cu-Co/TiO2 Catalyst Using a Stepwise Reactor)
step_3: Ethanol ---> furan + 2-butenol + C2H4 + benzene + n-butanol + 1-butene + aldehyde + crotonaldehyde + ethyl acetate + Et2O (id:18697, Catalyst:Au/TiO2, Temp:573K, Title:Difference in the deactivation of Au catalysts during ethanol transformation when supported on ZnO and on TiO2)
}

path3: {score:3368.34, pathway:CO2 + H2 ----> CH4(sele.100%) ----> Ethanol(sele.98.30%) ----> Crotonaldehyde
step_1: CO2 + H2 ---> CH4 (id:18664, Catalyst:Ni/Al2O3–CeO2, Temp:593K, Title:Ni nanocatalysts supported on mesoporous Al2O3–CeO2 for CO2 methanation at low temperature)
step_2: H2 + CO + CH4 ---> Ethanol + methanol + acetic acid (id:10647, Catalyst:Cu-Co/TiO2, Temp:573K, Title:Efficient Synthesis of Ethanol from CH4 and Syngas on a Cu-Co/TiO2 Catalyst Using a Stepwise Reactor)
step_3: Ethanol ---> furan + 2-butenol + C2H4 + benzene + n-butanol + 1-butene + aldehyde + crotonaldehyde + ethyl acetate + Et2O (id:18697, Catalyst:Au/TiO2, Temp:573K, Title:Difference in the deactivation of Au catalysts during ethanol transformation when supported on ZnO and on TiO2)
}

path4: {score:3251.41, pathway:Ethanol ----> aldehyde ----> acetaldol ----> Crotonaldehyde
step_1: Ethanol ---> CH3COCH3 + C2H4 + CH4 + aldehyde (id:21227, Catalyst:Co/CeO2, Temp:693K, Title:Co/CeO2 and Ni/CeO2 catalysts for ethanol steam reforming: Effect of the cobalt/nickel dispersion on catalysts properties)
step_2: aldehyde ---> acetaldol + C2H4 + 1,3-butadiene (id:20239, Catalyst:Mg-Al catalysts, Temp:698K, Title:Investigation into 1,3-butadiene and other bulk chemicals' formation from bioethanol over Mg–Al catalysts)
step_3: acetaldol ---> C2H4 + 1,3-butadiene + n-butanol + 1-butene + aldehyde + crotonaldehyde + Et2O (id:20240, Catalyst:Mg-Al catalysts, Temp:698K, Title:Investigation into 1,3-butadiene and other bulk chemicals' formation from bioethanol over Mg–Al catalysts)
}

path5: {score:3211.15, pathway:CO2 + H2 ----> CH4(sele.100%) ----> Ethanol(sele.98.30%) ----> Crotonaldehyde
step_1: CO2 + H2 ---> CH4 (id:23955, Catalyst:Ni NPs encapsulated in MIL-101, Temp:573K, Title:Enhancing activity for carbon dioxide methanation by encapsulating (1 1 1) facet Ni particle in metal–organic frameworks at low temperature)
step_2: H2 + CO + CH4 ---> Ethanol + methanol + acetic acid (id:10647, Catalyst:Cu-Co/TiO2, Temp:573K, Title:Efficient Synthesis of Ethanol from CH4 and Syngas on a Cu-Co/TiO2 Catalyst Using a Stepwise Reactor)
step_3: Ethanol + aldehyde ---> n-butanol + butyraldehyde + crotonaldehyde (id:24103, Catalyst:MgO, Temp:673K, Title:Computational and Experimental Mechanistic Insights into the Ethanol-to-Butanol Upgrading Reaction over MgO)
}

path6: {score:3136.54, pathway:CO2 + H2 ----> Ethanol(conv.61%) ----> aldehyde ----> Crotonaldehyde
step_1: CO2 + H2 ---> Ethanol + formic acid + methanol + acetic acid + CH4 + propane + paraformaldehyde + ethane (id:7961, Catalyst:Ni(111), Temp:573K, Title:Quantifying the Impact of Parametric Uncertainty on Automatic Mechanism Generation for CO2 Hydrogenation on Ni(111))
step_2: Ethanol ---> aldehyde (id:19069, Catalyst:Cu/AlMgO, Temp:573K, Title:Ethanol dehydrogenative reactions catalyzed by copper supported on porous Al–Mg mixed oxides)
step_3: aldehyde ---> crotonaldehyde (id:28217, Catalyst:HAP, Temp:598K, Title:Direct, Selective Production of Aromatic Alcohols from Ethanol Using a Tailored Bifunctional Cobalt–Hydroxyapatite Catalyst)
}
......
......

Figure S56. Top-Ranked Candidate Pathways for Crotonaldehyde Synthesis. Top-ranked candidate pathways for crotonaldehyde synthesis are scored and filtered using Python-based scoring rules. The evaluation considers factors such as reaction source reliability, catalyst compatibility, atom economy, and other key criteria. Scores, selectivity percentages, reaction conditions, catalyst details, and the corresponding literature sources are provided, enabling users to quickly identify pathways suitable for further experimental study.

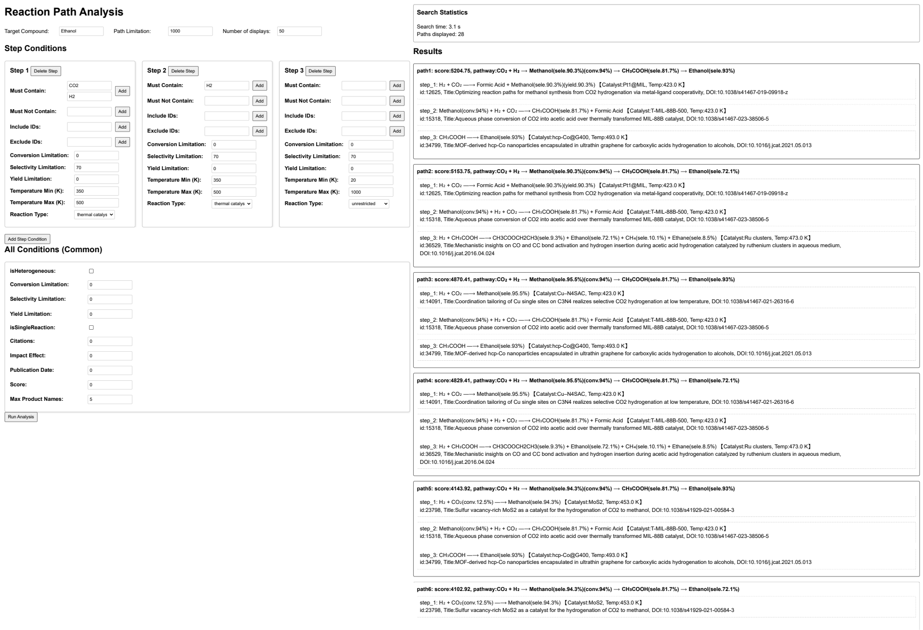

Figure S57. Catalytic Pathway Query Interface. The left panel allows users to input query constraints, including target product, number of desired pathways to return, inclusion or exclusion of specific reactants, reaction conditions, and maximum pathway length. The right panel displays the retrieved candidate reaction pathways queried from Cat-KG using automatically generated Cypher statements. The resulting pathways are scored, ranked, and presented in an interpretable format to assist researchers in identifying high-potential relay strategies that match their design requirements.

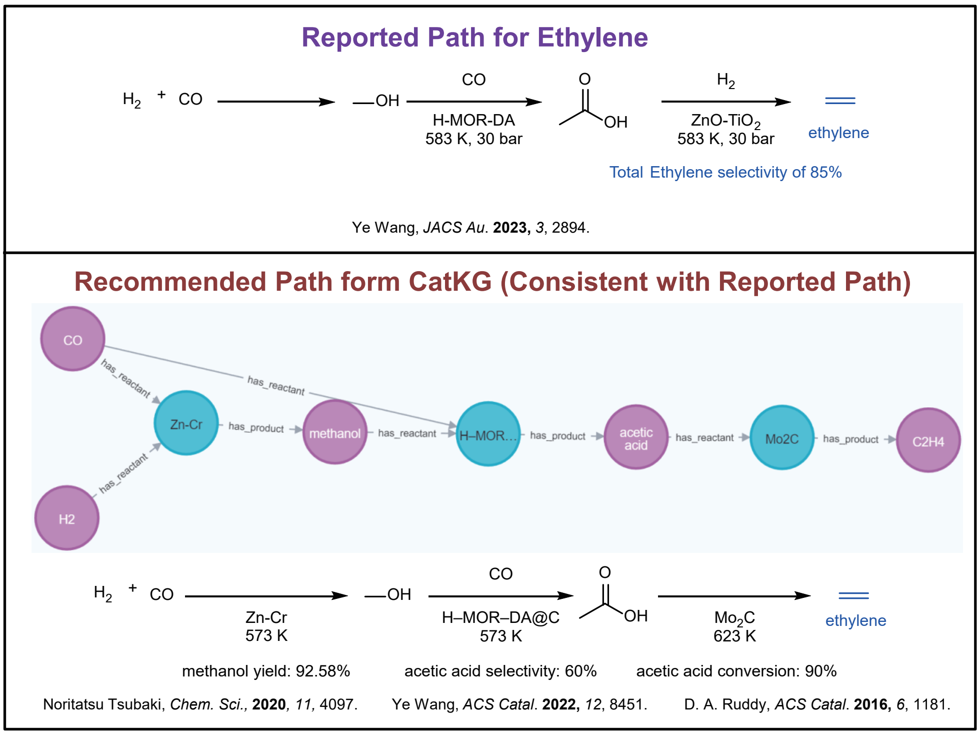

Figure S58. Comparison between the reported and KG-based recommended relay catalysis pathways for ethylene synthesis. The upper part of the figure illustrates a three-step reported pathway for the synthesis of ethylene[17]. The lower part shows the corresponding pathway recommended by the KG-based system, starting from methanol production from syngas [18], followed by the carbonylation of methanol and CO to form acetic acid [19], and concluding with the hydrogenation of acetic acid to ethylene [20].

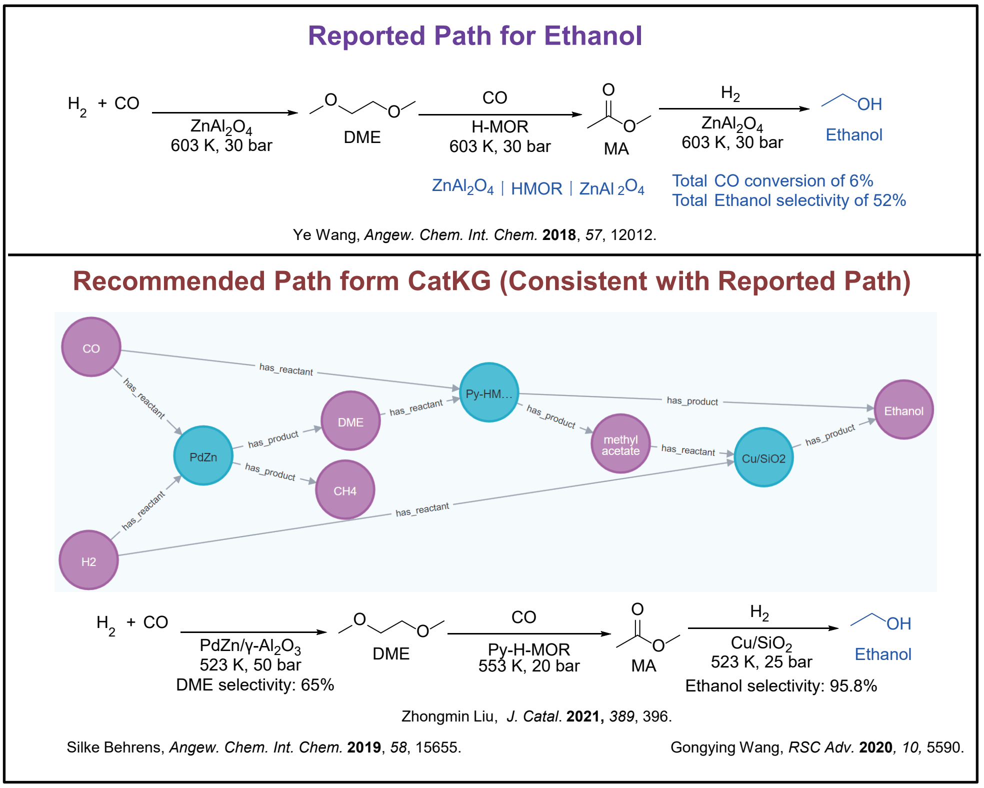

Figure S59. Comparison between the reported and KG-based recommended relay catalysis pathways for ethanol synthesis. The upper part of the figure illustrates a three-step reported pathway for the synthesis of ethanol, which has been experimentally validated as an effective relay catalysis pathway [21]. The lower part presents the corresponding pathway recommended by the KG-based system, starting from syngas conversion to 1,2-dimethoxyethane (DME) [22], followed by the transformation of DME to methyl acetate (MA) [23], and concluding with the hydrogenation of MA to ethanol [24].

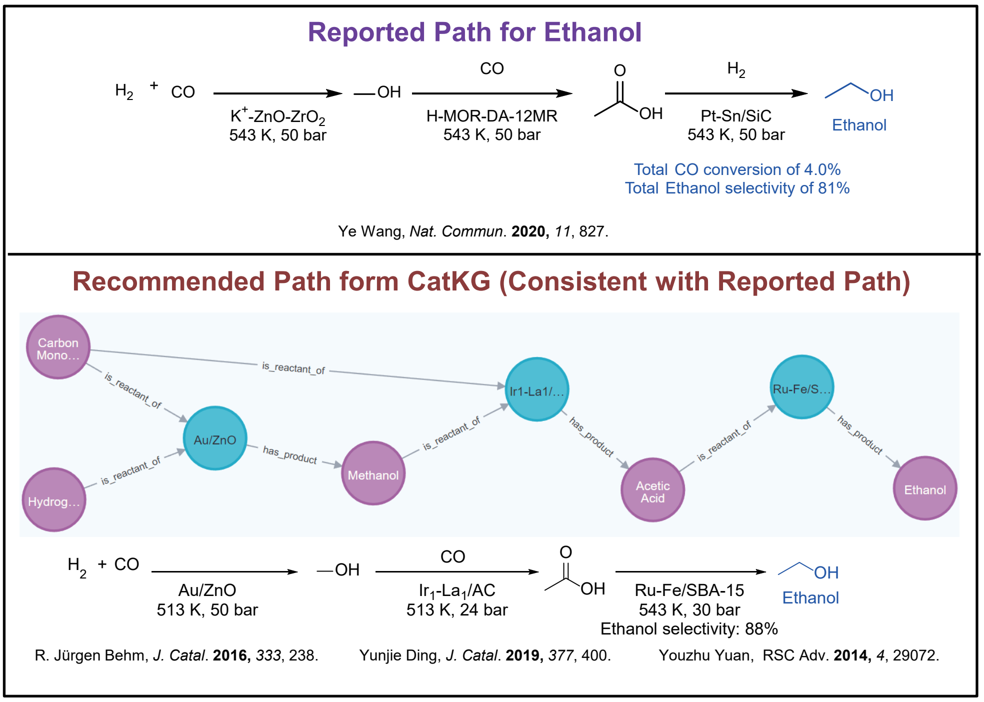

Figure S60. Comparison between the reported and KG-based recommended relay catalysis pathways for ethanol synthesis. The upper part of the figure illustrates a three-step reported pathway for ethanol production via syngas to methanol, followed by methanol carbonylation to acetic acid, and subsequent hydrogenation to ethanol [25]. The lower part shows the corresponding pathway recommended by the KG-based system, which also follows a syngas-to-ethanol sequence via methanol [26], methanol to acetic acid [27], and acetic acid hydrogenation to ethanol [28].

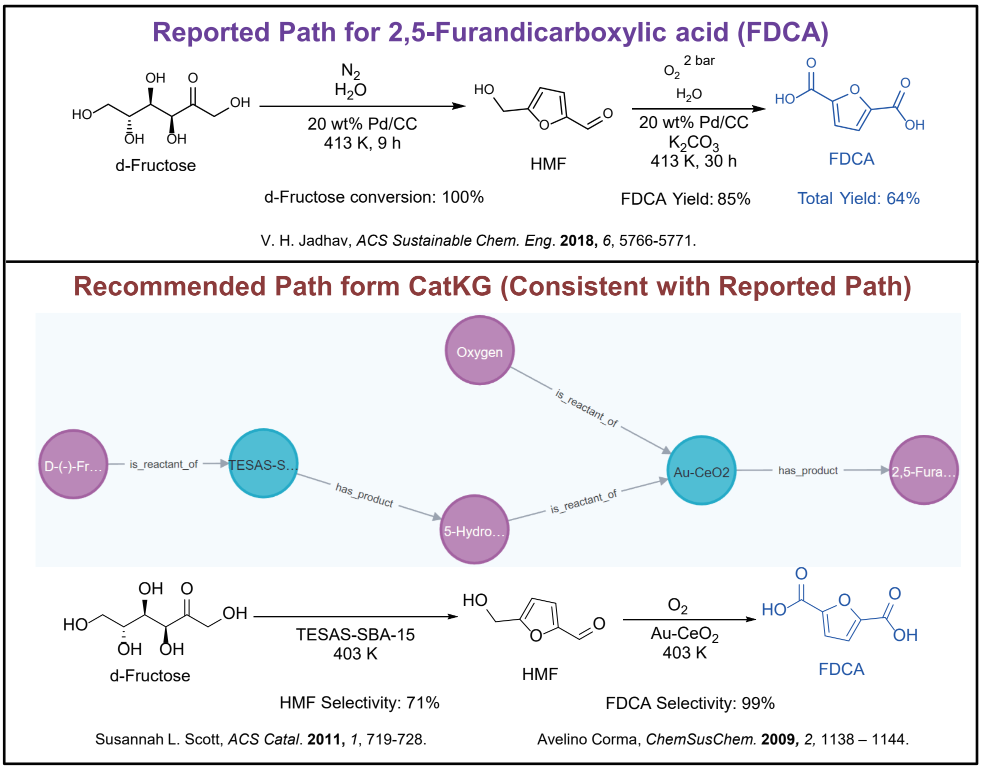

Figure S61. Comparison between the reported and KG-based recommended relay catalysis pathways for FDCA synthesis. The upper part of the figure displays a two-step reported pathway for the synthesis of FDCA [29]. The lower part shows the corresponding pathway recommended by the KG-based system, which involves the conversion of D-fructose to HMF [30], followed by the oxidation of HMF to FDCA [31].

References

[1] Weber, J. M., Guo, Z., Zhang, C., Schweidtmann, A. M. & Lapkin, A. A. Chemical data intelligence for sustainable chemistry. *Chem Soc Rev* **50**, 12013–12036 (2021). URL DOIhttps://doi.org/10.1039/D1CS00477H.

[2] Kearnes, S. M. *et al.* The open reaction database. *J Am Chem Soc* **143**, 18820–18826 (2021). URL https://doi.org/10.1021/jacs.1c09820.

[3] Hastings, J. *et al.* Chebi in 2016: Improved services and an expanding collection of metabolites. *Nucleic Acids Res* **44**, D1214–D1219 (2016). URL https://doi.org/10.1093/nar/gkv1031.

[4] Guo, J. *et al.* Automated chemical reaction extraction from scientific literature. *J Chem Inf Model* **62**, 2035–2045 (2022). URL https://doi.org/10.1021/acs.jcim.1c00284.

[5] Zheng, X., Wang, B., Zhao, Y., Mao, S. & Tang, Y. A knowledge graph method for hazardous chemical management: Ontology design and entity identification. *Neurocomputing* **430**, 104–111 (2021).

[6] Francis, N. *et al.* *Cypher: An evolving query language for property graphs*, 1433–1445 (2018).

[7] Goutte, C. & Gaussier, E. Losada, D. E. & Fernández-Luna, J. M. (eds) *A probabilistic interpretation of precision, recall and f-score, with implication for evaluation.* (eds Losada, D. E. & Fernández-Luna, J. M.) *Advances in Information Retrieval*, 345–359 (Springer Berlin Heidelberg, Berlin, Heidelberg, 2005).

[8] Huang, D. & Cole, J. M. A database of thermally activated delayed fluorescent molecules auto-generated from scientific literature with chemdataextractor. *Sci Data* **11**, 80 (2024). URL https://doi.org/10.1038/s41597-023-02897-3.

[9] Ji, S., Pan, S., Cambria, E., Marttinen, P. & Yu, P. S. A survey on knowledge graphs: Representation, acquisition, and applications. *IEEE T Neur Net Lear* **33**, 494–514 (2022).

[10] Alam, M., Asefa Gesese, G. & Paris, P.-H. J. a. e.-p. Neurosymbolic methods for dynamic knowledge graphs. *arXiv:2409.04572* (2024). URL https://ui.adsabs.harvard.edu/abs/2024arXiv240904572A.

[11] Yang, Z., Yuan, S., Shao, Z., Li, W. & Liu, R. A review on synergizing knowledge graphs and large language models. *Computing* **107**, 143 (2025). URL https://doi.org/10.1007/s00607-025-01499-8.

[12] Wang, X. *et al.* Knowledge graph quality control: A survey. *Fund Res-China* **1** (2021).

[13] Guellil, I., Garcia-Dominguez, A., Lewis, P. R., Hussain, S. & Smith, G. Entity linking for english and other languages: a survey. *Knowl Inf Syst* **66**, 3773–3824 (2024). URL https://doi.org/10.1007/s10115-023-02059-2.

[14] Zhu, B., Wang, R., Wang, J., Shao, F. & Wang, K. A survey: knowledge graph entity alignment research based on graph embedding. *Artif Intell Rev* **57**, 229 (2024). URL https://doi.org/10.1007/s10462-024-10866-4.

[15] Mirza, A. *et al.* A framework for evaluating the chemical knowledge and reasoning abilities of large language models against the expertise of chemists. *Nat Chem* (2025). URL https://doi.org/10.1038/s41557-025-01815-x.

[16] Li, H. *et al.* Beyond chemical qa: Evaluating llm's chemical reasoning with modular chemical operations. *arXiv:2505.21318* (2025). URL https://ui.adsabs.harvard.edu/abs/2025arXiv250521318L.

[17] Chen, K. *et al.* Relay catalysis for highly selective conversion of methanol to ethylene in syngas. *JACS Au* **3**, 2894–2904 (2023). URL https://doi.org/10.1021/jacsau.3c00463.

[18] Tan, L. *et al.* Design of a core–shell catalyst: an effective strategy for suppressing side reactions in syngas for direct selective conversion to light olefins. *Chem Sci* **11**, 4097–4105 (2020). URL http://dx.doi.org/10.1039/C9SC05544D.

[19] Zhang, F. *et al.* Selective transformation of methanol to ethanol in the presence of syngas over composite catalysts. *Acs Catal* **12**, 8451–8461 (2022). URL https://doi.org/10.1021/acscatal.2c01725.

[20] Schaidle, J. A. *et al.* Experimental and computational investigation of acetic acid deoxygenation over oxophilic molybdenum carbide: Surface chemistry and active site identity. *Acs Catal* **6**, 1181–1197 (2016). URL https://doi.org/10.1021/acscatal.5b01930.

[21] Zhou, W. *et al.* Direct conversion of syngas into methyl acetate, ethanol, and ethylene by relay catalysis via the intermediate dimethyl ether. *Angew Chem Int Ed* **57**, 12012–12016 (2018). URL https://onlinelibrary.wiley.com/doi/abs/10.1002/anie.201807113.

[22] Gentzen, M. *et al.* Supported intermetallic pdzn nanoparticles as bifunctional catalysts for the direct synthesis of dimethyl ether from co-rich synthesis gas. *Angew Chem Int Ed* **58**, 15655–15659 (2019). URL https://onlinelibrary.wiley.com/doi/abs/10.1002/anie.201906256.

[23] Zhou, Z. *et al.* Direct conversion of dimethyl ether and co to acetone via coupling carbonylation and ketonization. *J Catal* **396**, 360–373 (2021). URL https://dx.doi.org/10.1016/j.jcat.2021.03.006.

[24] Ren, Z. *et al.* Highly active ce, y, la-modified cu/sio2 catalysts for hydrogenation of methyl acetate to ethanol. *Rsc Adv* **10**, 5590–5603 (2020). URL https://dx.doi.org/10.1039/C9RA08780J.

[25] Kang, J. *et al.* Single-pass transformation of syngas into ethanol with high selectivity by triple tandem catalysis. *Nat Commun* **11**, 827 (2020). URL https://doi.org/10.1038/s41467-020-14672-8.

[26] Hartadi, Y., Widmann, D. & Behm, R. J. Methanol formation by co2 hydrogenation on au/zno catalysts – effect of total pressure and influence of co on the reaction characteristics. *J Catal* **333**, 238–250 (2016). URL https://dx.doi.org/10.1016/j.jcat.2015.11.002.

[27] Feng, S. *et al.* Insight into the stability of binuclear ir–la catalysts for efficient heterogeneous methanol carbonylation. *J Catal* **377**, 400–408 (2019). URL https://dx.doi.org/10.1016/j.jcat.2019.06.050.

[28] Li, W. *et al.* Efficient ru–fe catalyzed selective hydrogenolysis of carboxylic acids to alcoholic chemicals. *RSC Adv* **4**, 29072–29082 (2014). URL https://dx.doi.org/10.1039/C4RA03201B.

[29] Rathod, P. V. & Jadhav, V. H. Efficient method for synthesis of 2,5-furandicarboxylic acid from 5-hydroxymethylfurfural and fructose using pd/cc catalyst under aqueous conditions. *Acs Sustain Chem Eng* **6**, 5766–5771 (2018). URL https://doi.org/10.1021/acssuschemeng.7b03124.

[30] Crisci, A. J. *et al.* Acid-functionalized sba-15-type silica catalysts for carbohydrate dehydration. *Acs Catal* **1**, 719–728 (2011). URL https://doi.org/10.1021/cs2001237.

[31] Casanova, O., Iborra, S. & Corma, A. Biomass into chemicals: Aerobic oxidation of 5-hydroxymethyl-2-furfural into 2,5-furandicarboxylic acid with gold nanoparticle catalysts. *Chemsuschem* **2**, 1138–1144 (2009). URL https://chemistry-europe.onlinelibrary.wiley.com/doi/abs/10.1002/cssc.200900137.
